# Preliminary clinical and cost effectiveness of augmented depression therapy versus cognitive behavioural therapy for the treatment of anhedonic depression (ADepT): a single-centre, open-label, parallel-group, pilot, randomised, controlled trial

**DOI:** 10.1016/j.eclinm.2023.102084

**Published:** 2023-07-13

**Authors:** Barnaby D. Dunn, Emily Widnall, Laura Warbrick, Faith Warner, Nigel Reed, Alice Price, Merle Kock, Clara Courboin, Rosie Stevens, Kim Wright, Nicholas J. Moberly, Nicole Geschwind, Christabel Owens, Anne Spencer, John Campbell, Willem Kuyken

**Affiliations:** a Mood Disorders Centre, University of Exeter, Exeter, EX4 4QQ, UK; b Population Health Sciences, University of Bristol, Canynge Hall, Bristol, BS8 2PN, UK; c School of Psychology, Cardiff University, 70 Park Place, Cardiff, CF10 3AT, UK; d Centre for the Psychology of Learning and Experimental Psychopathology, KU Leuven, Tiensetraat 102, Box 3712, 3000, Leuven, Belgium; e Université libre de Bruxelles, Avenue Franklin Roosevelt 50, 1050, Bruxelles, Belgium; f Department of Health Sciences (MHARG), University of York, York, Y010 5DD, UK; g Clinical Psychological Science, Maastricht University, PO Box 616, 6200 MD, Maastricht, the Netherlands; h University of Exeter Medical School, St Luke's Campus, Heavitree Road, Exeter, EX1 2LU, UK; i Department of Psychiatry, University of Oxford, Warneford Hospital Oxford, OX3 7JX, UK

**Keywords:** Depression anhedonia, Wellbeing, CBT, Psychotherapy, RCT

## Abstract

**Background:**

Anhedonia (reduced interest/pleasure) symptoms and wellbeing deficits are core to depression and predict a poor prognosis. Current depression psychotherapies fail to target these features adequately, contributing to sub-optimal outcomes. Augmented Depression Therapy (ADepT) has been developed to target anhedonia and wellbeing. We aimed to establish clinical and economic proof of concept for ADepT and to examine feasibility of a future definitive trial comparing ADepT to Cognitive Behavioural Therapy (CBT).

**Methods:**

In this single-centre, open-label, parallel-group, pilot randomised controlled trial, adults meeting diagnostic criteria for a current major depressive episode, scoring ≥10 on the Patient Health Questionnaire (PHQ-9) and exhibiting anhedonic features (PHQ-9 item 1 ≥ 2) were recruited primarily from high intensity Improving Access to Psychological Therapy (IAPT) service waiting lists in Devon, UK. Participants were randomised to receive 20 sessions of CBT or ADepT, using a mimimisation algorithm to balance depression severity and antidepressant use between groups. Treatment was delivered in an out-patient university-based specialist mood disorder clinic. Researcher-blinded assessments were completed at intake and six, 12, and 18 months. Co-primary outcomes were depression (PHQ-9) and wellbeing (Warwick Edinburgh Mental Wellbeing Scale) at 6 months. Primary clinical proof-of-concept analyses were intention to treat. Feasibility (including safety) and health economic analyses used complete case data. This trial is registered at the ISRCTN registry, ISRCTN85278228.

**Findings:**

Between 3/29/2017 and 7/31/2018, 82 individuals were recruited (102% of target sample) and 41 individuals were allocated to each arm. A minimum adequate treatment dose was completed by 36/41 (88%) of CBT and 35/41 (85%) of ADepT participants. There were two serious adverse events in each arm (primarily suicide attempts; none of which were judged to be trial- or treatment-related), with no other evidence of harms. Intake and six-month primary outcome data was available for 37/41 (90%) CBT participants and 32/41 (78%) ADepT participants. Between-group effects favoured ADepT over CBT for depression (meanΔ = −1.35, 95% CI = −3.70, 1.00, d = 0.23) and wellbeing (meanΔ = 2.64, 95% CI = −1.71, 6.99, d = 0.27). At 18 months, the advantage of ADepT over CBT was preserved and ADepT had a >80% probability of cost-effectiveness.

**Interpretation:**

These findings provide proof of concept for ADepT and warrant continuation to definitive trial.

**Funding:**

NIHR Career Development Fellowship.

**Research in context**

**Evidence before this study**

Systematic reviews of depression clinical trials reveal that outcomes following psychotherapies are not optimised (depression response rates of <50%; diagnostic remission rates of <65%; >50% relapse rates within two years in those who do remit; the presence of residual wellbeing deficits after treatment). Narrative reviews identify that the two cardinal symptoms of depression are depressed mood and anhedonia, reflecting disturbances of the negative valence system (NVS) and the positive valence system (PVS) respectively. Individuals with depression report that repairing both systems is key to recovery and epidemiological data indicate both are prognostically important. Secondary analyses of clinical trials demonstrate that current mainstream therapies like Cognitive Behavioural Therapy (CBT) adequately repair the NVS but not the PVS, potentially contributing to sub-optimal treatment outcomes. A scoping review was conducted in May 2023 in PubMed to identify evaluations of psychological interventions targeting anhedonia in depression (including the terms “anhedonia”, “depression”, and “randomised controlled trials”). This identified an emerging class of transdiagnostic psychotherapies that target the PVS. However, none of these psychotherapies have a joint focus on the PVS and NVS nor have been designed specifically to target anhedonic depression.

**Added value of this study**

The present pilot study is the first randomised controlled trial that evaluates Augmented Depression Therapy (ADepT)—a novel wellbeing-oriented treatment that targets both NVS and PVS dysfunction—relative to CBT in the treatment of anhedonic depression. The findings show that ADepT leads to large and clinically meaningful improvements in depression, wellbeing, anhedonia, anxiety, and broader measures of NVS and PVS function. ADepT is not inferior, and is potentially superior, to CBT in building wellbeing and reducing depression and anhedonia immediately after acute treatment and at longer term follow-up. ADepT also showed potential to be cost-effective, dominating CBT from a health economic perspective.

**Implications of all the available evidence**

The present data, in combination with trials evaluating other transdiagnostic interventions targeting the PVS, show that it is possible to repair anhedonia and wellbeing deficits more effectively in clinical populations. Furthermore, the present findings suggest that ADepT has potential to lead to clinically meaningful improvements in wellbeing and depression outcomes compared to current mainstream treatments for anhedonic depression like CBT. A larger, suitably powered definitive trial is indicated to assess definitively the clinical- and cost-effectiveness of ADepT relative to CBT.

## Introduction

Depression is widespread (lifetime prevalence of 20%), disabling, chronically recurrent, a significant contributor to global disability, and results in extensive social and economic costs.^1^, ^2^, ^3^ Change during current psychotherapeutic and drug treatment is clinically significant but sub-optimal (50% of patients remit, 50% of whom relapse within two-years, meaning a sustained recovery rate of around 25%^4^^,^^5^). There have been no stepwise advances in depression treatment outcomes for forty years since Beck's seminal work developing Cognitive Behavioural Therapy (CBT)^6^ and there is a pressing need for innovation of clinically effective and value for money interventions that can be implemented in routine care.

A paradigm shift in treatment of depression may be required. The two core symptoms of depression are pervasive depressive mood and anhedonia (reduced interest/pleasure). These symptoms emerge from disruptions to dissociable neurobiological systems.^7^ Over-activation of the negative valence system (NVS) increases negative affect (NA), while under-activation of the positive valence system (PVS) inhibits positive affect (PA). Existing depression psychotherapies like CBT and Behavioural Activation (BA) effectively repair negative affect/depressed mood, but not positive affect/anhedonia, meaning individuals experience residual PVS dysfunction after treatment.^8^^,^^9^ Given that higher levels of positive affect predict increased likelihood of remission and reduced risk of relapse, a failure to repair PVS disturbances is likely contributing to sub-optimal outcomes.^10^ Furthermore, existing treatments view depression as an acute, curable illness. However, depression often follows a recurrent, relapsing course so is better conceptualised as a chronic vulnerability that individuals need to learn to live well ‘alongside’ (a recovery focus). Service-user definitions of recovery prioritise restoring wellbeing as well as symptom relief.^11^ Wellbeing treatment gains often lag behind symptom relief.^12^

Augmented Depression Therapy (ADepT) has been developed to target both NVS and PVS dysfunction and aims both to enhance wellbeing and reduce depression symptoms over the longer term.^13^ ADepT is an individual treatment (consisting of 15 acute and five booster sessions) that is a solution-focused, cognitively augmented, behavioural activation approach. ADepT involves identifying client values; behaviourally activating clients to work towards values consistent goals; and overcoming barriers to being resilient (managing challenges to reduce NA) and thriving (taking opportunities to maximize PA). Building wellbeing (capacity to experience pleasure, meaning and social connection in life) and functional recovery is the primary focus, with depression conceptualised as patterns of thinking, feeling and behaving that serve as barriers to achieving this goal. ADepT has some similarities to an emerging class of depression and transdiagnostic interventions targeting anhedonia and the PVS.^14^ However, ADepT is unique in that it has been designed specifically with anhedonic depression in mind, focuses equally on disturbances of the NVS and the PVS, and sets enhancing wellbeing as the primary treatment emphasis. ADepT is of a similar dose to standard care (CBT/BA) and is therefore comparable in delivery cost. To enhance implementation, ADepT has been designed so that existing CBT/BA therapists will be able to deliver it with minimal additional training. In a UK context, the target workforce are high intensity therapists working in NHS talking therapy services for anxiety and depression (NTTad; previously known as Improving Access Psychological Therapy [IAPT] services).

A multiple randomised baseline case series preliminarily examined feasibility, acceptability and clinical efficacy of ADepT in thirteen individuals with depression primarily recruited from NTTad high intensity waiting lists.^13^ ADepT was acceptable to individuals with depression and therapists. In addition, effect size improvements in depression, anhedonia and wellbeing were large and clinically meaningful after acute treatment, were largely sustained over one year follow-up, and there was no evidence of harms.

A randomised controlled trial (RCT) powered to detect minimum clinically important difference (MCID) on wellbeing and depression outcomes is now needed to evaluate the clinical- and cost-effectiveness of ADepT relative to existing therapies like CBT. Before proceeding to a large-scale definitive RCT of this kind, it is prudent to optimise further the ADepT protocol, confirm there are no harms resulting from ADepT, resolve any uncertainties about the planned RCT design, and establish further proof of concept about the possible clinical- and cost-effectiveness of ADepT. A pilot RCT—a smaller scale ‘mock up’ of the planned subsequent definitive trial—is an efficient way to achieve these aims. A pilot RCT comparing ADepT to CBT in the treatment of depression with a focus on both clinical- and cost-effectiveness was therefore conducted.^15^ Pre-specified feasibility continuation rules to proceed to definitive trial were: >60% of target sample was recruited; primary outcome data were collected on >60% of individuals at all follow-ups; >60% of clients in each arm completed a minimum adequate treatment dose (>50% acute sessions); no unexpected and clearly trial- or treatment-related serious adverse events occurred; and any remaining concerns about the intervention or trial design could be rectified. In addition, it was assessed if there was sufficient proof of signal regarding the potential clinical- and cost-effectiveness of ADepT to warrant continuation to definitive trial.

## Methods

### Study design and participants

A single-site pilot study was hosted at the Accessing Evidence Based Psychological Therapies (AccEPT) clinic, a National Health Service (NHS) commissioned outpatient psychological therapies service for mood disorders in Devon, UK. The trial aimed to recruit 80 currently clinically depressed participants (40 per arm), with an expected retention rate of 80% (64 in total; 32 per arm). This target sample size was chosen to have sufficient precision to estimate recruitment, retention and sustained recovery rates with a margin of error of <15% and to be sufficient to detect medium effect-size within-arm changes.^15^ No adjustment was made to take into account potential therapist clustering effects.

Inclusion criteria were: being aged over 18 years; meeting diagnostic criteria for a current major depressive episode according to the Structured Clinical Interview for Diagnosis (SCID-I^16^); scoring in the clinical range on the Patient Health Questionnaire-9 (PHQ-9^17^); describing depression as the primary presenting problem; and having adequate English to make use of therapy and complete research assessments without need of a translator. Given the focus on PVS disturbances in ADepT, individuals were selected to show marked anhedonic features (item one of PHQ-9 measuring anhedonia ≥2; more than half the days or nearly every day). Exclusion criteria were: presence of psychosis, learning disability, and/or organic brain change; substance abuse that compromised ability to use therapy; marked risk of self-harm or suicide that could not be safely managed in an outpatient clinic setting; and currently receiving any other psychosocial therapy. This reflects standard exclusion criteria used in NTTad settings in the UK NHS, where any subsequent definitive trial would be run. The primary method of recruitment was via local high-intensity NTTad service waiting lists in Devon, supplemented via self-referral and clinician-referral from other local NHS services.

Clinical and health economic assessments were conducted (face-to-face or via telephone) at intake and at 6-, 12-, and 18-month follow-up (6 months as the primary clinical outcome point; 18 months as the primary health economic outcome point). Trial recruitment ran from 3/29/2017 to 7/31/2018, with the final assessment completed by 1/1/2020 (prior to the COVID-19 pandemic). The trial received approval from the UK National Research Ethics Service (REC reference: 17/SW/0009) and the Health Research Authority (IRAS ID: 216,871) and was overseen by an independent combined trial steering committee and data monitoring and ethics committee (TSC/DMEC). All participants gave written, informed consent and received a £10 honorarium and had their travel costs reimbursed for each research assessment they completed. Experts by lived experience contributed to intervention design and project governance (SOM Section 1). This trial is reported according to the CONSORT 2010 guidance statement for pilot or feasibility trials, the CONSORT 2017 guidance statement for reporting nonpharmacological trials, the CONSORT 2022 guidance statement for reporting harms, and the Consolidated Health Economic Evaluation Reporting Standards (CHEERS) 2022 guidance statement.

### Randomisation and masking

Participants were randomly allocated (in a 1:1 ratio) to ADepT or CBT after intake assessment, the first 24 on a truly random basis and the remainder via a minimization method to maximize chances of balance between arms on stratification variables of depression severity (PHQ-9 < 19 or ≥ 19) and anti-depressant usage (taking or not taking anti-depressants). To ensure concealment, allocation was undertaken by a password-protected bespoke website maintained by the Peninsula Clinical Trials Unit, independent of the trial. The trial administrator accessed the website and informed participants of their allocation. Given the nature of the interventions, treatment was open label (patients and clinicians were aware of treatment allocation). However, outcome assessors were masked to participants’ allocations (and assessments were scheduled independent of treatment sessions).

### Procedures

Therapies were delivered in individual weekly (mostly face-to-face) format, with each session lasting 60 min. CBT consisted of up to 20 sessions, following the Beckian CBT protocol used in the COBRA depression trial.^18^ This focused on engaging the client with pleasant activities and then identifying and altering patterns of negative thinking that maintain low mood, with the client engaging with home learning between sessions. Therapist style predominantly focused on identifying areas of difficulty and problem-solving with the client how to overcome these.

ADepT consisted of up to 15 acute sessions and up to 5 (flexibly scheduled) booster sessions over the following year to help clients sustain gains and minimise the risk of relapse. Treatment focused on supporting clients to clarify values; work towards values-consistent goals in vocational, recreational, relational and self-care domains; and to learn to act opposite to depressogenic mechanisms that inhibit the capacity to thrive during opportunities (targeting the PVS) and to be resilient during challenges (targeting the NVS) while doing so. Therapists adopted a positive, future-focused, solution-focused and reinforcing style that helped clients develop the capacity to notice and utilize strengths. The end phase of therapy developed a wellbeing plan to continue progress over the coming months and booster sessions reviewed and troubleshot progress with this plan (see SOM Section 2 for a detailed intervention description).

Therapists (six CBT, four ADepT) were experienced CBT practitioners (either NTTad high intensity therapists, clinical psychologists, or nurse therapists with on average approximately twenty years’ experience of working in mental health settings and having practiced CBT for at least ten years). There was no overlap in therapists between arms. Therapists completed a one-day group training session prior to starting the trial and ongoing therapist supervision was provided in a small group format (up to 90 min weekly per therapist as required).

At the end of treatment, participants rated if treatment was acceptable and satisfactory and if they would recommend treatments to others on five-point Likert scales. Treatment sessions were recorded and a random subset of tapes (four for each CBT therapist, six for each ADepT therapist) were assessed for competence, using the Cognitive Therapy Rating Scale-Revised^19^ for CBT sessions and a bespoke scale for ADepT sessions. Tapes were also rated as to the degree to which they showed fidelity to the appropriate treatment protocol and differentiated between ADepT and CBT.

### Outcomes

The co-primary self-report outcomes were the nine-item PHQ-9^17^ and the 14-item Warwick Edinburgh Mental Wellbeing Scale (WEMWBS^20^). The PHQ-9 measures depression severity (scores ≥10 indicating caseness; individual MCID improvement = 1.7 point decrease). It aligns with DSM-V criteria for depression, includes an item measuring anhedonia, is part of the core measurement battery routinely used in IAPT settings, and has been used as the primary outcome in recent depression therapy trials.^18^ The WEMWBS measures wellbeing (UK general population score 51.61 [SD = 8.71]; scores <43 indicating languishing wellbeing and MCID improvement defined as >2.8 point increase, permission to use needed from scale developers). Both measures are widely used, free, and have been translated into multiple languages.

Secondary self-report outcomes were the Generalized Anxiety Disorder Scale (GAD-7^21^) to index anxiety; the Snaith Hamilton Pleasure Scale (continuous scoring form; SHAPS^22^) to index anhedonia; the Positive and Negative Affect Schedule (past week form; PANAS^23^) to index positive and negative affect; the Mood and Anxiety Symptom Questionnaire Short-Form (MASQ-S30^24^) to index anhedonia, general distress and anxious arousal; the IAPT phobia scale^25^ to assess phobia symptoms; and the Work and Social Adjustment Scale (WSAS^26^) to assess functional impairment. Interview measures were the Structured Interview Guide for the Hamilton Depression Rating Scale (SIGH-D; 17-item version^27^) to index depression severity; the Structured Interview Guide for the Hamilton Anxiety Rating Scale (SIGH-A^28^) to index anxiety severity; the SCID-I^16^ to assess current depression diagnostic status; and the Longitudinal Interval Follow-Up Evaluation (LIFE^29^) to index depression diagnostic status over the previous six-month period.

For health economic evaluation, intervention usage was measured from clinical records and information about training, supervision and other non-face-to-face activities was taken from therapists and trainers. Use of broader health and care services was measured using a modified version of the Adult Service Use Schedule (AD-SUS^30^). Health related quality of life was measured using the EuroQoL-5D-5L measure of health status (EQ-5D-5L^31^) and wellbeing related quality of life was measured using the ICEpop Capability Measurement for Adults (ICECAP^32^). Qualitative and quantitative process evaluation outcomes were also collected as specified in the trial protocol paper^15^; results of these analyses will be reported elsewhere.

### Statistical analysis

The analysis was conducted by the trial principal investigator (BD). Feasibility and clinical proof-of-concept analyses broadly followed the pre-specified plan in the protocol^15^ with some minor adaptations and post hoc additions, whereas the cost-effectiveness analyses were entirely post hoc (see SOM Section 3c). The trial continuation rules were evaluated via descriptive reporting of rates of recruitment, data retention, treatment engagement, adverse events, and other trial related procedures. Proof of concept analyses were run an intent-to-treat basis using multiple imputation to simulate missing data, except where otherwise stated. As this is a pilot trial with a small sample size, raw score and effect size (Cohen's d) 95% confidence intervals (CIs) rather than statistical significance are reported in all analyses. Effect sizes were interpreted according to Cohen's rules of thumb (d: >0.2 = small; >0.5 = medium; >0.8 = large effects). Paired sample t-test analyses examined change from intake to 6 months on continuous outcome variables in each arm. Linear regressions estimated between-group differences on continuous outcomes, each covarying for trial stratification variables and intake levels of the dependent variable. It was examined whether lower bound CIs crossed into the range where CBT might be MCID superior to ADepT and whether upper bound CIs crossed into the range where ADepT might be MCID superior to CBT. Where MCID values had been established for outcomes (PHQ-9, WEMWBS, GAD-7, and HDRS), these were used to set these MCID thresholds. Where MCID values were not available, these MCID thresholds were set as a standardized effect size difference (Cohen's d) of ± 0.24.^33^ Secondary analyses of area-under-the-curve (AUC) across all follow-ups assessed longer term continuous clinical outcomes, using comparable linear regressions and interpreting standardized effect size 95% CIs. The proportion of individuals remitting (falling in non-clinical ranges) and/or responding (showing a 50% improvement in symptoms) on key outcomes at 6 months was computed. Between-group effects on binary outcomes were estimated using logistic regressions, covarying for trial stratification variables, and odds ratio 95% CIs were interpreted.

In the subgroup of individuals who had remitted at the 6-month assessment, rates of subsequent depressive relapse were examined until the end of the 18-month follow-up. Between group-effects were estimated by interpreting the hazard ratio from a Cox regression proportional hazard model (covarying for the trial stratification variables), taking into account missing follow-up data via censoring rather than multiple imputation.

We benchmarked complete case ADepT and CBT six-month outcomes to those observed in the COBRA^18^ and CoBalT^34^ psychotherapy trials conducted in comparable UK primary care depressed populations and to meta-analytic findings.^4^ We refitted six-month complete case linear models on co-primary PHQ-9 and WEMWBS outcomes using a Bayesian approach, plotting the probability of the difference in outcomes if new clients were treated with ADepT rather than CBT.

Post hoc health economic analyses took a UK NHS and personal social services perspective and adopted an 18-month (within-trial) time horizon. EQ-5D and ICECAP measures were converted into utility scores at each assessment. AUC methods were used to compute quality adjusted life years (QALYs) across the eighteen-month trial follow-up. Intervention costs and broader health care utilization costs measured on the AD-SUS were summed across the follow-up assessments. Costs and QALYs used a 3.5% annual discounting rate. Linear regression analyses were run to estimate between-group differences for costs and QALYs, each covarying for trial stratification variables, intake EQ-5D and ICECAP utility scores, and (log transformed) resource utilization in the 6 months prior to the trial. Separate cost-effectiveness (utility) analyses were run for EQ-5D and ICECAP data. In each case, non-parametric bootstrapping (with 1000 resamples) was used to generate a distribution of mean costs and QALYs to capture sampling uncertainty. Incremental cost-effectiveness ratios (ICERs), cost-effectiveness planes, cost effectiveness acceptability curves (CEACs), and (net) expected incremental benefit (EIB) plots were generated from these bootstrapped distributions to estimate the probability that ADepT is cost-effective compared to CBT as a function of different values a decision-maker is willing to pay per QALY. The default willingness to pay threshold was set at £20,000 to align with UK National Institute of Clinical Excellence (NICE) guidance. See SOM Section 3 for a detailed analysis protocol.

### Role of the funding source

The funder of the study had no role in study design, data collection, data analysis, data interpretation, or writing of the report. BD and LW had access to the dataset and had final responsibility for the decision to submit for publication.

## Results

### Feasibility

**Fig. 1:**
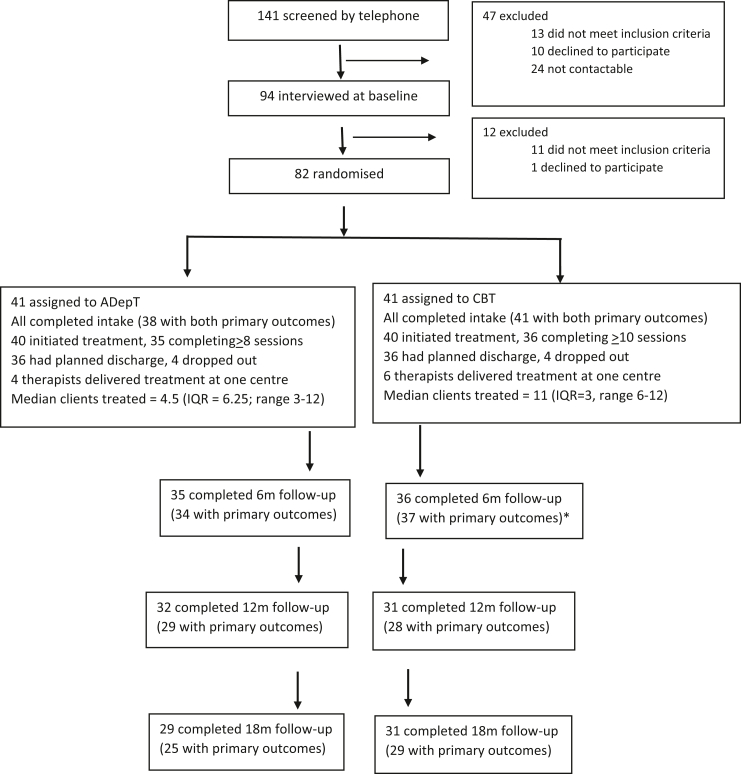
ADepT Pilot Trial Consort Diagram. Note: CBT = Cognitive behavioural therapy; ADepT = Augmented depression therapy; IQR = Inter-quartile range; ∗ = one participant in CBT did not attend 6m assessment but did post back outcome measures when requested.

Eighty-two participants were recruited (103% of planned sample; >60% target; Fig. 1). Groups were well balanced in terms of clinical and sociodemographic characteristics (Table 1) and the sample was representative of the intended target population (predominantly moderate to severe depression with marked anhedonia, comorbid anxiety and languishing levels of wellbeing, and who had not responded to previous low intensity therapy or anti-depressant medication in the current depressive episode).

**Table 1:** Intake demographic and clinical characteristics of participants and therapists.

	CBT	ADepT
Age (years)	36.00 (23.00)	41.00 (21.00)
Gender (female)	29/41 (71%)	22/41 (54%)
Gender (male)	12/41 (29%)	19/41 (46%)
Ethnicity (White British)	38/41 (93%)	35/41 (85%)
Ethnicity (White other)	3/41 (7%)	5/41 (12%)
Ethnicity (Mixed other)	0/41 (0%)	1/41 (2%)
Relationship Status (in relationship)^a^	22/41 (54%)	19/41 (46%)
Employment Status (employed)^b^	29/37 (78%)	26/38 (68%)
Taking anti-depressants	27/41 (66%)	27/41 (66%)
Previous psychological treatment	34/38 (89%)	32/40 (80%)
Previously attempted suicide	9/41 (22%)	7/41 (17%)
Age of first onset of depression	18.00 (7.00)	18.50 (17.00)
First onset pre adulthood	21/41 (51%)	20/41 (49%)
≥3 prior depressive episodes	27/32 (84%)	23/26 (88%)
PHQ-9 depression severe status (≥19)	21/41 (51%)	19/41 (46%)
WEMWBS languishing status (<43)	41/41 (100%)	37/38 (97%)
WSAS severe functional impairment (≥20)	35/41 (85%)	32/39 (82%)
GAD-7 anxiety caseness (≥8)	35/41 (85%)	34/39 (87%)
HDRS depression caseness (≥8)	41/41 (100%)	40/40 (100%)
HARS anxiety at least moderate severity (≥18)	38/41 (93%)	39/41 (95%)
SHAPS anhedonia caseness (≥24)	38/40 (95%)	35/38 (92%)
Number of therapists	6	4
Gender (female) of therapists	5/6 (83%)	2/4 (50%)
Age (years) of therapists	46.50 (15.00)	49.50 (7.00)
Clients treated by each therapist	4.50 (6.25)	11.00 (3.00)

Note: Table reports complete case data; continuous variables are median (interquartile range) values; categorical variables are count (%). CBT = Cognitive behavioural therapy; ADepT = Augmented depression therapy; PHQ-9 = Patient health questionnaire; WEMWBS = Warwick edinburgh mental wellbeing scale; WSAS = Work and social adjustment scale; GAD-7 = Generalized anxiety disorder scale; HDRS = Hamilton depression rating scale; HARS = Hamilton anxiety rating scale; SHAPS = Snaith Hamilton pleasure scale; where group size is less than 41, this reflects missing data or participants choosing not to answer the question.

a Relationship defined as co-habiting, married or civil partnership.

b Employment defined as paid or voluntary employment or being in formal study in past six months, taken from AD-SUS interview.

There were acceptable rates of assessment attendance (82/82 [100%], 71/82 [87%], 63/82 [77%], and 60/82 [73%] of sample randomised at intake and 6-,12-, and 18-months respectively); co-primary outcome data completeness (79/82 [96%], 71/82 [87%], 57/82 [70%] and 54/82 [66%] of sample randomised at intake and 6-,12-, and 18-months respectively; >60% target); and secondary outcome data completeness (SOM Tables S5 and S6). A minimum adequate dose of treatment (>50% of acute sessions) was completed by 36/41 (88%) of CBT and 35/41 (85%) of ADepT participants (>60% target). A majority of participants attended close to the full acute treatment dose (CBT acute sessions attended [out of 20]: mode = 20, mean = 16.66, SD = 5.58; ADepT acute sessions attended [out of 15]: mode = 15, mean = 12.95, SD = 4.33) and ADepT participants engaged satisfactorily with booster sessions (sessions attended [out of 5]: mode = 5, mean = 3.07, SD = 2.21). Total number of sessions attended across acute and booster phases of ADepT (mode = 20, mean = 16.02, SD = 6.04) was broadly comparable to CBT session attendance.

There were two serious adverse events in each arm (two non-fatal overdoses in the CBT arm; one non-fatal overdose and one planned minor surgical procedure in the ADepT arm). None of these events were judged as trial- or treatment-related by the TSC/DMEC (SOM Table S7). No participants showed reliable deterioration on the PHQ-9, WEMWBS or GAD-7 at six months. Of those clients who completed post-treatment experience ratings (34/41 [83%] in CBT; 30/41 [73%] in ADepT), all clients rated treatment as at least moderately acceptable and were at least moderately satisfied with it; 31/34 (91%) of CBT participants and 29/31 (94%) of ADepT participants would recommend treatment they received to others (SOM Table S8). Tape ratings indicated that overall competence, fidelity and differentiation criteria were met by all therapists (SOM Table S9). Participants rated the PHQ-9 and WEMWBS as being credible outcome measures (SOM Table S10). All other trial procedures functioned acceptably, and all pre-specified feasibility continuation criteria were met (see SOM Section 3 for detailed feasibility analyses).

### Clinical proof of concept

Table 2 reports continuous outcomes at each assessment, AUC across all assessments, and scale internal reliabilities at intake assessment. Internal reliability was adequate for all measures (α′s > 0.60). There were large effect size improvements at 6 months in both the ADepT and CBT arms on the PHQ-9 and WEMWBS co-primary outcomes and a majority of secondary outcomes (SOM Table S11). The exceptions were medium effect size for the IAPT phobia scale in both arms and in the CBT arm only for MASQ-S30 arousal and WSAS functional impairment.

**Table 2:** Continuous outcome measures at each assessment point in the CBT and ADepT arms.

	Arm	Intake	6m	12m	18m	18m AUC
*PHQ*-*9 depression*	CBT	18.10 (3.74)	9.89 (6.22)	9.48 (5.89)	9.97 (6.77)	16.88 (7.30)
α = 0.66	ADepT	18.22 (4.27)	8.49 (5.53)	8.45 (5.97)	8.84 (7.05)	14.74 (6.85)
*WEMWBS wellbeing*	CBT	29.34 (5.67)	40.95 (9.60)	41.36 (9.73)	38.52 (10.61)	57.64 (10.58)
α = 0.81	ADepT	30.43 (6.36)	43.97 (10.07)	43.05 (11.47)	43.20 (13.58)	62.32 (14.22)
GAD-7 anxiety	CBT	12.54 (4.72)	7.32 (5.00)	7.17 (5.00)	7.97 (5.46)	12.51 (6.56)
α = 0.78	ADepT	12.97 (4.42)	5.57 (4.05)	6.66 (5.52)	7.12 (5.90)	11.27 (6.32)
SHAPS anhedonia	CBT	34.58 (5.25)	27.78 (6.60)	26.83 (7.19)	27.40 (7.22)	42.91 (7.56)
α = 0.82	ADepT	34.24 (6.97)	24.44 (7.13)	25.30 (8.02)	25.76 (10.56)	39.27 (10.74)
PANAS positive affect	CBT	17.98 (5.72)	27.51 (8.23)	25.32 (9.57)	26.91 (8.61)	36.87 (9.47)
α = 0.85	ADepT	17.54 (5.33)	30.03 (9.02)	26.65 (8.98)	31.21 (9.72)	40.78 (11.88)
PANAS negative affect	CBT	31.98 (8.73)	22.05 (8.14)	21.93 (7.08)	24.74 (10.32)	36.16 (10.65)
α = 0.86	ADepT	29.34 (7.91)	19.12 (6.93)	19.96 (8.59)	19.71 (9.28)	31.19 (10.02)
MASQ anhedonia	CBT	45.14 (4.30)	36.74 (8.50)	36.24 (9.16)	37.97 (9.50)	43.08 (9.58)
α = 0.82	ADepT	45.39 (4.67)	33.31 (9.48)	34.89 (9.07)	34.56 (10.72)	38.75 (12.40)
MASQ general distress	CBT	35.15 (6.64)	26.08 (9.25)	25.00 (7.87)	26.43 (9.65)	40.86 (10.53)
α = 0.77	ADepT	35.12 (7.63)	21.58 (7.58)	23.11 (8.86)	25.16 (11.13)	36.36 (10.90)
MASQ anxious arousal	CBT	22.73 (8.14)	17.59 (6.56)	16.05 (5.08)	17.00 (6.46)	26.85 (7.71)
α = 0.83	ADepT	22.26 (7.91)	16.70 (7.16)	18.29 (7.35)	19.12 (8.42)	28.12 (10.44)
WSAS functioning	CBT	24.40 (6.72)	16.58 (9.59)	15.10 (8.99)	18.93 (11.11)	26.90 (11.11)
α = 0.76	ADepT	25.85 (7.94)	16.03 (9.11)	13.84 (9.79)	14.25 (11.51)	24.52 (12.10)
IAPT phobia scale	CBT	7.90 (5.67)	5.24 (5.11)	6.17 (5.35)	6.96 (6.85)	9.72 (7.35)
α = 0.79	ADepT	8.77 (5.72)	5.26 (5.02)	5.57 (5.79)	4.75 (5.88)	9.24 (7.98)
HDRS depression	CBT	19.05 (4.18)	9.07 (5.81)	9.00 (7.25)	10.27 (7.89)	16.52 (7.41)
α = 0.60	ADepT	18.13 (3.91)	7.25 (5.17)	7.65 (5.89)	8.07 (8.40)	11.46 (6.91)
HARS anxiety	CBT	15.37 (6.12)	7.89 (5.51)	7.32 (5.64)	9.63 (6.71)	13.70 (7.23)
α = 0.64	ADepT	15.22 (5.62)	6.34 (5.48)	8.09 (6.74)	8.69 (8.10)	13.29 (8.27)

Note: Table reports complete case data; primary outcomes italicized; data are mean (standard deviation) values; α = scale internal reliabilities at intake assessment. AUC = Area under the curve; CBT = Cognitive behavioural therapy; ADepT = Augmented depression therapy; PHQ-9 = Patient health questionnaire; WEMWBS = Warwick edinburgh mental wellbeing scale; GAD-7 = Generalized anxiety disorder scale; SHAPS=Snaith hamilton pleasure scale; PANAS = Positive and negative affect schedule; MASQ = Mood and anxiety symptom questionnaire-short form; WSAS = Work and social adjustment scale; IAPT = Improving access to psychological therapies; HDRS = Hamilton depression rating scale; HARS = Hamilton anxiety rating scale.

Table 3 reports between-group difference estimates (in raw score and standardized effect size units) from 6-month analyses. When considering raw score estimates (PHQ-9, WEMWBS, GAD-7, and HDRS), upper bound 95% CIs crossed into the range where ADepT may be MCID superior to CBT and lower bound 95% CIs did not cross into the range where CBT may be MCID superior to ADepT. When considering standardized effect size difference estimates, upper bound 95% CIs crossed into the range where ADepT may be MCID superior to CBT (ds > 0.24) and lower bound 95% CIs did not cross into the range where CBT may be MCID superior to ADepT (ds ≥−0.24) for the co-primary depression and wellbeing outcomes and nearly all secondary outcome variables. The exceptions were that 95% CIs crossed into ranges where ADepT could be either MCID superior to CBT or vice versa for WSAS functional impairment and the IAPT phobia scale. 95% CIs did not cross zero for SHAPS anhedonia and MASQ-S30 general distress, meeting conventional frequentist criteria for ADepT being superior to CBT.

**Table 3:** Summary of between group six-month and eighteen-month cumulative analyses.

	Sample	Raw Δ (95% CI)	d Δ (95% CI)
**Six-months**			
*PHQ*-*9 depression*	37/35	−1.35 (−3.70, 1.00)	0.23 (−0.17, 0.63)
*WEMWBS wellbeing*	37/32	2.64 (−1.71, 6.99)	0.27 (−0.17, 0.71)
GAD-7 anxiety	37/34	−1.72 (−3.53, 0.10)	0.37 (−0.02, 0.76)
SHAPS anhedonia	36/32	−3.05 (−6.02, −0.07)	0.44 (0.01, 0.86)
PANAS positive affect	37/32	2.74 (−1.09, 6.59)	0.32 (−0.13, 0.76)
PANAS negative affect	37/32	−2.39 (−5.46, 0.68)	0.31 (−0.09, 0.71)
MASQ-S30 anhedonia	36/32	−3.46 (−7.36, 0.44)	0.38 (−0.05, 0.81)
MASQ-S30 general distress	36/32	−4.27 (−7.79, −0.74)	0.49 (0.08, 0.89)
MASQ-S30 anxious arousal	36/32	−1.86 (−4.24, 0.52)	0.27 (−0.08, 0.62)
WSAS functional impairment	36/34	−1.08 (−5.10, 2.94)	0.12 (−0.32, 0.55)
IAPT phobia scale	37/34	−0.01 (−1.90, 1.88)	0.00 (−0.37, 0.38)
HDRS depression	36/34	−1.44 (−3.87, 1.00)	0.26 (−0.18, 0.70)
HARS anxiety	36/35	−1.44 (−3.92, 1.04)	0.26 (−0.19, 0.71)
**Eighteen-months (AUC)**			
*PHQ*-*9 depression*	33/32	−2.05 (−4.94, 0.85)	0.29 (−0.12, 0.69)
*WEMWBS wellbeing*	33/30	4.32 (−1.52, 10.15)	0.34 (−0.12, 0.81)
GAD-7 anxiety	33/31	−1.86 (−4.57, 0.86)	0.29 (−0.13, 0.71)
SHAPS anhedonia	32/30	−3.59 (−7.47, 0.29)	0.38 (−0.03, 0.80)
PANAS positive affect	32/29	4.05 (−0.81, 8.91)	0.38 (−0.08, 0.83)
PANAS negative affect	32/29	−4.26 (−8.41, −0.11)	0.40 (0.01, 0.80)
MASQ-S30 anhedonia	32/29	−4.74 (−9.44, −0.05)	0.43 (0.00, 0.85)
MASQ-S30 general distress	32/29	−4.47 (−8.53, −0.40)	0.41 (0.04, 0.79)
MASQ-S30 anxious arousal	32/29	−0.05 (−3.30, 3.19)	0.01 (−0.35, 0.36)
WSAS functional impairment	33/31	−3.67 (−8.23, 0.89)	0.32 (−0.08, 0.71)
IAPT phobia scale	33/30	−1.16 (−3.75, 1.44)	0.15 (−0.19, 0.49)
HDRS depression	33/32	−1.91 (−4.88, 1.06)	0.26 (−0.15, 0.67)
HARS anxiety	33/33	−0.87 (−3.88, 2.14)	0.11 (−0.28, 0.50)

Note: CBT = Cognitive behavioural therapy; ADepT = Augmented depression therapy; PHQ-9 = Patient health questionnaire; WEMWBS = Warwick edinburgh mental wellbeing scale; GAD-7 = Generalized anxiety disorder scale; SHAPS = Snaith hamilton pleasure scale; PANAS = Positive and negative affect schedule; MASQ-S30 = Mood and anxiety symptom questionnaire-short form; WSAS = Work and social adjustment scale; IAPT = Improving access to psychological therapies; HDRS = Hamilton depression rating scale; HARS = Hamilton anxiety rating scale. Sample = number of participants with complete data in CBT/ADepT arm; AUC = area under the curve across assessments; RawΔ = estimated raw score differences between groups (ADepT—CBT) from intent-to-treat linear regressions (using multiple imputation to simulate missing values); dΔ = estimated standardized (Cohen's d) difference between groups (ADepT—CBT) from intent-to-treat linear regressions (using multiple imputation to simulate missing values); minimum clinically important difference (MCID) estimates for PHQ-9 = 1.70 points, for WEMWBS = 2.80 points, for GAD-7 = 1.15 points, for HDRS = 3.00 points; there are no established estimates for other scales.

Table 3 also reports between-group effect size estimates for 18-month AUC analyses. For the co-primary depression and wellbeing outcomes and most secondary outcomes, upper bound 95% CIs crossed into the range where ADepT may be MCID superior to CBT (ds >0.24) and lower bound 95% CIs did not cross into the range where CBT may be MCID superior to ADepT (ds ≥ −0.24). The exceptions were that 95% CIs crossed into ranges where ADepT could be either MCID superior to CBT or vice versa for MASQ-S30 arousal and HARS anxiety. 95% CIs did not cross zero for PANAS negative affect and MASQ-S30 anhedonia and distress, meeting conventional frequentist criteria for superiority of ADepT over CBT.

Table 4 summarises binary outcomes in each group and the estimates of between-group effects from logistic regressions at 6-month assessment. There were 2.3–3.3 times greater odds of meeting response and remission criteria in ADepT relative to CBT for the co-primary depression and wellbeing outcomes. Similar findings emerged for secondary outcomes, with numerically greater odds of meeting all criteria in ADepT relative to CBT. Odds ratio 95% CIs did not cross one for: combined depression/wellbeing and SHAPS anhedonia response and remission; MASQ-S30 distress response; and SCID six-month and sustained remission, which would meet conventional frequentist criteria for ADepT being superior to CBT.

**Table 4:** Remission, Response and IAPT metric analyses.

	CBT	ADepT	Odds ratio (95% CI)
**Remission 6m**			
SCID depression	0.56	0.80	3.25 (1.09, 9.87)
PHQ-9 depression	0.49	0.69	2.48 (0.88, 7.03)
WEMWBS wellbeing	0.38	0.56	2.16 (0.82, 5.70)
PHQ-9/WEMWBS	0.27	0.53	3.10 (1.13, 8.50)
HDRS depression	0.39	0.60	2.41 (0.87, 6.75)
SHAPS anhedonia	0.22	0.47	3.16 (1.07, 9.21)
PANAS positive affect	0.43	0.68	2.66 (0.98, 7.24)
PANAS negative affect	0.49	0.71	2.69 (0.91, 7.92)
MASQ-S30 anhedonia	0.24	0.47	2.83 (0.98, 8.17)
MASQ-S30 general distress	0.16	0.27	1.86 (0.60, 5.81)
**Response 6m**			
PHQ-9 depression	0.46	0.66	2.34 (0.86, 6.36)
WEMWBS wellbeing	0.41	0.63	2.64 (0.94, 7.39)
PHQ-9/WEMWBS	0.30	0.59	3.42 (1.20, 9.87)
HDRS depression	0.58	0.62	1.06 (0.41, 2.77)
SHAPS anhedonia	0.36	0.63	3.06 (1.11, 8.58)
PANAS positive affect	0.54	0.66	1.60 (0.57, 4.53)
PANAS negative affect	0.51	0.75	2.83 (0.98, 8.17)
MASQ-S30 anhedonia	0.33	0.56	2.77 (0.98, 7.85)
MASQ-S30 general distress	0.43	0.73	4.18 (1.34, 13.07)
**NTTad 6m**			
Reliable Improvement	0.68	0.80	1.51 (0.52, 4.35)
Recovery	0.38	0.55	2.01 (0.76, 5.26)
Reliable Recovery	0.38	0.55	2.12 (0.81, 5.53)
**Sustained Remission**			
SCID depression at 6m, 12m and 18m	0.42	0.72	3.16 (1.03, 14.01)

Note: SCID = Structured clinical interview for diagnosis; PHQ-9 = Patient health questionnaire; WEMWBS = Warwick edinburgh mental wellbeing scale; HDRS = Hamilton depression rating scale; SHAPS = Snaith hamilton pleasure scale; PANAS = Positive and negative affect schedule; MASQ-S30=Mood and anxiety symptom questionnaire-short form; NTTad = NHS Talking therapies for anxiety and depression. Proportions are number of clients in each arm meeting criteria (using complete case data); odds ratios are estimates from intent-to-treat binary logistic regressions (using multiple imputation to simulate missing values); odds ratios >1 indicate greater rates of meeting criteria in ADepT than CBT and scores <1 indicate greater rates of meeting criteria in CBT than ADepT.

**Fig. 2:**
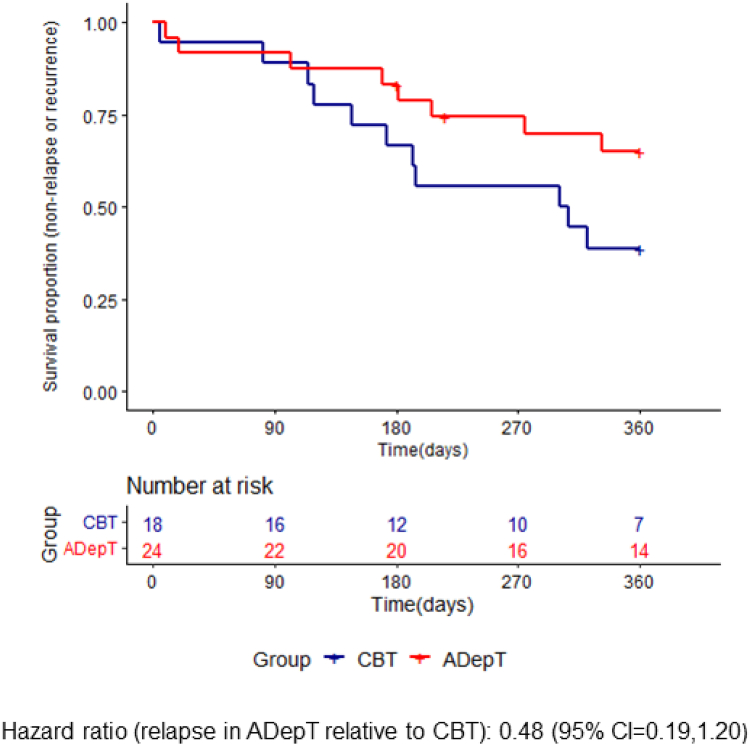
Survival plot examining depressive relapse in the CBT and ADepT arms for the subset of individuals who had recovered at 6m. Note: CBT = Cognitive behavioural therapy; ADepT = Augmented depression therapy.

Of those who had remitted at 6-month assessment, LIFE interview follow-up data to include in survival analyses were available for 18/20 individuals receiving CBT (11 [61%] of whom relapsed) and 24/28 individuals receiving ADepT (8 [33%] of whom relapsed) (Fig. 2). Cox's regression estimated participants were approximately half as likely to relapse following ADepT than CBT, hazard ratio = 0.48 (95% CI = 0.19, 1.20).

Benchmarking analyses suggested ADepT within-arm continuous effect sizes at 6 months were numerically larger, and rates of remission and response were equivalent or higher, relative to CBT and BA delivered in the COBRA^18^ and COBALT^34^ trials (SOM Tables S14 and S15). Comparison to meta-analytic findings^5^ also revealed rates of SCID diagnostic remission at 6 months were greater following ADepT (80%) than is typically observed following other depression psychotherapies (≈60%). CBT in the present trial resulted in broadly similar within-arm effect sizes, and rates of remission and response, to other active therapies reported in the COBRA^18^ and COBALT^34^ trials and to meta-analytic findings.^5^

**Fig. 3:**
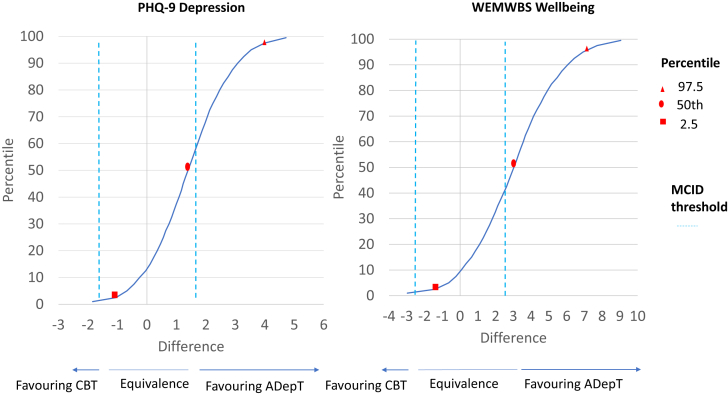
Probability of difference in depression and wellbeing outcomes between treatment arms in six-month Bayesian analyses. Note: CBT = Cognitive behavioural therapy; ADepT = Augmented depression therapy; PHQ-9 = Patient health questionnaire; WEMWBS = Warwick Edinburgh mental wellbeing scale. Analyses run on complete case data. Red shapes indicate the Bayesian 95% credibility interval. Blue dotted lines indicate the minimum clinically important difference threshold (MCID; 1.7 points for PHQ-9, 2.8 points for WEMWBS). Where curves cross the right hand blue dotted line, ADepT is MCID superior to CBT; where curves cross the left hand blue dotted line, CBT is MCID superior to ADepT. Where curves cross to the right of zero, ADepT is superior to CBT at any value; where curves cross to the left of zero, CBT is superior to ADepT at any value.

Fig. 3 summarises Bayesian analyses on PHQ-9 and WEMWBS outcomes at 6-month assessment (plotting the probability of difference in outcomes if new clients were treated with ADepT rather than CBT). For the PHQ-9, 87% of participants would show numerically greater improvement (and 60% would show at least a MCID advantage) in ADepT relative to CBT. For the WEMWBS, 92% of participants would show numerically greater improvement (and 50% would show at least a MCID advantage) in ADepT relative to CBT. On both outcomes, only 1% of clients would show at least a MCID advantage in CBT relative to ADepT. Comparable results favouring ADepT over CBT emerged in other secondary analyses specified in the trial protocol paper (SOM Section 5).

### Health economic proof of concept

**Fig. 4:**
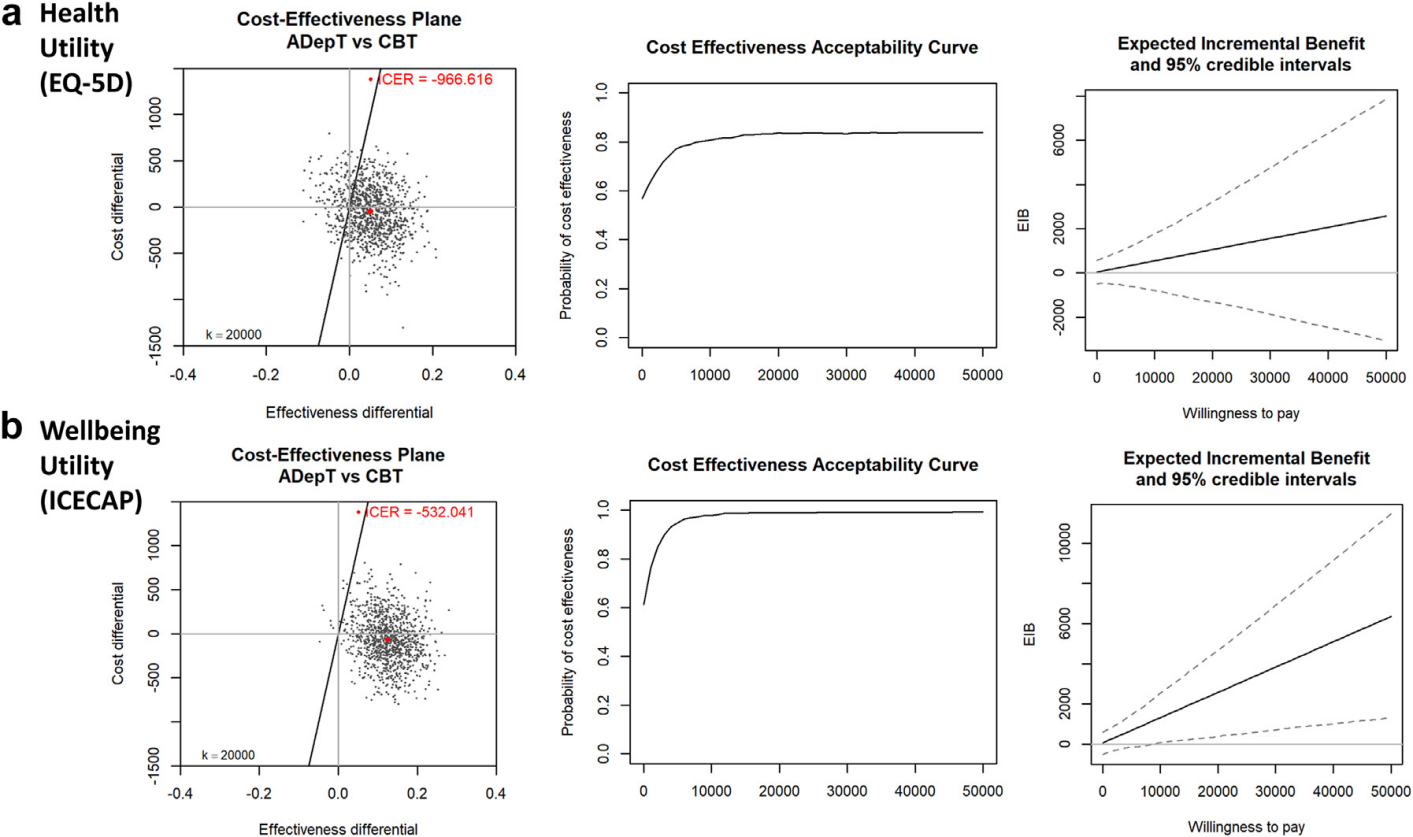
Cost effectiveness planes, expected incremental benefit plots, and cost effectiveness acceptability curves in 18m analyses for EuroQol measure of health status (EQ-5D) health utility (a) and ICEpop capability measurement for adults (ICECAP) wellbeing utility (b). Note: CBT = Cognitive behavioural therapy; ADepT = Augmented depression therapy. Analyses run on bootstrapped distributions from complete case data. In each panel, the left–hand graph is a scatterplot of cost and effectiveness pairs for ADepT versus CBT on a cost-effectiveness plane (with a £20,000 willingness to pay threshold), the middle graph is an expected incremental benefit plot showing the monetary benefit of ADepT relative to CBT at different willingness to pay thresholds (with 95% credibility intervals), and the right–hand graph is a cost effectiveness acceptability curve showing the probability ADepT is cost-effective relative to CBT at different willingness to pay thresholds. A negative incremental cost effectiveness ratio (ICER) value in each cost effectiveness plane reflects lower costs and greater QALYs in ADepT relative to CBT.

Complete QALY and cost data were available for 62/82 (76%) participants (32/41 [78%] CBT, 30/41 [73%] ADepT) for post hoc cost effectiveness analyses. There were two outliers in the CBT arm with greater health care utilization in the 6 months prior to entering the trial (£35K and £44K respectively). There were no outliers in health care utilization at subsequent follow-ups. Costs were broadly comparable between arms. Health (EQ-5D) and wellbeing (ICECAP) utility was greater in ADepT than CBT, with the 95% CI not crossing zero for wellbeing (Table 5). Costs were lower and QALY gains greater following ADepT than CBT, generating an ICER of -£967 for health utility and -£532 for wellbeing utility. The point estimate and a majority of scatter points in the cost-effectiveness planes fell in the SE dominant quadrant and on the side of the £20K willingness-to-pay per QALY line favouring ADepT over CBT for both utility measures (Fig. 4). Similarly, ADepT was likely cost-effective relative to CBT on both utility measures when inspecting CEAC plots (≈80% probability for health utility and >97.5% probability for wellbeing utility at the £20K willingness to pay threshold) and (net) EIB plots. Comparable findings emerged in secondary sensitivity analyses analysing cost-effectiveness at 6 months (SOM Section 6).

**Table 5:** Costs and QALYs at 18-month assessment point in the CBT and ADepT arms.

	CBT	ADepT	Difference (ADepT—CBT)
**Baseline**			
ADSUS costs	£2378 (£8618)	£646 (£984)	−£1722 (−£4420, £975)
**18-month**			
*Costs* per *participant (£)*			
Intervention costs	£2505 (£459)	£2514 (£488	
Broader health care utilization	£1055 (£952)	£988 (£1125)	
Total	£3703 (£1010)	£3697 (£1160)	−£40 (£−605, £525)
*QALY estimates*			
EQ-5D	0.999 (0.259)	1.000 (0.313)	0.053 (−0.053.0.160)
ICECAP	0.904 (0.235)	0.970 (0.308)	0.111 (0.013, 0.208)

Note: CBT = Cognitive behavioural therapy; ADepT = Augmented depression therapy; QALY = Quality adjusted life year; EQ-5D = EuroQol measure of health status (a measure of health utility); ICECAP= ICEpop capability measurement for adults (a measure of wellbeing utility). Cost and QALY estimate are mean (SD) values from complete case data; difference scores are mean (95% CI) values from intent-to-treat linear regressions (using multiple imputation to simulate missing values).

## Discussion

This is the first pilot randomised controlled trial evaluating the effects of Augmented Depression Therapy (ADepT^13^^,^^15^) in repairing anhedonic depression. Overall, the findings demonstrate the feasibility of testing this intervention relative to CBT to treat anhedonic depression in a subsequent definitive randomised controlled trial. It was possible to recruit and randomise individuals and there were adequate rates of data completeness at follow-up. Treatments were rated as acceptable and satisfactory by clients. A majority of clients completed an adequate dose of treatment, with low dropout rates. Therapists could be trained to deliver the ADepT and CBT protocols competently. There were no trial- or treatment-related serious adverse events and no broader evidence of harms. The sample recruited were primarily severe cases with co-morbid anxiety, so are representative of the complex presentations typically seen in routine clinical practice. All pre-specified feasibility continuation rules were met, suggesting a definitive randomised controlled trial could be run.

Clinical proof of concept findings suggest that ADepT is likely an effective treatment for anhedonic depression. Within-arm analyses showed ADepT and CBT both led to clinically meaningful improvements in depression, wellbeing, and all other secondary outcomes (including measure of anhedonia and the PVS). Inspection of between-group confidence intervals revealed that ADepT was not MCID worse than, and showed potential to be MCID superior to, CBT on the primary wellbeing and depression outcomes and on nearly all secondary outcomes (including anhedonia and PVS outcomes) at 6-month assessment. These gains were maintained when considering cumulative levels of each outcome across the 18-month trial follow-up. Comparable results emerged when considering rates of remission and response. Bayesian analyses on 6-month outcomes indicated that a majority of individuals showed at least a MCID advantage on depression and wellbeing outcomes receiving ADepT rather than CBT (with <1% of individuals showing a MCID advantage of CBT over ADepT). ADepT continuous and binary outcomes were generally superior to other active psychotherapies observed in previous trials, including BA and CBT evaluated in the COBALT and COBRA trials.^4^^,^^18^^,^^34^

The probability of ADepT cost-effectiveness relative to CBT was >97.5% on wellbeing and ≈80% on health QALY measures at the £20K QALY willingness to pay threshold recommended by UK NICE, with ADepT dominating CBT for both utility measures. As a point of comparison, the recent COBRA trial^18^ concluded BA was cost-effective relative to CBT on the basis that it was equivalently effective to CBT but was cheaper due to delivery via a low intensity (as opposed to high intensity) therapy workforce (with ≈80% probability of cost-effectiveness). The ICECAP wellbeing measure was more sensitive than the EQ-5D health measure in demonstrating differences in cost-effectiveness; it remains to be established if this is because it is a more sensitive tool in all mental health contexts or only when evaluating interventions that have a strong wellbeing emphasis.

The individual and societal burden of depression is such that even relatively small differences in treatment outcomes are likely clinically meaningful (ds >0.24 on continuous outcomes; >10% difference on rate outcomes). These pilot trial results suggest ADepT has potential to lead to enhanced outcomes relative to current standard practice (CBT) that exceed these thresholds for a meaningful effect. If the current clinical and health economic outcomes of ADepT can be replicated in a subsequent definitive trial, this would represent a stepwise improvement in depression treatment outcomes with potential economic advantages of implementing ADepT in health care settings like NTTad.

Intervention efficacy is ultimately more important than intervention novelty and it is desirable for novel treatments to incorporate proven elements from existing treatment approaches. ADepT has overlap with BA and the early stages of CBT in that it involves activating clients towards rewarding activities. The values focus of ADepT is also similar to Acceptance and Commitment Therapy (ACT). However, ADepT differs from these approaches in that it sets wellbeing as the primary goal and frames depression as a barrier to achieving this goal; utilises a positively-oriented, future-directed, and solution-focused therapy style to draw out client strengths during moments of resilience and thriving; targets a range of ‘pleasure blocking’ psychological mechanisms identified from basic-science research that are not explicit foci in these other therapies; and has a more explicit recovery focus. ADepT is one of a class of emerging transdiagnostic interventions that target PVS disturbance^14^^,^^35^^,^^36^ and that show potential to lead to enhanced treatment outcomes across a range of mental health conditions.

A number of limitations should be held in mind. There is ongoing debate about the validity of conducting proof of concept analyses on pilot trials. We would argue that within-arm frequentist analyses, examination of confidence intervals from between-group analyses, and inspection of between-group Bayesian credibility intervals provide an appropriate way to estimate ‘signal’ from well-designed pilot trials (maximising value of information gained and minimising research waste by ensuring only promising interventions are taken to definitive trial). In this case, we believe our findings suggest a substantial ‘signal’ in respect of the potential of ADepT. Care was delivered under ideal conditions in a research clinic setting by experienced therapists and the sample recruited was primarily of White British ethnic origin (reflecting the demographics of the Devon region). It remains an open question as to whether ADepT will be clinically effective when delivered in more pragmatic, real world setting by routine workforces and when treating a more diverse population. The health economic analyses were post hoc, only measured broader service use-categories captured by the AD-SUS, and did not utilise modelling to extrapolate beyond the eighteen-month follow-up. This means caution should be taken generalising these pilot results and the longer-term cost-effectiveness of ADepT has yet to be established. No systematic steps were taken to match the two treatments in terms of number of components, skills or activities and it is possible differences in these factors could be driving results. Due to the nature of the interventions, care was delivered open label, although researchers conducting assessments were blind to treatment allocation. While the trial was designed and delivered in such a way as to minimise sources of bias, the study is nevertheless vulnerable to allegiance bias. The pre-specified analyses did not adjust for potential therapist clustering effects. However, secondary sensitivity analyses that included therapist as a random factor within mixed models found negligible evidence of therapist clustering (SOM Section 3). The therapists in the current trial were all experienced and closely supervised, so it is conceivable there may be more marked therapist clustering effects in pragmatic settings. Potentially biasing clinical and health economic proof of concept results, there were slightly greater rates of missing follow-up data in ADepT relative to CBT and data was more likely to be missing at follow-up in those who did not fully engage with treatment (meaning complete case data approximates per protocol data). Partially mitigating this, key proof-of-concept analyses used multiple imputation and were run on an intent-to-treat basis (although the imputation models did not make adjustments for potential clustering effects). A future definitive trial should account for potential therapist clustering effects when setting the target sample size, consider using analytic methods that adjust for therapist clustering, and attempt to minimise rates of missing data. The analyses examining if the between-group effect size confidence intervals crossed into the region where therapies differed from each other above a MCID threshold should not be viewed as formal non-inferiority analyses, as the trial did not a priori adopt a non-inferiority design and the non-inferiority margin was not set prospectively.

Overall, the ADepT intervention and the trial evaluating it were found to be feasible, it was demonstrated that ADepT has potential to be clinically superior to (and is not worse than) CBT, and health economic analyses indicated ADepT may be cost-effective relative to CBT. A large multicentre pragmatic trial powered to detect minimum clinically important change in depression and wellbeing that builds on the successful procedures in the current study is warranted to test definitively if ADepT is clinically and health-economically superior compared to routine CBT in the treatment of anhedonic depression.

## Contributors

The authors made the following contributions to the study. BD: conceptualisation, data curation, formal analysis, funding acquisition, investigation, methodology, project administration, supervision, writing–original draft, and writing—review & editing; EW: data curation, methodology, project administration, investigation, writing review & editing; LW: data curation, methodology, project administration, investigation, formal analysis, writing—review & editing; FW: data curation, project administration, investigation; NR: conceptualisation, funding acquisition, methodology, writing—review & editing; AP, MK.,CC., RS: investigation; KW, NM, NG, CO, AS, JC., WK: conceptualisation, funding acquisition, project methodology, supervision, writing—review & editing. BD and LW both accessed and verified the data.

## Data sharing statement

Deidentified participant data with a data dictionary and related study documents will be made available on request from the first author (BD), subject to approval of a proposal and establishment of a signed data access agreement.

## Declaration of interests

BD has a book contract with Guilford Press to write the ADepT treatment manual and receives occasional payment or honoraria (including support for attending meetings) for delivering workshops and talks on ADepT. All other authors declare no competing interests.

## Appendix A Supplementary data

Appendix One Reporting Checklists for second revision

SOMS lancet revision

## References

[1] Judd L.L. (1997). The clinical course of unipolar major depressive disorders. Arch Gen Psychiatry.

[2] Kessler R.C., Berglund P., Demler O. (2003). The epidemiology of major depressive disorder - results from the national comorbidity survey replication (NCS-R). JAMA.

[3] Konig H., Konig H.H., Konnopka A. (2020). The excess costs of depression: a systematic review and meta-analysis. Epidemiol Psychiatr Sci.

[4] Cuijpers P., Karyotaki E., Weitz E., Andersson G., Hollon S.D., van Straten A. (2014). The effects of psychotherapies for major depression in adults on remission, recovery and improvement: a meta-analysis. J Affect Disord.

[5] Vittengl J.R., Clark L.A., Dunn T.W., Jarrett R.B. (2007). Reducing relapse and recurrence in unipolar depression: a comparative meta-analysis of cognitive-behavioral therapy's effects. J Consult Clin Psychol.

[6] Beck A.T., Rush A.J., Shaw B.F., Emery G. (1979). Cognitive therapy of depression.

[7] Insel T., Cuthbert B., Garvey M. (2010). Research domain criteria (RDoC): toward a new classification framework for research on mental disorders. Am J Psychiatry.

[8] Dunn B.D., German R.E., Khazanov G., Xu C.L., Hollon S.D., DeRubeis R.J. (2020). Changes in positive and negative affect during pharmacological treatment and cognitive therapy for major depressive disorder: a secondary analysis of two randomized controlled trials. Clin Psych Sci.

[9] Alsayednasser B., Widnall E., O'Mahen H. (2020). How well do cognitive behavioural therapy (CBT) and behavioural activation (BA) for depression repair anhedonia: a secondary analysis of the COBRA randomised controlled trial. Behav Res Ther.

[10] Dunn B.D., Gruber J. (2019). Oxford handbook of positive emotion and psychopathology.

[11] Zimmerman M., McGlinchey J.B., Posternak M.A., Friedman M., Attiullah N., Boerescu D. (2006). How should remission from depression be defined? The depressed patient's perspective. Am J Psychiatry.

[12] Widnall E., Price A., Trompetter H., Dunn B.D. (2020). Routine cognitive behavioural therapy for anxiety and depression is more effective at repairing symptoms of psychopathology than enhancing wellbeing. Cog Ther Res.

[13] Dunn B.D., Widnall E., Reed N., Owens C., Campbell J., Kuyken W. (2019). Bringing light into darkness: a multiple baseline mixed methods case series evaluation of Augmented Depression Therapy (ADepT). Behav Res Ther.

[14] Sandman C.F., Craske M.G., Pizagailli D.A. (2022).

[15] Dunn B.D., Widnall E., Reed N. (2019). Evaluating Augmented Depression Therapy (ADepT): study protocol for a pilot randomised controlled trial. Pilot Feasibility Stud.

[16] First M.B., Spitzer M., Gibbon M., Williams J.B.W. (1994).

[17] Kroenke K., Spitzer R.L., Williams J.B.W. (2001). The PHQ-9 - validity of a brief depression severity measure. J Gen Intern Med.

[18] Richards D.A., Ekers D., McMillan D. (2016). Cost and outcome of behavioural activation versus cognitive behavioural therapy for depression (COBRA): a randomised, controlled, non-inferiority trial. Lancet.

[19] Blackburn I.M., James I.A., Milne D.L. (2001). The revised cognitive therapy scale (Cts-R): psychometric properties. Behav Cog Psychother.

[20] Tennant R., Hiller L., Fishwick R. (2007). The Warwick-Edinburgh mental well-being scale (WEMWBS): development and UK validation. Health Qual Life Outcome.

[21] Spitzer R.L., Kroenke K., Williams J.B.W., Lowe B. (2006). A brief measure for assessing generalized anxiety disorder - the GAD-7. Arch Inten Med.

[22] Snaith R.P., Hamilton M., Morley S., Humayan A., Hargreaves D., Trigwell P. (1995). A scale for the assessment of hedonic tone: the Snaith-Hamilton Pleasure Scale. Br J Psychiatry.

[23] Watson D., Clark L.A., Tellegen A. (1988). Development and validation of brief measures of positive and negative Affect - the PANAS scales. J Pers Soc Psychol.

[24] Wardenaar K.J., van Veen T., Giltay E.J., de Beurs E., Penninx B.W.J.H., Zitman F.G. (2010). Development and validation of a 30-item short adaptation of the mood and anxiety symptoms Questionnaire (MASQ). Psychiatry Res.

[25] IAPT Phobia Scale (2008).

[26] Mundt J.C., Marks I.M., Shear M.K., Greist J.H. (2002). The work and social adjustment scale: a simple measure of impairment in functioning. Br J Psychiatry.

[27] Williams J.B.W. (1988). A structured interview guide for the hamilton depression rating-scale. Arch Gen Psychiatry.

[28] Shear M.K., Vander Bilt J., Rucci P. (2001). Reliability and validity of a structured interview Guide for the Hamilton anxiety rating scale (SIGH-A). Depress Anxiety.

[29] Keller M.B., Lavori P.W., Friedman B. (1987). The longitudinal interval follow-up evaluation - a comprehensive method for assessing outcome in prospective longitudinal-studies. Arch Gen Psychiatry.

[30] Bower P., Byford S., Sibbald B. (2000). Randomised controlled trial of non-directive counselling, cognitive-behaviour therapy, and usual general practitioner care for patients with depression. II: cost effectiveness. BMJ.

[31] Herdman M., Gudex C., Lloyd A. (2001). Development and preliminary testing of the new five-level version of EQ-5D (EQ-5D-5L). Qual Life Res.

[32] Al-Janabi H., Flynn T.N., Coast J. (2012). Development of a self-report measure of capability wellbeing for adults: the ICECAP-A. Qual Life Res.

[33] Cuijpers P., Turner E.H., Koole S.L., van Dijke A., Smit F. (2014). What is the threshold for a clinically relevant effect? The case of major depressive disorders. Depress Anxiety.

[34] Wiles N.J., Thomas L., Abel A. (2014). Cognitive behavioural therapy as an adjunct to pharmacotherapy for primary care based patients with treatment resistant depression: results of the CoBalT randomosed controlled trial. Lancet.

[35] Craske M.G., Meuret A.E., Ritz T., Treanor M., Dour H., Rosendfield D. (2019). Positive affect treatment for depression and anxiety: a randomized clinical trial for a core feature of anhedonia. J Consult Clin Psychol.

[36] Geschwind N., Arntz A., Bannink F., Peeters F. (2019). Positive cognitive behaviour therapy in the treatment of depression: a randomized order within-subject comparison with traditional cognitive behaviour therapy. Behav Res Ther.

