# Supplementary material for: Preliminary clinical and cost effectiveness of augmented depression therapy versus cognitive behavioural therapy for the treatment of anhedonic depression (ADepT): a single-centre, open-label, parallel-group, pilot, randomised, controlled trial
Source: eClinicalMedicine. 2023 Jul 13;61:102084. doi: 10.1016/j.eclinm.2023.102084 (PMC10388573; doi:10.1016/j.eclinm.2023.102084)
Supplement: Appendix One Reporting Checklists for second revision [file mmc1.docx]

# **Appendix 1a: Reporting checklist for a pilot randomised trial.**

|  |  | Reporting Item | Page Number |
| --- | --- | --- | --- |
| **Title and Abstract** |  |  |  |
| Title | 1a | Identification as a pilot or feasibility randomised trial in the title. | p.1 |
| Abstract | 1b | Structured summary of pilot trial design, methods, results and conclusions (for specific guidance see CONSORT abstract extension for pilot trials). | p.2 |
| **Introduction** |  |  |  |
| Background and objectives | 2a | Scientific background and explanation of rationale for future definitive trial, and reasons for randomized pilot trial. | p.4-5 |
| Background and objectives | 2b | Specific objectives or research questions for pilot trial. | p.5 |
| **Methods** |  |  |  |
| Trial design | 3a | Description of pilot trial design (such as parallel, factorial) including allocation ratio. | p.5-6 |
| Trial design | 3b | Important changes to methods after pilot trial commencement (such as eligibility criteria), with reasons. | SOM S3c |
| Participants | 4a | Eligibility criteria for participants. | p.6 |
| Participants | 4b | Settings and locations where the data were collected. | p.5 |
|  | 4c | How participants were identified and consented. | p.6 |
| Interventions | 5 | The experimental and control interventions for each group with sufficient details to allow replication, including how and when they were actually administered. | p.7, SOM S2 |
| Outcomes | 6a | Completely defined prespecified assessments or measurements to address each pilot trial objective specified in 2b, including how and when they were assessed. | p.7-10, SOM S3a, SOM 4 |
| Outcomes | 6b | Any changes to pilot trial assessments or measurements after the pilot trial commenced, with reasons. | SOM S3c |
|  | 6c | If applicable, prespecified criteria used to judge whether, or how, to proceed with future definitive trial. | p.5 |
| Sample size | 7a | Rationale for numbers in the pilot trial. | p.5-6 |
| Sample size | 7b | When applicable, explanation of any interim analyses and stopping guidelines. | NA |
| Randomization - Sequence generation | 8a | Method used to generate the random allocation sequence. | p.6 |
| Randomization - Sequence generation |  | Type of randomization(s); details of any restriction (such as blocking and block size). | p.6 |
| Randomization - Allocation concealment mechanism | 9 | Mechanism used to implement the random allocation sequence (such as sequentially numbered containers), describing any steps taken to conceal the sequence until interventions were assigned. | p.6 |
| Randomization – Implementation | 10 | Who generated the allocation sequence, who enrolled participants, and who assigned participants to interventions. | p.6 |
| Blinding | 11a | If done, who was blinded after assignment to interventions (for example, participants, care providers, those assessing outcomes) and how. | p.7 |
| Blinding | 11b | If relevant, description of the similarity of interventions. | NA |
| Statistical methods | 12a | Methods used to address each pilot trial objective whether qualitative or quantitative. | p.8-10, SOM S3a, SOM 4 |
| **Results** |  |  |  |
| Participant flow diagram (strongly recommended) | 13a | For each group, the numbers of participants who were approached and/or assessed for eligibility, randomly assigned, received intended treatment, and were assessed for each objective. | Figure One |
| Participant flow | 13b | For each group, losses and exclusions after randomization, together with reason. | Figure One |
| Recruitment | 14a | Dates defining the periods of recruitment and follow-up. | p.6 |
| Recruitment | 14b | Why the pilot trial ended or was stopped. | NA |
| Baseline data | 15 | A table showing baseline demographic and clinical characteristics for each group. | Table One |
| Numbers analysed | 16 | For each group, number of participants (denominator) included in each analysis and whether the analysis was by original assigned groups. | Throughout |
| Outcomes and estimation | 17a | For each objective, results including expressions of uncertainty (such as 95% confidence interval) for any estimates. If relevant, these results should be randomized by group. | Throughout |
| Ancillary analyses | 18 | Results of any other analyses performed that could be used to inform the future definitive trial. | SOM throughout |
| Harms | 19 | All important harms or unintended effects in each group (For specific guidance see CONSORT for harms). | p.11, SOM S4 rule 4 |
|  | 19a | If relevant, other important unintended consequences. | NA |
| **Discussion** |  |  |  |
| Limitations | 20 | Pilot trial limitations, addressing sources of potential bias and remaining uncertainty about feasibility. | p.14-15 |
| Generalisability | 21 | Generalisability (applicability) of pilot trial methods and findings to future definitive trial and other studies. | p.13 to p.15 |
| Interpretation | 22 | Interpretation consistent with pilot trial objectives and findings, balancing potential benefits and harms, and considering other relevant evidence. | p.13 to p.15 |
|  | 22a | Implications for progression from pilot to future definitive trial, including any proposed amendments. | p.13 to p.15, SOM S4 |
| Registration | 23 | Registration number for pilot trial and name of trial registry. | p.2 |
| **Other information** |  |  |  |
| Protocol | 24 | Where the pilot trial protocol can be accessed, if available. | p.5 |
| Funding | 25 | Sources of funding and other support (such as supply of drugs), role of funders. | p.2, p.10 |
|  | 26 | Ethical approval or approval by research review committee, confirmed with reference number. | p.6 |

**Appendix 1b: CONSORT guidance for reporting nonpharmacological treatment (NPT) trials**

| Paper section and topic | Checklist number | Extension for nonpharmacological trials | Page reported |
| --- | --- | --- | --- |
| **Methods** |  |  |  |
| Outcomes | 3a | When applicable, how care providers were allocated to each trial group. | SOM S4, rule 5 |
| Eligibility criteria for participants | 4a | When applicable, eligibility criteria for centers and for care providers. | SOM S4, rule 5 |
| Interventions | 5 | Precise details of both the experimental treatment and the comparator. | SOM S2 for ADepT |
|  | 5a | Description of the different components of the interventions  and, when applicable, description of the procedure for tailoring the intervention to individual participants. | SOM S2 for ADepT |
|  | 5b | Details of whether and how the interventions were standardized. | SOM S2 for ADepT |
|  | 5c | Details of whether and how adherence of care providers to the protocol was assessed or enhanced. | SOM S4, rule 5 |
|  | 5d | Details of whether and how adherence of participants to interventions was assessed or enhanced. | SOM S4, rule 3 |
| Sample Size | 7a | When applicable, details of whether and how the clustering by care providers or centers was addressed. | p.6, SOM S3, sensitivity analyses, |
| Numbers analyzed | 11a | If done, who was blinded after assignment to the interventions (e.g. participants, care providers, those administering co-interventions, those assessing outcomes) and how. | p.6 |
|  | 11c | If blinding was not possible, description of any attempts to limit bias. | NA |
| Statistical Methods | 12a | When applicable, details of whether and how the clustering by care providers of centers was addressed. | SOM S3 sensitivity analyses, |
| **Results** |  |  |  |
| Participant flow | 13a | The number of care provider or centers performing the intervention in each group and the number of patients treated by each care provider or in each center. | SOM S4 rule 5, SOM Table S9 |
|  | 13c | For each group, the delay between randomization and the initiation of the intervention. | SOM S4 p.21 |
|  | New | Details of the experimental treatment and comparator as they were implemented. | p.10, SOM S4 rule three |
| Baseline data | 15 | When applicable, a descriptor of care providers (case volume, qualification, expertise, etc.) and centres (volume) in each group. | Table 1, SOM S4 rule 5, SOM Table S9 |
|  |  |  |  |
| **Discussion** |  |  |  |
| Limitations | 20 | In addition, take into account the choice of the comparator, lack of or partial blinding, and unequal expertise of care providers or centers in each group. | p.14-15 |
|  | 21 | Generalizability (external validity) of the trial findings according to the intervention, comparators, patients, and care providers and centers involved in the trial. | p.14-15 |

**Appendix 1c: CONSORT guidance for abstracts reporting RCTs Assessing NPTs**

| Paper section and topic | Extension for nonpharmacological trials | Completed? |
| --- | --- | --- |
| Participants | When applicable, report eligibility criteria for centers where the intervention is performed and for care providers. | N/A |
| New | Report any important changes to the intervention delivered from what was planned. | N/A |

**Appendix 1d: Consort guidance on abstract for reporting pilot trials**

| Item | Criteria | Included |
| --- | --- | --- |
| Title | Identification of study as randomized pilot or feasibility trial | Y |
| Trial Design | Description of pilot trial design (e.g. parallel, cluster) | Y |
| **Methods** |  |  |
| Participants | Eligibility criteria for participants and the settings where the pilot trial was conducted | Y |
| Interventions | Interventions intended for each group | Y |
| Objectives | Specific objectives of the pilot trial | Y |
| Outcome | Prespecified assessment or measurement to address the pilot trial objectives* | Y |
| Randomization | How participants were randomized to interventions | Y |
| Blinding (masking) | Whether or not participants, caregivers and those assessing the outcomes were blinded to group assignment | Y |
| **Results** |  |  |
| Numbers randomized | Number of participants screened and randomized to each group for the pilot trial objectives | Y |
| Recruitment | Trial status+ | NA |
| Numbers analyzed | Number of participants analysed in each group for the pilot trial objectives* | Y |
| Outcome | Results for the pilot objectives, including any expressions of uncertainty* | Y |
| Harms | Important adverse events or side effects | Y |
| Conclusions | General interpretation of the results of pilot trial and their implications for future definitive trial | Y |
| Trial Registration | Registration number for pilot trial and name of trial register | Y |
| Funding | Source of funding for pilot trial | Y |

*space permitting, list all pilot trial objectives and give the results for each. Otherwise, report those that are a priori agreed as the most important to the decision to proceed with the future definitive RCT.

+for conference abstracts

**Appendix 1e: Consolidated Health Economic Reporting Standards (CHEERS) checklist**

| Paper section and topic | Item | Extension for nonpharmacological trials | Page reported |
| --- | --- | --- | --- |
| Title | 1 | Identify the study as an economic evaluation and specify the interventions being compared. | p.1 |
| Abstract | 2 | Provide a structured summary that highlights context, key methods, results and alternative analyses. | p.2 |
| **Introduction** |  |  |  |
| Background and objectives | 3 | Give the context for the study, the study questions and its practical relevance for decision making in policy or practice. | p.4-5 |
| **Methods** |  |  |  |
| Health economic analysis plan | 4 | Indicate whether a health economic analysis plan was developed and where available. | SOM Section 3b |
| Study population | 5 | Describe characteristics of the study population (such as age range, demographic, socioeconomic, or clinical characteristics). | Table 1 |
| Setting and location | 6 | Provide relevant contextual information that may influence findings. | p.5 |
| Comparators | 7 | Describe the interventions or strategies being compared and why chosen. | p.4 -6, SOM Section 2 |
| Perspective | 8 | State the perspective(s) adopted by the study and why chosen. | p.9, SOM Section 3b |
| Time horizon | 9 | State the time horizon for the study and why appropriate. | p.9, SOM Section 2b |
|  |  |  |  |
| Discount rate | 10 | Report the discount rate(s) and the reason chosen. | SOM Section 3b |
| Selection of outcomes | 11 | Describe what outcomes were used as the measure(s) of benefits(s) and harm(s). | p.8, SOM Section 3b |
| Measurement of outcomes | 12 | Describes how outcomes used to capture benefit(s) and harm(s) were measured. | p.8, SOM Section 3b |
| Valuation of outcomes | 13 | Describe the population and methods used to measure and value outcomes. | SOM Section 3b |
| Measurement/valuation of resources and costs | 14 | Describe how costs were valued. | SOM Section 3b |
| Currency, price date, and conversion | 15 | Report the dates of the estimated resource quantities and unit costs, plus the currency and year of conversion. | SOM Section 3b |
| Rationale and description of model | 16 | If modelling is used, describe in detail and why used. Report if the model is publicly available and where it can be accessed. | N/A |
| Analytics and assumptions | 17 | Describe any methods for analysing or statistically transforming data, any extrapolation approaches, and approaches for validating model used. | SOM Section 3b |
| Characterising heterogeneity | 18 | Describe any methods used for estimating how the results of the study vary for subgroups. | N/A |
| Characterising distributional effects | 19 | Describe how impacts are distributed across different individuals or adjustments made to reflect priority populations. | N/A |
| Characterising uncertainty | 20 | Describe methods to characterize any sources of uncertainty in the analysis. | p.10, SOM Section 3b |
| Approach to engagement with patients and others affected by the study | 21 | Describe any approaches to engage patients or service recipients, the general public, communities, or stakeholders (such as clinicians or payers) in the design of the study. | SOM Section 1 |
| **Results** |  |  |  |
| Study parameters | 22 | Report all analytic inputs (such as values, ranges, references) including uncertainty or distributional assumptions. | p.13, Table 5 |
| Summary of main results | 23 | Report the mean values for the main categories of costs and outcomes of interest and summarise them in the most appropriate overall measure. | p.13, Table 5 |
| Effect of uncertainty | 24 | Describe how uncertainty about analytic judgments, inputs, or projections affect findings. Report the effect of choice of discount rate and time horizon, if applicable. | SOM Section 6 |
| Effect of engagement with patients and others affected by the study | 25 | Report on any difference patient/service recipient, general public, community, or stakeholder involvement made to the approach or findings of the study. | SOM Section 1 |
| **Discussion** |  |  |  |
| Study findings, limitations, generalisbility, and current knowledge | 26 | Report key findings, limitations, ethical or equity considerations not captured, and how these could affect patients, policy or practice. | p.14-15 |
| **Other relevant information** |  |  |  |
| Source of funding | 27 | Describe how the study was funded and any role of the funder in the identification, design, conduct and reporting of the analysis. | p.2, p.10 |
| Conflicts of interest | 28 | Report authors conflicts of interest according to journal or International Committee of Medical Journal Editors requirements. | p.16 |

**Appendix 1f: CONSORT guidance for reporting harms**

| Paper section and topic | Checklist number | Description | Page reported |
| --- | --- | --- | --- |
| Title and Abstract | 1 | If the study collected data on harms and benefits, the title or abstract should so state. | p.2 |
| **Introduction** |  |  |  |
| Background | 2 | If the trial address both harms and benefits, the introduction should so state. | p.5 |
| **Methods** |  |  |  |
| Outcomes | 6 | List addressed adverse events with definitions for each (with attention, when relevant, to grading, expected v unexpected events, reference to standardized and validated definitions, and descriptions of new definitions. | SOM S3 rule 4, Table S7 |
| Outcomes | 6 | Clarify how harms-related information was collected (mode of data collection, timing, attribution, methods, intensity of ascertainment, and harms-related monitoring and stopping results, if pertinent). | SOM S3, rule 4 |
| Statistical methods | 12 | Describe plans for presenting and analyzing information on harms (including coding, handling of recurrent events, specification of timing issues, handling of continuous measures, and any statistical analyses). | SOM S3 rule 4 |
| **Results** |  |  |  |
| Participant flow | 13 | Describe for each arm the participant withdrawals that are due to harms and their experiences with the allocated treatments. | SOM S4, rule 3 |
| Numbers analyzed | 16 | Provide the denominator for analyses on harms. |  |
| Outcomes and estimation, Ancillary analyses and adverse events | 17,18,19 | Present the absolute risk per arm and per adverse event type, grade and seriousness, and present appropriate metrics for recurrent events, continuous variables, and scale variables, whenever pertinent. | p.10-11, SOM S4 rule 4 |
|  | 17,18,19 | Describe any subgroup analyses and exploratory analyses for harms. | NA |
| **Discussion** |  |  |  |
| Interpretation, Generalizability, Overall Evidence | 20,21,22 | Provide a balanced discussion of benefits and harms with emphasis on study limitations, generalizability, and other sources of information on harms. | p.13 |
