# Supplementary material for: Preliminary clinical and cost effectiveness of augmented depression therapy versus cognitive behavioural therapy for the treatment of anhedonic depression (ADepT): a single-centre, open-label, parallel-group, pilot, randomised, controlled trial
Source: eClinicalMedicine. 2023 Jul 13;61:102084. doi: 10.1016/j.eclinm.2023.102084 (PMC10388573; doi:10.1016/j.eclinm.2023.102084)
Supplement: SOMS lancet revision [file mmc2.docx]

**Supplementary Online Materials (SOM)**

**1: Patient and public involvement**

The form and content of the ADepT intervention and the trial protocol was co-designed using input from the public and patient involvement lead on the project team (NR), other members of the Lived Experience Group (LEG) at the Mood Disorders Centre, University of Exeter, and qualitative interviews with other service-users. PPI members were involved in ongoing governance of the project, including NR meeting regularly with the PI and attending ADepT project meetings and another LEG member sitting on the TSC/DMEC. UK standards for public involvement in research were followed.

**2: Detailed Description of ADepT Intervention**

Sections from ADepT Treatment Protocol

The following sections are taken from the ADepT training manual given to therapists taking part in the trial. In addition to this skeleton overview, rationale overview, and phase overview, therapists were given detailed session by session guidance and a series of handouts to share with clients.

Skeleton Overview

- ADepT is a cognitively augmented behavioural activation approach. Clients are encouraged to identify values and goals in life and then to make behavioural changes to move towards these goals/values. The focus is on building wellbeing (mood, meaning and social connection) across relationship, vocation, leisure and self-care life domains. Depression is seen as an obstacle to achieve these wellbeing goals that can be skilfully managed (so symptom reduction is only a secondary objective). In other words, clients are supported to move to a position of realistic acceptance and skilful management of their depression so that they can live as well as possible both when well and when symptomatic (e.g., like managing diabetes).
- A graded exposure activation approach is followed, with the assumption that there will be significant avoidance to overcome (triggered both in response to positive and negative feelings/situations). For each action step in the hierarchy, clients are encouraged to consider capability, motivation, and opportunity to carry this out (COM-B framework). Therapy monitors how client maximises opportunities (thriving) and minimises challenges (resilience) when working towards these goals/values (formulating a range of cognitive, interpersonal, somatic and behavioural factors that help and hinder). Therapy then attempts to support clients to change unhelpful mechanisms and bolster existing/alternative adaptive ones. Symptoms of depression that make it hard to follow this activation approach are addressed (sleep, fatigue, cognitive deficits) as necessary. Prioritisation of the four life domains varies from client to client but always ensure that sufficient attention is paid to the self-care domain (including engaging in simple everyday pleasures, nurturing self-compassion and disengaging from counterproductive behaviours like use of substances). Good self-care is seen as a pre-requisite for progress in the other life domains (as working towards values/goals is hard work).
- In addition to working towards valued goals, clients are encouraged to engage in everyday wellbeing enhancing behaviours throughout therapy, identifying routine activities that have the potential to build pleasure, meaning and connection, and practising engaging with these activities mindfully.
- A wellbeing plan going forwards is developed (including setting three-month values consistent goals, routinising everyday wellbeing enhancing activities, and spotting and responding to drops in mood) and there is an extended series of (up to five) follow up sessions to monitor progress with this wellbeing plan.
- There is a strong emphasis on building the capacity of the client to constructively shape the (physical and social environment) around them (getting and giving what is needed from partner, family, friends, employer, community; being able to skilfully articulate to people what they need to be able to function even when depressed).
- There is a strong emphasis on helping clients build a constructive identity towards their depression – an understanding of their mental health that empowers them to manage it effectively. It is possible to experience pockets of wellbeing, to have periods of effective functioning, and to continue to make some goal progress even when depressed. Skilful behavioural management can minimise likelihood, frequency, duration and life impact of future relapses. Depression is seen as something that comes over people not a core part of their identity, with therapists making regular use of externalising metaphors to build this perspective.
- Therapists focus is on reviewing changes in wellbeing, just as much as reductions in depression and anxiety. Therapists attend much as possible on when things have gone well – where resilience was shown and where an opportunity was taken – to thicken a ‘solution focused’ narrative. The intention is to pay equal attention to resilience and thriving, although this will need to be adapted for each clients’ presentation. As much as possible, therapy looks forwards (building positive anticipation) rather than to the past.
- Therapist makes judicious use of warmth, validation, humour and self-disclosure to try and make sessions enjoyable rather than aversive experiences. Therapists make active use of the therapeutic alliance, particularly focusing on and formulating things that have gone well. Therapists are ‘working towards the ending from the beginning’, gradually handing over responsibility for sessions to client over time and reinforcing independence, autonomy and self-efficacy to maximise the chances clients feel able to cope independently at the end of treatment.
- A major emphasis in ADepT is consolidating learning by encouraging clients to summarise sessions, complete session review sheets, put into practice learning, and feedback how this went. Clients are also given extensive handouts to read to ensure core concepts are covered (and so they can return to these in the future if needs be).
- ADepT follows a formulation driven approach rather than a precise therapeutic manual. One helpful metaphor is that being skilled at delivering ADepT is like being a concert pianist; you need to know the score well enough to then have confidence to improvise on top of it.
- The ADepT therapist self-rating tool and supervision rating tool are used to support therapist deliver the intervention with fidelity. These assess the development of competencies in three meta-domains: style, structure and techniques.

ADepT Rationale to Present to Clients

- The rationale presented to clients is about learning to life well with depression. ADepT sees depression as an obstacle towards achieving personal life goals that can be skilfully managed (rather than a state that needs to be eliminated/removed). This involves identifying what is important to life, working towards it, and anticipating and overcoming how their depression trips them up. If they can do this, it will maximise pleasure, meaning and social connection.
- The therapist and client work together to problem solve what helps and hinders achieving valued life goals – both in terms of taking opportunities (thriving) and managing challenges (resilience) in everyday life. Treatment will support the client to identify patterns of goal setting, thinking, feeling, behaving, and managing the body that they use; aiming to strengthen use of the adaptive patterns and to modify maladaptive patterns.
- The aim is to build a new ‘mental habit’ that moves clients from a negative outlook to a positive outlook. This may feel alien at first but with practice will become natural. At same time as doing this, ADepT will support the client to engage in good selfcare and wellbeing enhancing behaviours to recharge their batteries – people often forget to do this when depressed. Treatment will focus on helping client strike a balance between goal striving and self-nurturing.
- This rationale is adapted depending on clients’ chronicity (if first few episodes: this approach will help you get out of depression hole you are currently in; if a chronic, relapsing course: this approach will minimise frequency and intensity of future episodes and help you continue living well even when depressed). If useful, for more chronic clients introduce idea that wellbeing and illbeing are partly independent of each other and that it is possible to find wellbeing even in the midst of a depression episode.

Approximate Phases of ADepT

- Phase one is assessment and values clarification. It involves assessing depression; auditing current functioning in work, relationship, leisure, and self-care domains; identifying values, setting treatment goals to work towards; and identifying a first few (realistic) action steps to move towards these goals.
- Phase two is activation towards goals and formulation of mechanisms that help/hinder maximising opportunities and minimising challenges along the way. In early change sessions, there is no expectation of wellbeing or depression improvement and the emphasis is on understanding how depression trips individuals up; in later sessions clients should experience some gains as changes are made to depressogenic mechanisms. Key skills for the client to learn in early change sessions are behavioural activation and the capacity to formulate what helps/hinders this (using the COM-B framework and mapping tool). Clients are also encouraged to regularly practice self-care behaviours and to engage in wellbeing enhancing activities as a standard part of treatment. Clients also start to complete a positive journal (anticipating and remembering pockets of resilience/thriving). In later change sessions, the client and therapist test out new ways of coping to maximising resilience/thriving, using a mixture of psychoeducation, skills training, and behavioural experiments as appropriate. As necessary, therapy also works on coping with depression symptoms that snag activation (sleep, fatigue, pain, and cognitive dysfunction).
- Phase three is planning for ongoing activation after treatment finishes and developing a wellbeing plan to continue moving towards valued goals. Also, clients are encouraged to build self-care/wellbeing enhancing behaviours into routine life going forwards. Therapists anticipate possible snags with clients, and they plan together how these will be addressed. This phase is adapted depending on whether client has reached recovery or not (i.e., staying well going forwards or continuing journey to getting well). Wellbeing plans focuses both on moving towards ongoing goals and on relapse prevention/minimisation.
- Phase four is an extended follow up (up to five booster sessions over next year). Clients meet with therapists to discuss progress and to troubleshoot wellbeing plan. The focus of these review sessions depends on client’s mood state. If they have responded, emphasis is on continuing to do well (more like a coaching session). If they are still depressed, the emphasis is living as well as possible in context of depression (more like previous therapy sessions). These sessions are optional and can be scheduled flexibly to meet client needs.

ADepT Logic Model

Figure S1 outlines the logic model underpinning ADepT.

Figure S1

ADepT Logic Model

**
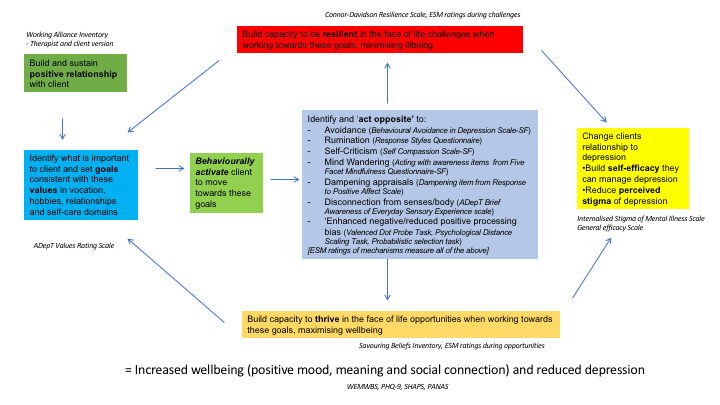
**

ADepT Session by Session Content

An overview of approximate content covered in ADepT in each session is provided in Table S1. ADepT is delivered in a tailored, flexible and formulation driven way, so this structure is often modified to align to client needs and the issues they wish to discuss in a given session. However, the expectation is this ground will all have been covered by the end of a complete therapy.

Table S1:

Session by Session Content in ADepT Intervention

| Session | Description |
| --- | --- |
| 1 | Assess the clients’ depression; review what is currently helping and not helping about how they are managing it; and introduce the ADepT rationale and structure. *Home activities:* Read treatment rationale handout, watch ‘BlackDog’ video about living with depression, complete mood diary |
| 2 | Review mood diary and reaction to rationale and video; identify values in vocational, relationship, self-care and leisure domains and introduce to ‘dartboard’ exercise. This involves rating how close to the ‘bullseye’ behaviour is to key values in each life domain. *Home activities:* read values handout, refine values, complete mood diary |
| 3 | Review mood diary and values homework; set values consistent goals in each life domain using extended ‘dartboard’ exercise. *Home activities:* Read goals handout, refine goals, complete mood diary |
| 4 | Review mood diary and goals handout; use a goal planning and monitoring tool to break goals down into SMART action steps and build the capability, opportunity and motivation to carry out each action step. *Home activities:* Read goal planning and monitoring tool handout and use to address one goal, read handout about overcoming snags that block goal pursuit. |
| 5 | Review use of goal planning and monitoring tool; introduce to mapping tool, which formulates mechanisms that help/hinder resilience/thriving. This tool can be used to map out an ‘old me’ (depressive coping) and to develop a ‘new me’ (constructive coping) in a situation. The ‘new me’ formulation will be utility based, focusing on what the goal is and then what would be a way of thinking and behaving that would be most likely to bring this about. *Home activities:* Use mapping tool to analyse one opportunity and one challenge in the next week. |
| 6 | Review use of mapping tool and introduce to positive diary keeping to capture moments of resilience and thriving. This intends to build a positive, specific memory and attentional style. *Home activities:* Read handout on positive diary, complete positive diary for next week. |
| 7 | Introduce to mindful engagement with everyday wellbeing activities that enhance pleasure, meaning and social connection. *Home activities:* Read handout on everyday wellbeing activities, practice mindful engagement with everyday activities and continue to complete action steps. |
| 8-12 | Use above tools to work through action steps identified above and develop new ways of coping when engaging with opportunities and challenges (‘acting opposite’ to depressive mechanisms). This will consist of psychoeducation around mechanisms, skills training around alternative ways of coping, and conducting behavioural experiments to test out and refine these new ways of coping. *Home activities:* Dependent on client goals and learning needs. |
| 13-15 | Develop wellbeing plan to continue to build wellbeing in months after therapy. This can include: reviewing goal progress and setting future goals on ‘dartboard’; reviewing key mechanisms helping/hindering resilience/thriving on formulation tool; reviewing key therapy techniques using a checklist; formulating a ‘relapse signature plan’ (early warning signs mood is dropping and steps that will be taken to minimise this); formulating a ‘wellbeing signature plan’ (early indicator signs mood is lifting and steps they will take to maximise this); sustaining engagement with everyday wellbeing activities and ‘positive review’. If useful, a carer/partner can be invited into later ADepT sessions to share learning and support the client with ongoing change after acute therapy has completed. *Home activities:* Read and complete wellbeing planning tools. |
| 15-20 | Five optional booster sessions will then be offered over the year after therapy. This will be used to review progress with goals, celebrate success, and trouble shoot any difficulties.  *Home activities:* Dependent on client goals and learning needs. |

Core competencies in ADepT delivery

Tables S2 provides an overview of the core style, structure and technical competencies therapists are trained to use, which can help identify similarities and differences of ADepT compared to other treatment approaches like CBT, BA, ACT, and positive CBT. These competencies also form the basis of the bespoke ADepT competency rating tool, used to assess to what extent therapists were competent, followed the treatment protocol with fidelity, and their practice was differentiated from CBT.

Table S2

ADepT Style, Structure and Technical Competencies

| **Style competencies** |
| --- |
| *Positive interpersonal style:* Display a positive, warm, humorous, reinforcing therapist interpersonal style and bring authentic therapist self into the room, to help client feel liked and to look forward to and enjoy sessions. This style is adapted to clients’ current mood state and capacity to focus on the positives, using ‘graded exposure’ to gradually increase positivity over sessions. |
| *Thickening the positive narrative:* Consolidate learning around moments of thriving and resilience the client displays whenever possible, making use of solution-focused questioning yet retaining a ‘positive-realist’ stance while doing so. |
| *Moving from the informal to the formal:* Introduce ideas and techniques informally through dialogue, before then consolidating through use of formal handouts and tools. |
| *Chunking:* Break tasks down into manageable action steps for the client, adapting for clients’ current functional capacity as appropriate. |
| *Working with the therapeutic alliance:* Build a positive working alliance with the client, working on positive and negative process enactments in therapy sessions as necessary to strengthen the relationship (including working towards endings). |
| *Changing relationship to depression:* Help clients to build a different relationship to depression, including externalising depression rather than seeing it as a core part of their identity, seeing depression as something that can be managed and lived well alongside, and reducing perceived shame and stigma around depression. |
| *Taking ownership of change:* Help clients notice progress and feel a realistic sense of ownership of change that occurs in and between sessions, building their sense of self-efficacy and agency. |
| *Tailoring:* Tailor protocol to fit client functioning, needs, and personal agenda as appropriate in each session. |
| **Structural competencies** |
| *Therapist preparation:* Prepare thoroughly for sessions and take notes afterwards, remembering key details about clients’ life, values and goals, and tracking ongoing agenda items across sessions. |
| *Agenda setting:* Collaboratively set and follow through an appropriate agenda in each session, ensuring this explicitly attends to moments of resilience and thriving in the clients’ life as well as working on difficulties and challenges. |
| *Utilising client feedback:* Seek, provide, and act on feedback from the client in each session, focusing on both what is going well and what are any things they would like to modify about the sessions. |
| *Homework:* Set and review homework in each session, reinforcing effort, troubleshooting difficulties, noticing successes, and linking this back to broader therapy goals. |
| *Consolidating learning*: Use memory techniques to help client consolidate learning in and between session, including regular summaries and reflections during session and emphasising key points from each session. |
| *Pacing:* Make effective use of session time and pace each session according to clients’ current functional capacity. |
| **Technical competencies** |
| *Psychoeducation:* Provide client with relevant psychoeducation to understand their depression and the ADepT rationale to overcoming it via building a positive focus (including sharing ADepT handouts and tools at relevant stage of therapy). |
| *Diary monitoring:* Support client to review current mood and functioning using ADepT diary, aiming to notice variability in mood, identify moments of resilience and thriving, label deprossogenic mechanisms to act opposite to, and to explore client values. |
| *Values clarification and goal setting:* Support client to identify realistic and values consistent goals across life domains, find appropriate balance between life domains, and break each goal into manageable action steps, using ADepT dartboard tool. |
| *Behavioural Activation:* Support client to activate towards goals, troubleshooting as necessary to overcome motivational barriers and making appropriate use of COM-B (capability, opportunity, motivation) framework and tool. |
| *Goal monitoring and values updating:* Support client to monitor progress towards goals and action steps, continuing to clarify and update client values based on outcomes of actions, using ADepT dartboard tool. This serves as the ‘macro formulation’ to guide overall treatment planning in ADepT. |
| *Formulating mechanisms around resilience and thriving:* Support client to understand and modify what helps/hinders resilience/thriving when engaging with activities/action steps, using ADepT mapping tool. Assist client to recognise and act opposite to common deprossogenic patterns that block thriving (including reduced positive attentional bias, reduced use of experiential processing, engaging in dampening appraisals, and not socially capitalising) and resilience (including heightened negative bias, withdrawal, rumination, self-criticism, and difficulties in making effective use of social support). Focus on contrast of ‘old/depressed’ patterns with ‘new/well’ patterns. Place an emphasis on establishing goals relating to each activity/action step and evaluating to what extent particular patterns help achieve these goals (a utility focus). This serves as the ‘micro formulation’ in ADepT to review how particular situations/actions went. |
| *Connecting to simple pleasures:* Support client to build an ongoing habit of engaging mindfully and experientially with everyday rewarding activities, using the ADepT simple pleasures tool. |

Table S2 (continued)

ADepT Style, Structure and Technical Competencies

| **Technical competencies (continued)** |
| --- |
| *Noticing better moments:* Support clients to build a habit of noticing and reflect on ongoing moments of resilience and thriving, through use of ADepT positive journal and informal day review techniques. Take a ‘graded exposure’ stance, aiming to gradually increase the degree to which client feels comfortable noticing better moments (and overcoming any anxiety, disgust or avoidance this positive focus may trigger). |
| *Enhancing self-care:* Support client to develop self-care habits (practical and emotional) and self-compassion and to overcome any barriers to doing so. |
| *Cognitive change:* Help client develop a range of skills to work with cognition. These include working with utility (identifying what patterns of attention/thought are most likely to achieve goals) and truth (evaluating available data to work out what is most accurate way to understand current situation) to help review events and plan for future events. Clients are also supported to notice unhelpful patterns of thoughts during activity, be able to decentre/defuse from these thoughts, and then as appropriate direct attention to the present moment, commit to valued activity, or rehearse thoughts that may be more helpful in the moment. Behavioural experiments are used to practice these skills (and guided reflection after these experiments are used to update the clients’ view of self, world and future). |
| *Emotional, interpersonal and practical change:* Make use of appropriate ADepT change techniques during both opportunities and challenges. This includes skills training, rehearsal, feedback, behavioural experimentation and reflection in: emotion-regulation techniques; interpersonal effectiveness techniques; and in practical problem solving techniques. Where clients have existing skills/strengths they are not utilising, the emphasis is on reorienting them to these skills/strengths and putting them into practice. |
| *Wellbeing planning:* Help client develop a wellbeing plan to sustain progress after the end of the acute therapy, including both ways to continue to work towards value consistent goals, build in wellbeing enhancing habits, and notice and respond skilfully to any ‘early warning signs’ of relapse. |

**3: Detailed Analysis Protocol**

3a: Clinical Proof of concept

Before proceeding to a large-scale definitive RCT evaluating ADepT, it is prudent first to optimise further the ADepT protocol, resolve any uncertainties about the planned RCT design, and to establish stronger proof of concept about the possible clinical effectiveness of ADepT. A pilot RCT – a smaller scale ‘mock up’ of the planned subsequent definitive trial – is an efficient way to achieve these aims^1^. There is ongoing debate about the validity of conducting proof-of-concept analyses on pilot trials^2,3^, and some argue it is not valid to infer proof of concept from pilot studies at all^3^. In our view, the within-arm frequentist analyses, examination of confidence intervals from between-group analyses, and inspection of between-group Bayesian credibility intervals conducted below provide an appropriate way to estimate ‘signal’ from the present pilot trial (maximising value of information gained and minimising research waste by ensuring ADepT is only taken forward to definitive trial if results look promising).

Data Handling Procedures

All questionnaire measures were checked for missing values after completion and where possible participants were approached to answer the missing items. Data were entered into a database by trial researchers. The two primary outcomes captured at each assessment point (PHQ-9 and WEMWBS) were double entered and checked for errors. A random subset of other measures was also checked for errors. Where less than 10% of items on a questionnaire measure was missing, a corrected sum scale score was computed (using the average across the completed items to estimate the value of any missing items). Any other errors that came to light during the analysis were corrected. Data were initially entered and checked in the Statistical Package for Social Sciences (SPSS)^4^. Subsequent proof-of-concept analyses were implemented in the statistical package R^5^. Analyses were conducted by the trial principal investigator (BD) and were checked by trial research fellow (LW), seeking input from statisticians at the Exeter CTU and the trial health economist (AS) as required to support analyses.

Within-Arm Analyses

Paired sample t-tests were run to examine within-arm effect sizes for change in each continuous clinical outcome from intake to six-month assessment for each group separately. These analyses were run on an intent-to-treat (ITT) basis, with missing data being simulated via multiple imputation using the R MICE package^6^, assuming that data was missing at random. Cohen’s d (and associated 95% confidence intervals) were reported as measures of effect size. These within-arm analyses do not control for non-specific factors like expectancy effects and also it is possible any changes observe reflect spontaneous recovery over time, so are only weak evidence of efficacy. Moreover, it is not appropriate to compare the magnitude of within-arm effects in each arm to infer anything about likely between-group differences^7^ . However, such analyses are useful to help inform continuation to definitive trial. An intensive therapy like ADepT is only likely to be worth evaluating further if it produces within-arm improvements over time that bench with large magnitude effect size pre- to post- outcomes seen in routine clinical care. Moreover, it is also important to be able to establish if CBT was delivered competently in the pilot trial, or if changes need to be made to CBT therapist selection, or therapist training and supervision. Inspection of within-arm changes in the ADepT and CBT arms help make these judgments.

Between-Arm Continuous Analyses

Linear regression analyses were run to estimate between-group effect sizes (and their 95% confidence intervals). In each regression, scores on the outcome at the six-month assessment was the dependent variable, treatment arm was the predictor variable and the trial stratification variables and intake scores on the independent variable were entered as covariates. Non-parametric bootstrapping methods were used to estimate the adjusted group term confidence interval in these analyses, as this approach gives a more accurate forecast than conventional parametric methods in small sample, under-powered analyses. Multiple imputation was nested within each bootstrap to simulate missing data using the R package bootimpute^8^, assuming data was missing at random. Effect sizes estimates (and their 95% confidence intervals) were expressed in raw score units and were also converted into standardized units (Cohen’s d) by dividing them by the standard deviation of the outcome at six months. Established thresholds for minimum clinically important difference (MCID) were available for the PHQ-9 (1·7 points^9^), GAD-7 (1·15 points^9^), WEMWBS (2·8 points^10^), and HDRS (3·0 points^11^). Visual analyses examined whether the lower bound of the confidence interval crossed into the range where ADepT might be MCID inferior to CBT (raw score deterioration >MCID value) and whether the upper bound of the confidence interval crossed into the range where ADepT might be MCID superior to CBT (raw score improvement > MCID value) for each of these outcomes. It has also previously been estimated that a Cohen’s d value of + 0·24 can be used as a proxy estimate of MCID for continuous depression outcomes^12^. Visual analyses examined whether the lower bound of the confidence interval crossed into the range where ADepT might be MCID inferior to CBT (d<-.24) and whether the upper bound of the confidence interval crossed into the range where ADepT might be MCID superior to CBT (d>.24) for all continuous outcomes. In the current sample, at six months a d of 0·24 equated to a 1·4-point change on the PHQ-9 and a 2·4-point change on the WEMWBS. This aligns reasonably with existing individual based MCID estimates outlined above (>1·7 points on PHQ-9^9^; >2·8 points on WEMWBS^10^).

To index cumulative change in symptoms and positive functioning more effectively over the follow-up period, we modified the primary follow-up analyses specified in the protocol paper^13^. Rather than looking separately at twelve-month and eighteen-month follow-ups, we instead computed area under the curve (AUC) across the trial follow-up period to index cumulative symptoms/positive functioning. Inspection of the data revealed non-linear trends across time that varied between individuals and for some involved patterns of improvement and worsening over time. AUC methods can accommodate different patterns of change over time between individuals and do not impose restrictive assumptions on the nature of this pattern, so are well suited for modelling data of this kind.

AUC for each variable was computed using the trapezoid rule (summing the approximations of AUC for the intake to six-month assessment, the six-month to twelve-month assessment, and the twelve-month to eighteen-month assessment). Where outcome data were missing for a single follow-up assessment at six months or twelve months, values either side of it were averaged to approximate values at that time point, and this approximated value was used in the AUC calculation. Where only eighteen-month data were missing, we used the change observed from six months to twelve months to estimate the eighteen-month value (assuming a linear trend). No replacement was made of missing intake values. These AUC estimates were then entered as the dependent variable in a series of continuous regression analyses (with the same predictors and covariates as in the six-month continuous analyses) and were interpreted relative to the d=0·24 threshold used in the six-month analyses. For the sake of completeness, analyses were also conducted at twelve months and eighteen months as specified in the original protocol paper^13^ (see SOM section 3).

Between-Arm Categorical Analyses

Between-group binary clinical outcome analyses were also conducted at each follow-up period. The following metrics were pre-specified in the protocol paper^13^: reliable and clinically significant improvement on all outcomes at all time points; response (>50% improvement) and remission (falling beneath clinical cut-offs) on the PHQ-9, and HDRS at six months; remission on the SCID at six months; and IAPT reliable improvement, recovery and reliable recovery at six months. Reliable improvement cut-offs were based on the standard deviation observed in the current sample at intake and estimates of scale internal reliability from the extant literature, following the approached outlined by Jacobson and Truax^14^. Clinically significant criteria used established external cut offs where these were available and if not criterion c^14^. We used established clinical cut-offs to assess remission on the PHQ-9, HDRS, and WEMWBS. The PHQ-9 and HDRS both have a zero point that represents an absence of symptoms, so computation of response is simply whether participants have moved at least 50% closer to zero during treatment. The WEMWBS does not have a clear zero point, so instead we defined response as moving 50% closer to general population average values during treatment. IAPT reliable improvement means showing reliable improvement on either the GAD-7 (>4 point change) *or* the PHQ-9 (>6 point change). Recovery means scoring in the non-clinical for PHQ-9 (<10) *and* GAD-7 (<8).

We conducted the following additional categorical post hoc analyses at six months. As WEMWBS was a co-primary outcome, we computed response and remission on the WEMWBS also. To have single composite metrics of improvement across the two co-primary outcomes in the trial, we computed rates of simultaneously meeting remission criteria for the PHQ-9 and WEMWBS and simultaneously meeting response criteria for the PHQ-9 and WEMWBS. To allow benchmarking to the extant literature on how well depression treatments repair positive and negative valence system outcomes, we also computed rates of remission and response on the SHAPS (anhedonia), the PANAS (positive and negative affect), and the MASQ-S30 (anhedonia and general distress) scales. For the PANAS and MASQ-S30, we defined remission as falling within 0.5 SD of general population averages^15^. For the SHAPS, we defined remission as scoring less than 25 on the SHAPS^16^. The PANAS, SHAPS, and MASQ-S30 scales do not have a clear zero point, so instead we defined response as moving 50% closer to general population average values during treatment.

All binary outcomes were analysed using intent-to-treat logistic regression models, with the same predictor variables and covariates as previously described in continuous analyses. As a number of models would not converge when using the bootimpute package^8^, instead missing data were simulated using the MICE package^6^. The effect sizes reported are odds ratios and their associated 95% (parametric) confidence intervals.

Benchmarking Analyses

Benchmarking analyses were conducted to examine to what extent the effects of treatment in the CBT and ADepT arms of the pilot trial were comparable to those observed in the extant literature. We benchmarked against two RCTs conducted in similar UK primary care depressed samples. The COBALT trial^17^ recruited 469 treatment-resistant currently depressed clients (defined as not responding to anti-depressant medication given for at least six weeks at an adequate dose). These participants were randomized to receive either up to eighteen sessions of CBT as an adjunct to continuation medication or treatment as usual (TAU; continuation medication). CBT was superior to TAU, supporting the effectiveness of CBT with this client group. The COBRA trial^8^ recruited 440 currently individuals with depression (with no inclusion criteria around treatment resistance or prior medication use) and randomized them to receive up to twenty sessions of either CBT delivered by high intensity therapists or BA delivered by low intensity therapists. CBT and BA were both equivalently effective at treating depression, but BA was health-economically dominant. COBRA and COBALT both captured PHQ-9 and GAD-7 outcomes (and COBRA additionally captured SHAPS anhedonia) at intake and six-month assessment.

We computed within-arm effect sizes at the six-month outcome point (Cohen’s d and associated 95% confidence intervals from complete case analyses) in each arm in each of the COBRA and COBALT trials and compared these to complete case estimates from the ADepT trial. We also ran post hoc complete case regression analyses comparing the effects in each arm of the ADepT trial relative to each comparator arm in each of the other trials. These analyses were identical to those comparing the ADepT arms (predicting six-month raw score differences on each outcome as a function of group, covarying for intake depression severity [moderate or severe] and anti-depressant medication status [taking medication, not taking medication]). We report the raw score difference between arms (and the associated 95% confidence interval).

We also benchmarked depression remission and response rates to meta-analytic findings of depression psychotherapy trials. These analyses should be interpreted cautiously, as groups were not randomly allocated and likely differ in intake demographic and clinical characteristics. Nevertheless, they allow provisional benchmarking of effect sizes in the current trial CBT and ADepT arms relative to previous trials.

Survival Analyses

Survival analyses were run using Cox’s regression proportional hazards model on the subset of participants who met remission criteria on the SCID at the six-month assessment, examining if there was a differential risk of relapse until the eighteen-month follow-up as a function of treatment allocation. The key predictor variable was treatment arm, and the trial stratification variables (moderate or severe intake depression severity; whether or not anti-depressant medication was being taken at randomisation) were entered as covariates. The analysis included all eligible patients and censored for missing data over the follow-up period. We assessed the proportional hazards assumption using the cox.zph command in R. As survival analysis handles missing follow-up data through censoring, no imputation procedures were used to simulate missing data. All of the individuals included in the analyses had completed a minimum adequate dose of therapy, making this approximate to a per-protocol analysis. The hazard ratio (and the 95% confidence interval) of the group term from this analysis were reported. The trial protocol paper also stated we would analyse survival at twelve-month follow-up, so this is reported in SOM Section 4. In both the twelve-month and eighteen-month analyses there was no evidence that the proportional hazards assumption was violated (χ^2^  Ps>0·48 for all terms in models).

Bayesian Analyses

Bayesian analyses (on complete case data) were conducted to generate 95% credibility intervals of the difference between ADepT and CBT at six-month, twelve-month, and eighteen-month assessment on the two primary outcomes of PHQ-9 depression and WEMWBS wellbeing. The analysis models (adjusting for the trial stratification variables and intake symptom severity on the dependent variable) were rerun using the Bayesian modelling package rstanarm v 2.21.1^19^ on complete case data. The default (weakly informative) priors were applied (error standard deviation set as exponential; co-efficient and intercepts set as normal, standardized and with an SD of 2.5) to provide some regularization to avoid overfitting. The default settings for Markov Chain Monte Carlo (MCMC) simulations were also used (four chains, each with 2000 iterations and a burn in phase of 1000 iterations, leading to 4000 iterations in the final simulation). Model convergence was adequate for all models (for all parameters: Rhat<1.1; effective sample size > 10% of target sample size; Monte Carlo standard error < 10% of posterior standard deviation; trace plots were noisy and overlapping across chains; and autocorrelation plots tended to zero). Graphical posterior predictive checks (using the pp_check function in rstanarm) were also all plausible. Using output from these models, a series of plots visualised the expected additional change in raw score outcomes for the 1^st^ to the 99^th^ centiles of clients when treated with ADepT rather than CBT at each assessment point, relative to estimates of MCID on each scale (including the 95% credibility interval for each scale). Lower centiles represent a ‘worst case scenario’ and higher centiles represent a ‘best case scenario’ of the benefits of ADepT relative to CBT on each outcome. MCID was estimated as a 1·7 point difference on the PHQ-9, based on the amount of change over a two-week period that individuals with moderate depression severity reported as meaning they felt at least slightly better^5^. MCID was estimated as a 2·8 point difference on the WEMWBS, based on averaging across five computations of one standard error of measurement (SEM) on the scale across different studies reported by Maheswaran and colleagues^10^. We report the percentage of clients who would show a MCID advantage of ADepT over CBT and vice versa. This approach is an extension of the ACCEPT methodology recently proposed to help interpret trial data^20^. The six-month analyses are reported in the main paper and the twelve-month and eighteen-month analyses are reported in SOM Section 3. We have not conducted these analyses on the eighteen-month AUC data, as MCID estimates are not interpretable in this context.

Secondary Sensitivity Analyses Examining Therapist Clustering Effects

Secondary sensitivity analyses examined if there was any clear evidence of therapist clustering effects. We repeated the primary outcome clinical comparisons using a mixed model analyses on complete case data, examining if treatment arm predicted differences in six-month outcomes. For all mixed models, a restricted estimate maximum likelihood (REML) approach was used, with the Satterthwaite approximation applied to estimate denominator degrees of freedom. Each model used the same dependent variables, predictor variables and covariates as described in the original linear regression analyses, except for the addition of the therapist variable. All effects in models were specified as fixed, apart from therapist being specified as a random effect. Models were run on complete case data, excluding the two participants who did not start treatment (and so were not assigned a therapist). The original intention was to fit a random intercept and slopes model, using an unstructured covariance structure. These models would not converge, so instead a random intercept only model was used. As this left only a single variable random effects specification, the covariance structure was then reset as identity. Analyses report the intraclass correlation (ICC) and the results of the log restricted likelihood test (LR) to assess if there was evidence of clustering. The between group effect (and associated 95% confidence interval) for the mixed model and a linear regression on the same data are reported, to allow inspection of the impact of accounting for therapist clustering on the findings observed.

For the PHQ-9 analyses, the ICC was negligible (2.51e-23) and the LR test non-significant (P=1.00). There was little difference between the mixed model group estimate ( -1.46, 95% CI= -4.76, 1.76) and the linear regression estimate (-1.46, 95% CI= -4.00, 1.09). For the WEMWBS analyses, the ICC was negligible (9.44e-16) and the LR test non-significant (P=1.00). There was little difference between the mixed model group estimate (3.54, 95% CI= -2.34, 9.42) and the linear regression estimate (3.06, 95% CI= -1.62, 7.75). For the EQ analyses, the ICC was negligible (1.49e-19) and the LR test non-significant (P=1.00). There was little difference between the mixed model group estimate (0.05, 95% CI= -0.09, 0.19) and the linear regression estimate (0.05, 95% CI= -0.05, 0.16). For the ICECAP analyses, the ICC was negligible (8.28e-24) and the LR test non-significant (P=1.00). There was little difference between the mixed model group estimate (0.10, 95% CI= -0.02, 0.23) and the linear regression estimate (0.10, 95% CI= 0.01, 0.21). For the cost analyses, the ICC was small (.013) and the LR test non-significant (P=0.44). There was little difference between the mixed model group estimate (-£40, 95% CI= -£618, £538) and the linear regression estimate (-£40, 95% CI= -£817, £735). Given the small number of clusters and the small and variable number of samples within each cluster, the reliability of these multilevel modelling results is not high^21^ but nevertheless does not indicate clustering by therapist is markedly biasing the current results. We therefore report the analyses uncorrected for clustering as originally specified in the trial protocol paper^13^.

3b. Health Economic Proof of Concept

Before proceeding to a large-scale definitive RCT evaluating ADepT, it is also sensible to estimate potential cost and cost-effectiveness of the approach to see if it would be viable for decision-makers to implement if it is found to be clinically effective. Therefore, an exploratory health economic analysis was conducted. The cost-effectiveness analyses were conducted post hoc, and no health economic analysis protocol was pre-registered. Therefore, the description presented serves as the most complete description of how analyses were conducted. Health economic descriptive analyses were conducted using SPSS^4^ and cost-effectiveness analyses were conducted using R^5^.

Aim, Perspective and Time Horizon

The aim of the economic evaluation was to preliminarily assess the cost-effectiveness of ADepT compared with CBT in the treatment of depression, to help inform continuation to definitive trial. As recommended by the UK National Institute of Clinical Excellence (NICE), the economic evaluation took a NHS/personal social services (PSS) perspective, covering the use of hospital, community, health and social services. The analysis was within trial (using an eighteen-month time horizon) and no modelling was used to extrapolate findings beyond the final follow-up.

Outcomes

The EQ-5D-5L was used as a health-related utility measure and the ICECAP was used as a wellbeing-related utility measure. Health and wellbeing states were converted into a single summary index utility score for each measures by applying weights to each level in each dimension derived from the valuation of EQ-5D health states and ICECAP wellbeing states in UK adult general population samples^22,23^. Given that the NICE and other decision-makers currently recommend use of the three-level rather than five-level version of the EQ-5D, a validated cross mapping tool was used to convert answers on the five-level scale administered in the trial to utility scores based on the three -level version^24^. Future research should directly administer the three-level version of the EQ-5D rather relying on this cross-mapping tool. Quality Adjusted Life Years (QALYs) were calculated as the area under the curve (AUC) defined by the utility values at baseline and each follow-up, making the assumption that utility score changes over time were linear in nature. As with the eighteen-month AUC clinical outcome analyses, AUC for each variable was computed using the trapezoid rule. Where EQ-5D or ICECAP data were missing for a single follow-up assessment at six months or twelve months, values either side of it were averaged to approximate cost data for that time point. Where only eighteen-month data were missing, we used the change observed from six months to twelve months to estimate the eighteen-month value (assuming a linear trend). No replacement was made of missing intake values. A discounting factor of 3.5% per year for QALYs was used.

Calculation of Costs

Cost of CBT and ADepT treatment (in UK £) was calculated using a micro-costing (bottom-up) approach^25^. Staff therapist costs were based on the NHS Agenda for Change salary band 7 and staff supervisor costs on the NHS Agenda for Change salary band 8a for 2018-2019 (£54 and £65 per hour respectively, including national insurance and pension contributions plus capital, administrative and managerial overheads). Cost per hour was calculated, estimating a fixed amount of time for training and supervision per client and then allowing 1.5 hours for each session delivered and 0.5 hours for each session cancelled or missed. Initial training costs (one day for each of four therapists and two trainers per arm) and ongoing supervision costs (assuming ninety-minute supervision and fifteen-minute preparation time for each week patients were being treated in the two-year acute phase, with four therapists being supervised in group format by one supervisor in each arm) were computed. In the ADepT arm only, booster session supervision costs were estimated assuming fortnightly supervision of thirty minutes for each week patients were being treated in the one-year booster phase, again with four therapists being supervised in group format by one supervisor. This worked out at a total training/supervision cost for the ADepT arm of £48051 (£1172 per patient) and in the CBT arm of £45241 (£1103 per patient).

Broader service use was measured via the AD-SUS interview, which asks participants for the number and duration of contacts with the range of services and professionals (and also use of medications). The AD-SUS explored the following hospital use service use categories: hospital admission, hospital outpatient appointments, accident and emergency attendances (and if these required an ambulance). The AD-SUS also covered the following community-based health and social and complementary services: GP surgery; GP home visit; GP telephone; practice nurse; district nurse, health visitor or midwife; community psychiatric nurse; psychiatrist in the community; occupational therapist in the community; art/drama/music therapy in the community; social worker; marriage counselling; advice service (e.g., citizens advice bureau); helpline (e.g., Samaritans/MIND); day centre or drop-in centre; chiropractor/osteopath; homeopathy; acupuncture. Participants could also specify other service use categories not covered above. An additional hospital service use category that was collected but not included in costings was diagnostic tests (as this was judged to not be relevant to mental health and would likely significantly skew cost estimates). The AD-SUS also collected detailed use of medications. At intake, the interview covered the six months before entering the trial. At each of the subsequent assessments, service use since the previous interview was recorded to ensure that the entire period from intake to eighteen-month assessment was covered. A unit cost (in UK£) was applied to each resource use to calculate the total costs of resources used by each individual in the trial. Unit costs were taken from 2018-2019 rates from the Personal Social Services Research Unit [PSSRU]^26^ and/or NHS Reference costs^27^; see Table S3). Medication costs were calculated using daily dose information and the cost of generic drugs detailed in the British National Formulary (BNF)^28^. Where AD-SUS data were missing for a single follow-up assessment, this value was substituted using the average across other assessment points. AD-SUS costs and direct treatment costs over the trial follow-up period (six months, twelve months, and eighteen months) were summed together to create an overall cost for each participant in each treatment arm. A discounting factor of 3.5% per year for costs was used.

Data Handling

EQ-5D-5L, ICECAP, AD-SUS and session attendance data for each participant at each assessment were entered into SPSS and checked for missing values (missing values coded as 999) by a research assistant working on the project. A random subset of data was checked for accuracy by the trial principal investigator. Extreme high or low values were double checked to ensure there had not been a data entry error. The data were then transferred into R, where scripts were run to cost intervention use and convert EQ-5D and ICECAP scores into QALYs.

Table S3

Unit costs applied to economic data.

| Service | Unit | Cost |
| --- | --- | --- |
| Medication | Per daily dose | Various |
| *AD-SUS Hospital* |  |  |
| Inpatient stays | Per night | £727 |
| Outpatient appointments | Per contact | £137 |
| Accident and emergency | Per contact | £148 |
| Ambulance | Per contact | £252 |
| *AD-SUS community (listed)* |  |  |
| GP surgery | Per contact | £28 |
| GP home | Per contact | £35 |
| GP telephone | Per contact | £21 |
| Practice nurse | Per contact | £11 |
| District nurse, health visitor, midwife | Per contact | £65 |
| Community psychiatric nurse | Per contact | £64 |
| Community psychiatrist | Per contact | £341 |
| Occupational therapist in community | Per contact | £28 |
| Art/drama/music therapist | Per contact | £53 |
| Social worker | Per contact | £29 |
| Marriage counselling (e.g. Relate) | Per contact | £49 |
| Advice service (e.g. Citizens Advice Bureau) | Per contact | £31 |
| Helpline (e.g. Samaritans) | Per contact | £4 |
| Day centre/drop in centre | Per contact | £32 |
| Chiropodist/osteopath | Per contact | £55 |
| Homeopathy | Per contact | £61 |
| Acupuncture | Per contact | £61 |
| *AD-SUS community (other)* |  |  |
| Mental Health Assessment Team | Per contact | £35 |
| Crisis Team | Per contact | £43 |
| Clinical Psychologist | Per contact | £53 |
| Senior Mental Health Practitioner | Per contact | £44 |
| Primary care counselling | Per contact | £43 |
| Private therapy | Per contact | £34 |
| Hospital drop in mindfulness session | Per contact | £15 |
| Group MBCT course | Per 8-week course | £105 |
| Hypnotherapy | Per contact | £50 |
| Massage therapy | Per contact | £67 |
| Support worker | Per contact | £20 |
| Physiotherapy | Per contact | £53 |
| Podiatrist/chiropodist | Per contact | £53 |
| NHS 111 call | Per contact | £8.50 |
| Access to work scheme | Per contact | £16 |
| Workplace Occupational Health | Per contact | £43 |
| Workplace employee assistance programme | Per employee | £14 |

Health Economic Data Analysis

Resource category use by participant was reported as mean by intervention group and the percentage of each group who at had least one contact with each resource category. The mean (SD) QALY, intervention costs, broader health care utilization costs, and total costs for each treatment arm were reported. Cost estimates of each treatment were reported.

Differences between randomised group in mean total costs, mean EQ-5D QALYs, and mean ICECAP QALYS across the eighteen-month follow-up were analysed using linear regression, each covarying for trial stratification variables, intake EQ‑5D and ICECAP utility scores, and (log transformed) resource utilization in the six months prior to the trial. The estimates of between-group differences (and their 95% confidence intervals) from these analyses were reported. As with the clinical proof of concept analyses, nonparametric bootstrapping methods were used to estimate the adjusted group term confidence interval in these analyses. Multiple imputation was nested within each bootstrap to simulate missing data using the R package bootimpute^8^, assuming data was missing at random.

The primary economic analyses focused on cost-effectiveness of ADepT relative to CBT in terms of cost per QALY, conducting separate analyses for EQ-5D and ICECAP QALY estimates. To model statistical uncertainty, repeat resampling (bootstrapping) from complete pairs of cost and QALY data were used to generate a distribution of mean costs and QALY outcomes. It was not possible to embed multiple imputation within these paired bootstrap estimates, so these analyses used the subset of participants with complete cost and QALY data (32/41 CBT; 32/41 ADepT). Bootstrapping was implemented in the R package boot v1.3-28, with 1000 replications and using the ordinary (non-parametric) simulation method^29^. These distributions were used to calculate the probability that ADepT or CBT is the optimal choice, subject to a range of maximum values a decision-maker might be willing to pay for a unit improvement in outcome (willingness to pay thresholds). As recommended by NICE, £20000 per QALY was used as the base case willingness to pay threshold. To investigate the uncertainty that exists around estimates of costs and effects, the bootstrap replications were fed in as vectors to the R package Bayesian Cost Effective Analysis (BCEA) version 2.4.1, which was then used to generate a series of plots to help guide a probabilistic sensitivity analysis^30^. Cost-effectiveness plane figures are scatterplots of each bootstrap pair of cost and outcome estimates (with the red dot representing the pooled average across bootstraps), fitting a £20000 willingness to pay line. Expected incremental benefit (EIB) plots show the expected incremental benefit of ADepT relative to CBT for different willingness to pay thresholds, including 95% credibility intervals of these estimates. Cost-effectiveness acceptability curve (CEAC) plots show the probability ADepT is cost-effective relative to CBT for different willingness to pay thresholds. The 95% confidence interval of these estimates is reflected by the range between 2.5% and 97.5% probability of cost-effectiveness on these plots: if estimates fall above/below this range this would indicate ADepT is clearly superior/inferior to CBT using conventional frequentist criteria.

A secondary sensitivity analysis explored the cost-effectiveness of ADepT relative to CBT at the six-month assessment (the primary clinical end point). Reflecting this is a preliminary analysis of a pilot trial to help inform continuation to a definitive trial, rather than a definitive cost-effectiveness evaluation, no further secondary sensitivity analyses were run. In a subsequent definitive trial, secondary sensitivity analyses should include examining if imputing missing data, adjusting perspective, or modelling beyond the end of the trial follow-up changes the pattern of findings. For similar reasons, no subgroup analyses were run, no efforts were made to characterize distributional effects across individuals, and no adjustments were made to reflect priority populations in the current analyses. While public and patient involvement helped shape the design of the ADepT intervention and the trial design more generally, they provided no specific input to the health economics aspects of the study.

3c. Changes to Trial Design and Analysis

History of Significant Amendments Made to Approved NRES Ethics Protocol

- Prior to the trial being registered on ISRCTN and opening for recruitment, we stepped back from the original aim stated in the grant application of conducting surrogate between-group analyses on PANAS positive affect (including reporting p values) to establish proof of concept. After exploring MCID estimates on the PANAS in more detail, we realised the trial was not adequately powered for this purpose and we did not have resources to increase sample size. This change was approved by statisticians in the local clinical trials unit, the funder, and the ethics committee.
- We modified inclusion criteria four months after opening for recruitment to be clear that there was no upper age limit on who was eligible for the study (some documents had originally incorrectly stated an 18-65 rather than an 18+ age range). This change was approved by the ethics committee.

Clarifying Minor Differences in the ISRCTN Registration and the Trial Protocol Paper

- The trial ISRCTN registration (85278228) for the present study listed as primary outcomes the range of feasibility aims of the pilot trial to inform continuation to definitive trial. The trial protocol paper^13^ continued to list the continuation rules that would be tested to inform continuation to definitive trial but specified as primary outcomes the candidate measures that would be used to evaluate clinical effectiveness in a definitive trial (changes in PHQ-9 depression and WEMWBS wellbeing at six-month assessment). In the present manuscript, we follow the approach outlined in the trial protocol paper.

Minor Changes from Analysis Protocol Specified in Trial Protocol Paper^13^

- We do not report inferential results (p-values) from within-arm paired sample t-tests and only focus on the confidence interval of the effect size, as this change to our pre-specified analysis protocol was mandated by one of the Reviewers of this article.
- The secondary sensitivity analyses examining potential therapist clustering effects were not a priori specified in the original protocol. They were conducted post hoc at the request of one of the Reviewers of the manuscript.
- We stated in the trial protocol paper we would conduct Bayesian analyses of between-group effects. The AccEPT curve methodology we use to visualize this analysis had not been published at the time we wrote the protocol, so reflects a post hoc extension of this planned analysis.
- We stated in the trial protocol paper we would report between group confidence intervals to evaluate potential proof of concept of ADepT. The decision to interpret these confidence intervals relative to thresholds for minimum clinically important difference reflects a post hoc extension of this planned analysis.
- We stated in the trial protocol paper we would benchmark the trial findings to the extent depression trials literature. A direct comparison of findings to the COBRA and COBALT trials was a post hoc extension of this planned analysis.
- We report effect size units in terms of Cohen’s d (and associated 95% confidence intervals) for regression analyses rather than **η2** (and associated 90% confidence intervals), as these are more intuitively interpretable relative to estimates of d=0·24 representing MCID for depression outcomes^12^.
- We examined long term outcomes via computing area under the curve (AUC) over the entire follow-up period as our main follow-up analysis (see section above for justification). However, we do still report separate analyses for twelve-month and eighteen-month follow-up as specified in the original protocol paper (see SOM Section 3)
- We were not able to reliably estimate MCID from participants’ global ratings of change scales due to a small sample size with high variability, so reliable and clinically significant change analyses used the threshold for clinically significant change based on external or statistical criteria.
- Treatment satisfaction ratings were only taken once at some point after acute treatment finished (and not twice as specified in the protocol paper).
- Some clinical proof-of-concept analyses were post hoc and so were not specified in the original trial protocol paper: i) creating response and remission variables for a wider range of variables; ii) creating composite remission and response variables pooling across the PHQ-9 and WEMWBS; and iii) formal comparison of ADepT trial outcomes relative to COBRA and COBALT trial outcomes.
- The decision to conduct a more complete cost-effectiveness analysis, including generating cost-effectiveness plane, EIB, and CEAC plots, was made post hoc and was not specified in the original trial protocol paper.
- We had originally planned to pilot use of a pen and paper resource log to see if this facilitated health economic data collection (randomizing half of participants to receive the log and half not to receive the log). Due to experimenter error, this did not happen (and no participants were given the resource log).
- The protocol paper stated that: moderation analyses; mixed models analyses examining weekly change in symptoms during acute treatment; a mixed method process evaluation; and a health economic decision tree modelling analysis would be conducted. These will be reported elsewhere.

**4: Detailed Feasibility Analyses**

Continuation Rule One: Recruitment

*Criterion: > 60% of target sample (of 80) can be recruited*

To examine recruitment, the number (%) of participants recruited in each arm, recruitment rates each month, and the efficiency (conversion rates) of each recruitment method were reported. Conversion rates were computed in terms of the percentage of clients initially approached, who expressed interest, and who were assessed that were eventually randomised. Eighty-two participants were recruited (103% of the target sample size; > 60% continuation rule) over fifteen months, at an average rate of 5.5 participants per month and with recruitment tracking intended rates over time (Table S4 and Figure S2). The primary recruitment method via IAPT record search worked efficiently, accounting for 60/82 participants (73% of the sample). There was an adequate supply of potentially eligible clients on IAPT waiting lists (record searches identified 607 suitable clients over the course of the recruitment window). To avoid over-recruitment, only a subset of these eligible individuals was written to. Each month, the number of potential clients that could be taken on by the therapy teams was calculated and permission to contact letters were sent out to roughly three times that number of clients (anticipating around a 33% conversion rate). Conversion rates from approach, expression of interest and assessment were 38%, 69% and 88% respectively. Other recruitment methods contributed a relatively small number of clients, with marked variability in their efficiency.

Table S4

Recruitment flows and efficiency for each recruitment source.

|  | IAPT record search | Counselling record search | GP record search | AccEPT clinic referral | Self-referral |
| --- | --- | --- | --- | --- | --- |
| Identified | 607 | - | - | - | - |
| Approached | 160 | 20 | 31 | - | - |
| Expressed Interest | 87 | 8 | 7 | 14 | 22 |
| Assessed | 68 | 2 | 2 | 12 | 15 |
| Randomized | 60 | 1 | 1 | 11 | 9 |
| Treatment Allocation | 32/28 | 0/1 | 1/0 | 5/6 | 3/6 |
| % of overall sample | 73 | 1 | 1 | 13 | 11 |
| Conversion from approach | 38% | 5% | 3% | - | - |
| Conversion from expressed interest | 69% | 13% | 14% | 79% | 41% |
| Conversion from assessment | 88% | 50% | 50% | 92% | 60% |

*Note –* Data are count or percentage values; treatment allocation is number allocated to CBT/ADepT.

Figure S2

Recruitment over time, relative to target recruitment schedule


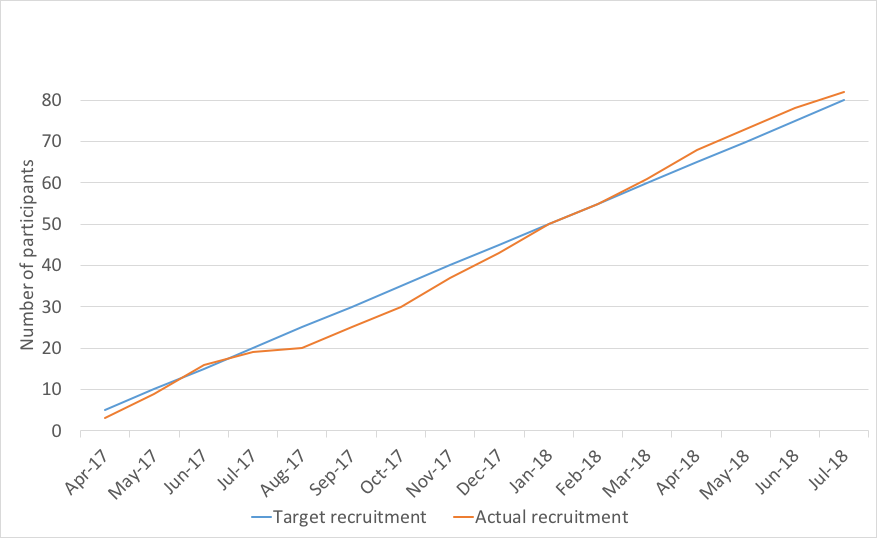


Continuation Rule Two: Data Completeness

*Criterion: >60% of sample recruited return primary outcome data at each assessment point*

To evaluate data completeness, the number (%) of participants in each arm completing each assessment and providing outcome data on the two primary outcome measures (PHQ-9 and WEMWBS) at each assessment was reported (Table S5). Attendance rates were adequate in both arms at the primary six-month assessment (>85%) and at each subsequent follow-up appointment (>70%). Completion rates of the primary outcome measures were also satisfactory in both arms at six-month assessment (>83%) and at all subsequent follow-ups (>61%), exceeding the continuation target of 60%. That completion rates for the primary outcome measures were marginally lower than rates of attendance at the assessments reflects the fact that some participants took the (anonymized) self-report questionnaire booklet home to complete after the interview assessment in their own time and then this was not returned. Data completion rates for the secondary outcomes were almost identical to the primary outcome measures, as they formed part of the same assessment questionnaire pack (Table S6). There were minor discrepancies in completion rates where clients missed out items on a questionnaire (precluding scoring) or where they failed to complete page in the assessment booklet. Due to researcher error, the PANAS was not administered to some participants at eighteen months, leading to a relatively greater proportion of missing data (23/41 [56%] CBT and 24/41[585) ADepT complete data) relative to other measures.

Table S5

Attendance at assessments and primary outcome data completeness rates at each assessment.

| Assessment | Arm | Attended  assessment | WEMWBS and PHQ-9 available |
| --- | --- | --- | --- |
| Intake | CBT | 41 (100%) | 41 (100%) |
|  | ADepT | 41 (100%) | 38 (93%) |
|  | Total | 82 (100%) | 79 (96%) |
| 6-month | CBT | 36 (88%) | 37 (90%)^a^ |
|  | ADepT | 35 (85%) | 34 (83%) |
|  | Total | 71 (87%) | 71 (87%) |
| 12-month | CBT | 31 (76%) | 28 (68%) |
|  | ADepT | 32 (78%) | 29 (71%) |
|  | Total | 63 (77%) | 57 (70%) |
| 18-month | CBT | 31 (76%) | 29 (70%) |
|  | ADepT | 29 (70%) | 25 (61%) |
|  | Total | 60 (73%) | 54 (66%) |

*Note –* Data are count (% of sample) values. ^a^ One participant did not attend assessment but did post back outcome measures when requested.

Table S6

Outcome data completeness rates on all clinical and health economic measures at each assessment.

|  | CBT |  |  |  |  | ADepT |  |  |  |  |
| --- | --- | --- | --- | --- | --- | --- | --- | --- | --- | --- |
|  | I | 6m | 12m | 18m | C | I | 6m | 12m | 18m | C |
| *PHQ-9 depression* | 41 | 37 | 28 | 30 | 33 | 41 | 35 | 29 | 25 | 32 |
| *WEMWBS wellbeing* | 41 | 37 | 28 | 29 | 33 | 38 | 34 | 29 | 25 | 30 |
| GAD-7 anxiety | 41 | 37 | 29 | 30 | 33 | 39 | 35 | 29 | 25 | 31 |
| SHAPS anhedonia | 40 | 37 | 29 | 30 | 32 | 38 | 34 | 29 | 25 | 30 |
| PANAS positive affect | 41 | 37 | 28 | 23 | 32 | 38 | 34 | 26 | 24 | 29 |
| PANAS negative affect | 41 | 37 | 28 | 23 | 32 | 38 | 34 | 26 | 24 | 29 |
| MASQ anhedonia | 40 | 37 | 29 | 30 | 32 | 37 | 34 | 29 | 25 | 29 |
| MASQ general distress | 40 | 37 | 29 | 30 | 32 | 37 | 34 | 29 | 25 | 29 |
| MASQ anxious arousal | 40 | 37 | 29 | 30 | 32 | 37 | 34 | 29 | 25 | 29 |
| WSAS functional impairment | 41 | 36 | 29 | 30 | 33 | 39 | 35 | 29 | 26 | 31 |
| IAPT phobia scale | 41 | 37 | 29 | 30 | 33 | 39 | 35 | 29 | 25 | 30 |
| HDRS depression interview | 41 | 36 | 31 | 30 | 33 | 40 | 35 | 32 | 29 | 32 |
| HARS anxiety interview | 41 | 36 | 31 | 30 | 33 | 41 | 35 | 32 | 29 | 33 |
| SCID depression interview | 41 | 36 | 31 | 31 | 29 | 41 | 35 | 32 | 29 | 27 |
| LIFE depression interview | - | 27 | 28 | - | - | - | - | 30 | 29 | - |
| EQ-5D-5L quality of life | 41 | 37 | 33 | 33 | 32 | 38 | 36 | 32 | 33 | 30 |
| ICECAP quality of life | 40 | 37 | 33 | 32 | 32 | 38 | 36 | 32 | 33 | 30 |
| AD-SUS care utilization | 41 | 35 | 31 | 31 | 32 | 41 | 36 | 32 | 28 | 31 |
| CBT/ADepT useage | 41 | 41 | 41 | 41 | 41 | 41 | 41 | 41 | 41 | 41 |

*Note:-* Data are count values (out of a sample of 41 in each arm); italics = co-primary outcome measures; I = intake; C=cumulative (area under curve for nearly all variables, except sum for AD-SUS and sustained remission across six-month, twelve-month, and eighteen-month assessments for SCID); LIFE interview only administered at twelve-month and eighteen-month assessment.

Continuation Rule Three: Engagement with Therapy

*Criterion: > 60% of sample recruited complete a minimum adequate dose of treatment*

To evaluate intervention engagement, the following metrics were reported: the number (%) of clients receiving a minimum adequate dose of acute therapy (attending at least 50% of the acute treatment maximum dose); and the mode and mean (SD) number of acute sessions attended. For the ADepT arm only, the number of clients engaging with booster sessions and mode and mean (SD) of booster sessions attendance were additionally reported. Further, the number (%) of participants who withdrew from the trial prior to randomisation and indicated that this was because they could not receive their preferred treatment and the number (%) of participants who withdrew from the trial during active treatment and stated this was due to dissatisfaction with the treatment were reported.

A minimum adequate dose of treatment (>50% of acute treatment sessions) was completed by 36/41(88%) of CBT and 35/41(85%) of ADepT participants (>60% target). A majority of individuals attended close to the full acute treatment dose (CBT mode=20, mean=16·66, SD=5·58; ADepT mode=15, mean=12·95, SD=4·33). Twenty-nine (81%) of 35 ADepT participants completing acute treatment engaged with at least one booster session (in this subgroup, mode=5, mean=4·34, SD=1·11; in the overall ADepT sample mode = 5, mean=3·07, SD=2·21). In the CBT arm, one individual withdrew after randomisation but before commencing treatment and four dropped out after treatment started but before completing a minimum adequate dose. No reasons for drop-out were captured by therapists or the research team. In the ADepT arm, one client dropped out after randomisation but before being allocated to a therapist, two clients dropped out due to external reasons (unable to afford travel due to benefits being withdrawn; unable to arrange childcare), and two clients gave reasons indicating they were dissatisfied with treatment (feeling too depressed to benefit, reporting therapy was not helping them). One further client had a planned discharge after seven sessions having met their treatment goals, so was not classified as a drop-out.

Continuation Rule Four: Intervention and Trial Safety

*Criterion: There are no unexpected and clearly trial- or treatment- related serious adverse events*

A combined Trial Steering Committee and Data Monitoring and Ethics Committee (TSC/DMEC) was convened to provide independent oversight of the trial, including monitoring trial progress and assessing any participant safety concerns. An adverse event reporting protocol was developed and followed. Adverse events were defined as any untoward medical occurrence or response, including a sustained and marked increase in depression severity (> six-point increase on PHQ-9 lasting two weeks), attempted suicide, and marked self-harm that put the individual at physical risk, or a significant flare up in pain or fatigue symptoms. Adverse events were further classified as serious adverse events if they were fatal, life threatening, required hospitalisation, resulted in significant disability or incapacity, or lead to any other serious condition judged significant by a clinician. All potential adverse events were reviewed by the trial principal investigator (BD). If events were judged to be serious and potentially trial or treatment related, they were then referred to the TSC/DMEC to review on ad hoc basis. To assess possible intervention harms further, the number of clients showing reliable deterioration from intake to six-month assessment on the PHQ-9 (> six-point increase), GAD-7 (> four-point increase), and WEMWBS (> seven-point decrease) was reported. Adverse events and serious adverse events in each arm (and decisions reached about whether each of these were trial- or treatment- related) was documented and reviewed by the TSC/DMEC at each meeting. No formal interim analyses were conducted by the TSC/DMEC and so no specific adjustments were made to minimise operational bias. Had any concerns come to light, the TSC/DMEC (in collaboration with sponsor and funder) would have recommended pausing the trial until a detailed review had been completed (at which point they would have recommended stopping, modifying or continuing the trial). Table S7 summaries the adverse event data. Reflecting the severity of the sample, there were two serious adverse events in each arm (two non-fatal overdoses in the CBT arm; one non-fatal overdose and one planned minor surgical procedure in the ADepT arm; each event in different participants). All of these events were referred to the TSC/DMEC to evaluate and were judged not to be trial or treatment related. There were also five adverse events in the CBT arm and seven adverse events in the ADepT arm. These were nearly all the risk protocol being enacted due to clients reporting suicidal ideation (none of which were judged to be trial- or treatment- related). One client (in the CBT arm) became upset answering questions during a research assessment, which was judged to be trial-related but to have been satisfactorily managed by the research team. A majority of adverse events occurred in different participants, although there were a number of participants in each arm who had multiple instances of the suicide risk protocol being enacted. No participants showed reliable deterioration on the PHQ-9, GAD-7, or WEMWBS. from intake to six-month assessment.

Table S7

Adverse event data.

| Arm/ID | Description | Classification |
| --- | --- | --- |
| *CBT* | (5 AEs, 2 SAEs) |  |
| AD15 | Client became upset during research assessment (AE, trial related) | AE, trial related |
| AD43 | Suicidal ideation where risk protocol enacted during treatment (3 times) | AE, not treatment related |
| AD52 | Suicide attempt (overdose), ongoing suicidal ideation where risk protocol was enacted (3 times) | SAE* and AE, not treatment related |
| AD58 | Suicide attempt (morphine overdose following relationship difficulties) | SAE*, not treatment related |
| AD67 | Suicidal ideation where risk protocol was enacted during treatment (2 times) | AE, not treatment related |
| AD95 | Suicidal ideation where risk protocol was enacted during treatment | AE, not treatment related |
| *ADepT* | (7 AEs, 2 SAEs) |  |
| AD16 | Suicidal ideation where risk protocol enacted (3 times during treatment) | AE, not treatment related |
| AD18 | Suicidal ideation where risk protocol was enacted (2 times during treatment) | AE, not treatment related |
| AD19 | Suicidal ideation where risk protocol was enacted during treatment | AE, not treatment related |
| AD29 | Planned gall bladder surgery | SAE*, not treatment related |
| AD30 | Suicide attempt (overdose) | SAE*, not treatment related |
| AD42 | Suicidal ideation where risk protocol was enacted during treatment | AE, not treatment related |
| AD49 | Suicidal ideation where risk protocol was enacted during treatment | AE, not treatment related |
| AD51 | Suicidal ideation where risk protocol was enacted during treatment | AE, not treatment related |
| AD60 | Suicidal ideation where risk protocol was enacted during treatment | AE, not treatment related |
| AD65 | Suicidal ideation where risk protocol was enacted during treatment | AE, not treatment related |

*Note:-* Arm/ID = treatment group/client id; AE = adverse event, SAE = serious adverse event; DMEC = Data Monitoring and Ethics Committee.

Continuation Rule Five: Intervention Acceptability and Feasibility

*Criterion: Any remaining concerns about the intervention or trial design can be rectified.*

Client Views on Intervention

Participants completed the Credibility/Expectancy Questionnaire (CEQ^31^) to gain their views on treatment after completing their first treatment session. Credibility is rated on a 9-point Likert scale range from 1 low credibility to 9 high credibility. Expectancy is rated in terms of mean anticipated percentage improvement in symptoms. CEQ data were available for 39/41 individuals in each arm. After acute treatment was completed, participants were asked to rate the acceptability of treatment and how satisfied they were with treatment (both on scales where 1 = not at all to 5 = extremely) and how likely they would be to recommend that treatment to friends or family (from 1 = extremely unlikely to 5 = extremely unlikely). Post-treatment rating data were returned by 34/41 (83%) of individuals in the CBT arm and 30/41 (73%) of individuals in the ADepT arm.

To assess intervention acceptability, the mean (SD) of credibility and expectancy ratings after session one, mean (SD) of post treatment ratings, and the number (%) of clients reporting that the intervention they received was acceptable, satisfactory, and that they would recommend it to others was reported (Table S8). Both treatments were rated as credible and participants reported some expectancy of improvement in symptoms (>50%) after session one. Post-treatment satisfaction and acceptability ratings were high (means > 4), with all participants at least moderately satisfied and finding treatment at least moderately acceptable. Recommendation ratings were also high (means>4), with >90% of participants being likely or very likely to recommend treatment to others (with remaining ratings being neither likely nor unlikely).

Table S8

Participant intervention ratings

|  | CBT | ADepT |
| --- | --- | --- |
| Session One Credibility Rating | 6·83 (1·23) | 7·06 (1·41) |
| Session One Expectancy Rating | 53% (24%) | 59% (21%) |
| End of Treatment Satisfaction Rating | 4·15 (0·74) | 4·23 (0·77) |
| Rating at least moderately satisfied | 34/34 (100%) | 31/31 (100%) |
| End of Treatment Acceptability Rating | 4·15 (0·66) | 4·33 (0·66) |
| Rating at least moderately acceptable | 34/34 (100%) | 31/31 (100%) |
| End of Treatment Recommendation Rating | 4·50 (0·66) | 4·53 (0·57) |
| Rating at least likely to recommend | 31/34 (91%) | 29/31 (94%) |

*Note*: data are complete case mean (SD) or count (%).

Competence, Fidelity and Differentiation of Intervention Delivery by Therapists

Therapists in both arms were experienced in high intensity CBT and had extensive prior mental health expertise. In both arm there was a mixture of therapists primarily employed in the AccEPT clinic (with prior experience of therapy delivery in a research context) and therapists hired in on a locum basis from local IAPT services to deliver therapy in the trial (with no prior experience of therapy delivery in a research context). All but one therapist had at least five years of experience of delivering high intensity CBT interventions. The remaining therapist (in the CBT arm) had extensive experience as a low-intensity therapist, supervisor and trainer and had recently qualified as a high intensity therapist. There was no clear difference in overall expertise or experience between arms. In the ADepT arm, two therapists were new to the ADepT model and two had previous experience of delivering in ADepT (as part of a previously published case series^33^). In the CBT arm, five out of six therapists had no training in ADepT. One therapist had been an ADepT therapist in the previously published ADepT case series^33^, but there was no evidence of contamination of his CBT practice in the current trial (adequate differentiation scores on all tapes; see Table S7). The choice of which therapists were allocated to each arm was pragmatic, based on which days they were available to attend supervision and if they had prior experience of delivering ADepT.

All clients gave consent for their sessions to be recorded. A vast majority of sessions (>95%) were successfully recorded. Where recordings were not successfully captured, this was due to therapists forgetting to record, recording equipment failing, data corruption when uploading the recording to the university server, too much background noise meaning the recording was poor quality, and on a handful of occasions sessions taking place in a setting where recording was not feasible (for example, walking outside). Twenty-four session recordings in each arm (6 from each of four ADepT therapists; four from each of six CBT therapists) were rated for competence, using the Revised Cognitive Therapy Scale (CTS-R^32^) in the CBT arm and via a bespoke ADepT Therapy Rating scale (Dunn et al., in preparation) in the ADepT arm.

The CTS-R consists of five general items (agenda setting and adherence; feedback; collaboration; pacing and efficient use of time; and interpersonal effectiveness) and eight cognitive therapy specific items (agenda setting and adherence; eliciting appropriate emotional expression; eliciting key cognitions; eliciting behaviours; guided discovery; conceptual integration; application of change methods; and homework setting). Therapists are rated on each item.

The ADepT therapy rating scale consists of three meta-domains (style, structure, and technique), each consisting of a number of behavioural specific competencies (see summary of competencies in Table S2; full copy of rating scale available on request from corresponding author Barney Dunn, [b.d.dunn@exeter.ac.uk](mailto:b.d.dunn@exeter.ac.uk)). Style competencies are therapist positive interpersonal style; thickening the positive narrative; moving from the informal to the formal; breaking tasks down into steps; working with the therapeutic alliance; changing client relationship to depression; and helping clients take ownership of change. Structural competences are therapist preparation; agenda setting; feedback; homework; consolidating learning; and pacing. Technical competences are values identification; goal setting and monitoring; case conceptualisation; use of cognitive change techniques; use of behavioural change techniques; use of interpersonal change techniques; use of emotional/bodily change techniques; and wellbeing planning. Therapists are rated on each meta-domain not each individual competence. This is because there is not an expectation that a competent session has to exhibit all of the competencies in each meta-domain, but instead that the competencies appropriate to the stage of therapy and session content will be effectively utilised.

On both the ADepT rating scale and the CTS-R, ratings are on a six-point scale that aligns to the Dreyfus competence framework. Scores range from 0 (absence of feature or highly inappropriate performance) to 6 (excellent performance), with the cut-off for competence being a score of at least 3 (competent, but some problems and/or inconsistencies). On the CTS-R, every item is individually assessed, with a total score of 36 as the cut-off for a tape being rated as competent. On the ADepT rating scale, each meta-domain is rated and the score on each domain has to be at least three for a tape to be rated as competent.

Two separate ratings for each tape also assessed fidelity to the protocol (“Was session delivered in a way that was consistent with the therapy manual?”) and differentiation between treatments (“Was session delivered in a way that distinguished it from the comparator arm?”). Fidelity was rated on a scale from 1 (not at all consistent with protocol) to 10 (very much consistent with the protocol), with scores > 5 indicating adequate fidelity. Differentiation was rated on a bipolar score ranging from 1 (very like CBT) to 9 (very like ADepT), with a score of 5 indicating the tape was equally like CBT or ADepT. Scores < 5 indicated adequate differentiation in the CBT arm, whereas scores > 5 indicated adequate differentiation in the ADepT arm. Raters were all experienced in the therapy they were rating and had read the relevant trial treatment protocol. Raters were given a series of behavioural descriptors of features that differentiate ADepT from CBT to help inform the differentiation rating.

Session recordings for rating were randomly selected in terms of client and session number, apart from stratification by therapy stage (half of tapes in first half of acute sessions; half of tapes in second half of acute sessions). CBT ratings were completed by two of the CBT therapists in the trial and ADepT ratings were done by the two more experienced of the ADepT therapists in the trial. Therapists did not rate their own tapes. Raters underwent training and standardization prior to reviewing the tapes. If in doubt about how to rate a particular session, a second opinion was sought from another member of the trial team.

To assess inter-rater reliability of the novel ADepT rating scale, a subset of randomly selected tapes (two for each therapist) was additionally coded by a second rater (an additional therapist trained in ADepT). Satisfactory inter-rater reliability was defined as the same overall binary judgement of whether a tape was competent or not, met fidelity criteria or not, and met differentiation criteria or not, and there not being more than a single category difference in continuous competence classification (e.g., if a tape was rated as proficient by one assessor, there was satisfactory reliability if the tape was rated as competent, proficient, or expert by the second assessor). To assess the quality of the therapy that was delivered in each arm using these rating scales, the number of therapists and therapy tapes that were coded that met competence, fidelity and differentiation criteria was reported. Further, it was examined whether there was any clustering by therapist of client drop-out before completing a minimum adequate therapy dose. Inter-rater reliability of ADepT tapes was satisfactory, with the eight tapes that were dual rated all receiving the same overall binary competence, differentiation and fidelity classifications (meeting or not meeting threshold). With regards to continuous competence ratings, five tapes received exactly the same rating and three tapes differed in their rating (one assessor judging them to be proficient and another assessor judging them to be expert, with no consistent bias as to which rater gave the higher judgement).

Table S9 summarises the number of clients treated by each therapist (and the number who dropped out of treatment with each therapist). It also summarises competency, fidelity and differentiation tape rating data for each therapist. All six CBT therapists met overall competency, fidelity and differentiation cut offs when averaging across tapes (mean competence > 3, mean fidelity > 5, mean differentiation > 5 [more like CBT than ADepT]) and when looking at each tape individually. Four therapists were on average rated as competent to good (mean competency score of between 3 and 4) and two therapists were on average rated as good to very good (mean competency score of between 4 and 5). There was no obvious clustering of therapy drop-outs in the CBT arm.

All four ADepT therapists also met overall overall competence, fidelity and differentiation cut-offs when averaging across tapes (mean competence > 3, mean fidelity > 5, mean differentiation < 5 [more like ADepT than CBT]). When looking at individual tapes, two therapists (both with prior experience of ADepT) met all criteria for all tapes. One of the two therapists new to ADepT met competence and differentiation criteria for all tapes, but one tape was scored as having borderline fidelity (rated as 5). The other therapist new to ADepT (therapist three) met all criteria for four tapes but showed borderline competence (ratings of 2.33 and 2.55), fidelity (ratings of 5 and 5), and differentiation (ratings of 5 and 5) criteria for two tapes. Three out of four ADepT therapists were rated as good to very good (mean competence rating score between 4 and 5) and one (therapist three) was rated as competent to good (mean competence ratings score between 3 and 4). There was some evidence of clustering of treatment drop-outs, with all four clients dropping out in the ADepT arm being treated by therapist three. This therapist treated 12 clients overall, meaning they had a 33% drop-out rate.

Table S9

Therapist demographics, experience and competence, fidelity and differentiation ratings.

| Therapist | Treated  (dropped out) | Competence  mean (SD),  classification | Fidelity  mean (SD), classification | Differentiation mean (SD), classification |
| --- | --- | --- | --- | --- |
| ADepT 1 | 6 (0) | 4.69 (0.52), 6/6 | 7.50 (0.55), 6/6 | 7.67 (0.82), 6/6 |
| ADepT 2 * | 10(0) | 4.19 (0.76), 6/6 | 7.17 (1.33), 5/6 | 7.33 (0.88), 6/6 |
| ADepT 3 | 12(4) | 3.75 (1.12), 4/6 | 6.50 (1.38), 4/6 | 6.50 (1.38), 4/6 |
| ADepT 4 * | 12(0) | 4.44 (0.66), 6/6 | 8.33 (1.51), 6/6 | 7.81 (1.17), 6/6 |
| *ADepT Overall* |  | *4.27 (0.83)* | *7.33 (1.66)* | *7.33 (1.14)* |
| CBT 1 | 4(0) | 3.50 (0.50), 4/4 | 7.00 (1.83), 4/4 | 2.00 (0.00), 4/4 |
| CBT 2 | 12(0) | 3.80 (0.28), 4/4 | 6.00 (0.00), 4/4 | 3.50 (0.58), 4/4 |
| CBT 3 | 4(1) | 3.92 (0.50), 4/4 | 8.50 (1.73), 4/4 | 1.25 (0.50), 4/4 |
| CBT 4 | 4(0) | 4.14 (0.44), 4/4 | 7.00 (0.82), 4/4 | 3.00 (0.00), 4/4 |
| CBT 5 + | 12(2) | 4.06 (0.18), 4/4 | 7.75 (1.50), 4/4 | 1.00 (0.00), 4/4 |
| CBT 6 | 4(1) | 3.52 (0.39), 4/4 | 7.75 (2.63), 3/4 | 1.25 (0.50), 4/4 |
| *CBT Overall* |  | *3.77 (0.45)* | *7.38 (1.34)* | *2.00 (1.02)* |

*Note:-* Table reports complete case data; classification data = number of tapes meeting criteria out of tapes assessed; competence cut-off >3; fidelity cut off >5; differentiation cut-off >5 for ADepT and <5 for CBT. *=therapist new to ADepT in trial; +=CBT therapist with prior experience of ADepT.

Evaluation of Broader Trial Procedures

The following data were reported regarding broader trial procedures: number of failures in the external web-based system conducting randomisation; number of times there were problems with allocation after randomisation; balance between arms at intake assessment in the trial stratification variables; and the number of data protection breaches or other violations of confidentiality that occurred. The functioning of the trial procedures to assess adverse events and manage client risk was narratively summarised. To examine feasibility of the health economic methodology, it was investigated whether particular resource use categories were missing or were misunderstood on the AD-SUS (and any difficulties researchers experienced when completing AD-SUS assessments with participants were logged). To evaluate if the assessor blinding was successful, assessors reported at the primary six-month assessment whether the blind had been broken and if not to guess which arm clients were allocated to. To assess efficiency of allocation process to treatment after randomisation, the mean (SD) delay between randomisation and first treatment session was reported. To capture participants’ views on the key outcome measures (PHQ-9, WEMWBS, GAD-7, PANAS and SHAPS), at eighteen-month assessment participants were given a copy of the measures to look at and were then asked to rate to what extent they agreed with the statement that the measure captured what it is important to improve on during treatment for depression (on a scale 1 strongly agree to 5 strongly disagree). Participants also ranked the measures in terms of how useful an outcome they would be in a subsequent trial (from 1 best to 5 worst).

Trial randomisation, allocation and concealment processes worked as intended, with the external web-based system resulting in an acceptable balance between arms on the trial stratification variables (66% of participants in both arms currently taking anti-depressants; 51% severely depressed in CBT versus 46% severely depressed in ADepT). There were no failures of randomisation or problems encountered during allocation. Data protection procedures worked as intended and there were no confidentiality breaches. Adverse event assessment procedures worked effectively, with the DMEC/TSC providing rapid and thorough assessment of whether serious adverse events were potentially trial- or treatment- related. The trial risk protocol around suicidal intentions or acts also functioned adequately. Health economic procedures worked effectively. There was no evidence of increased rates of missingness in, or misunderstanding of, any resource use categories in the AD-SUS. Researchers reported the only difficulty they regularly encountered was gaining exact doses of medications participants had been using, particularly when changes to prescriptions had been made during the follow-up period.

Blind data at six-month assessment were available for 35/41 participants (85%) in each arm. The blind was broken in 6/35 assessments (17%) in the CBT arm and in 4/35 assessments (11%) in the ADepT arm. Reasons given for the blind being broken were the client mentioned the name of their therapist, the client told the assessor which arm they were in, or therapists had mentioned to the assessor that one of their clients was due to be seen by them. In sessions where the blind was not broken, assessors could not guess allocation at above chance levels (45% guess accuracy in CBT and 45% guess accuracy in ADepT).

The mean wait from randomisation to first treatment session was 17 days (SD=8.92; maximum=38 days) in the CBT arm and 21 days (SD=12.53; maximum= 62 days) in the ADepT arm. Where there were longer waits, this was due to client availability (extended annual leave or desire to start treatment at a particular time to align with other life circumstances) or therapist capacity (clients having limited availability as to times they could attend treatment and needing to wait for a therapist to have a treatment slot free at those times).

The questionnaire asking participants to evaluate some of the outcome measure ratings was filled in at the end of the study by 52/82(63%) participants (28/41[68%] in CBT, 24/41[58%] in ADepT), although not all participants completed all items. Table S10 summarises mean importance ratings, the proportion of clients agreeing or strongly agreeing that the measure captured what was important, mean relative ranking data, and the proportion of clients rating that scale as their preferred (or joint-preferred) outcome measure. We had no reason to expect group differences in these ratings, so collapsed across therapy arms. The two candidate primary outcome measures were ranked most highly and >80% of individuals judged them as capturing what was important. However, therapists and clients both narratively commented none of the measures adequately captured functional recovery. As only a subset of participants completed these ratings, they should be interpreted tentatively.

Table S10

Evaluation of candidate outcome measures.

|  | Importance ratings | Agree Important | Relative  ranking | Top Rank |
| --- | --- | --- | --- | --- |
| PHQ9 depression | 1·88 (1·03) | 42/51 (82%) | 2·45 (1·76) | 25/49 (51%) |
| WEMWBS wellbeing | 1·90 (0.96) | 37/46 (80%) | 2·56 (1·43) | 15/48 (31%) |
| GAD7 anxiety | 1·97 (1·12) | 38/50 (76%) | 3·14 (1·66) | 12/49 (25%) |
| SHAPS anhedonia | 1·87 (1·08) | 39/47 (83%) | 3·15 (1·73) | 10/48 (21%) |
| PANAS positive affect | 2·22 (0·95) | 34/50 (68%) | 3·77 (1·59) | 6/48 (13%) |
| PANAS negative affect | 2·12 (0·96) | 35/50 (70%) | 3·73 (1·66) | 6/48 (13%) |

*Note* – Table reports complete case data; importance on a scale from 1 very important to 5 not at all important; ranking on a scale from 1 highest importance to 5 lowest importance.

Suggested Refinements to Definitive Trial Design

All pre-specified feasibility continuation rules were met. However, some minor amendments are required to optimize subsequent definitive trial design. While data completeness at six months was satisfactory, there was a slight reduction in return rates at longer-term follow-up. To enhance rates of data completeness, the measurement burden should be reduced and there should be a move to electronic rather than pen-and-paper measurement. While ADepT was delivered competently, several therapists recommended an extended training phase prior to treating clients in a trial. An extended initial training and supervision (and competency assessment) of at least two cases is recommended for therapists before they enter a subsequent trial. While the primary outcome measures were rated positively, there is also a need to select a measure that captures functional recovery from depression.

**5: Additional Clinical Proof-of-Concept Analyses**

Within-Group Analyses

Table S11 reports the effect size (and 95% confidence interval) of paired sample t-tests examining change from intake to six months on each outcome variable in each group.

Table S11

Summary of within-group analyses comparing intake to six-month scores on each continuous outcome.

|  | CBT  *N* | Effect size | ADepT  *N* | Effect size |
| --- | --- | --- | --- | --- |
| *PHQ-9 depression* | 37 | 1·44 (1·14, 1·75) | 35 | 1·61 (1·31, 1·92) |
| *WEMWBS wellbeing* | 37 | 1·11 (0·80, 1·41) | 32 | 1·31 (1·01, 1·62) |
| GAD-7 anxiety | 37 | 0·99 (0·69, 1·30) | 34 | 1·64 (1·34, 1·95) |
| SHAPS anhedonia | 36 | 0·83 (0·52, 1·14) | 32 | 1·43 (1·13, 1·74) |
| PANAS positive affect | 37 | 1·01 (0·70, 1·31) | 32 | 1·48 (1·17, 1·78) |
| PANAS negative affect | 37 | 1·00 (0·69, 1·31) | 32 | 1·23 (0·92, 1·53) |
| MASQ anhedonia | 36 | 0·91 (0·60, 1·21) | 32 | 1·35 (1·05, 1·66) |
| MASQ general distress | 36 | 0·98 (0·67, 1·28) | 32 | 1·70 (1·39, 2·01) |
| MASQ anxious arousal | 36 | 0·68 (0·37, 0·99) | 32 | 0·93 (0·62, 1·23) |
| WSAS functional impairment | 36 | 0.75 (0.44, 1.05) | 34 | 0.95 (0.64, 1.26) |
| IAPT phobia scale | 37 | 0·61 (0·30, 0·92) | 34 | 0·77 (0·47, 1·08) |
| HDRS depression | 36 | 1·81 (1·50, 2·11) | 34 | 1·76 (1·45, 2·06) |
| HARS anxiety | 36 | 1·24 (0·93, 1·55) | 35 | 1·30 (1·00, 1·61) |

*Note:-* Primary outcomes italicized; N=number of participants with complete intake and six month data; t = t-value from intent-to-treat paired sample t-test (using multiple imputation to simulate missing values) at six months; effect sizes = Cohen’s d (95% confidence interval) from intent-to-treat paired sample t-tests (using multiple imputation to simulate missing values).

Twelve-Month and Eighteen-Month Outcomes

All continuous analyses were completed on twelve-month and eighteen-month follow-up data (Table S12). The vast majority of twelve-month and eighteen-month analyses continued to favour ADepT over CBT, although the confidence intervals were broader than in the primary six-month and secondary eighteen-month AUC analyses and non-inferiority (lower bound confidence interval ds>-0·24) were only established on a subset of secondary outcomes (twelve months: SHAPS anhedonia, MASQ general distress, WSAS functioning, and IAPT phobia; eighteen months: WEMWBS wellbeing, PANAS PA and NA, MASQ anhedonia, WSAS functioning and IAPT phobia). The mean effect size was greater for CBT than ADepT in three analyses: MASQ anxious arousal at twelve- and eighteen-month assessment and HARS anxiety at twelve-month assessment.

Table S12

Predicted raw and standardized score continuous differences between arms in twelve-month and eighteen-month analyses.

|  | *N* | Raw ∆ | Standardized d ∆ |
| --- | --- | --- | --- |
| **Twelve months** |  |  |  |
| *PHQ9 depression* | 28/29 | -1·00 (-3·76, 1·76) | 0·17 (-0·30, 0·64) |
| *WEMWBS wellbeing* | 28/27 | 1·52 (-3·40, 6·45) | 0·14 (-0·32, 0·61) |
| GAD7 anxiety | 29/28 | -0·66 (-3·20, 1·89) | 0·13 (-0·36, 0·61) |
| SHAPS anhedonia | 28/27 | -1·70 (-5·17, 1·76) | 0·22 (-0·23, 0·68)+ |
| PANAS positive affect | 28/25 | 1·65 (-2·76, 6·07) | 0·18 (-0·30, 0·66) |
| PANAS negative affect | 28/25 | -1·48 (-5·17, 2·21) | 0·19 (-0·28, 0·66) |
| MASQ anhedonia | 28/26 | -0·19 (-6·16, 2·39) | 0·02 (-0·27, 0·69) |
| MASQ general distress | 28/26 | -2·02 (-5·67, 1·63) | 0·24 (-0·19, 0·68)+ |
| MASQ anxious arousal | 28/26 | 1·56 (-1·25, 4·38) | -0·25 (-0·69, 0·20) ^a^ |
| WSAS functioning | 29/28 | -2·11 (-5·96, 1·75) | 0·23 (-0·19, 0·64)+ |
| IAPT phobia scale | 29/27 | -1·37 (-3·61, 0·87) | 0·25 (-0·16, 0·65)+ |
| HDRS depression | 31/31 | -0·97 (-4·02, 2·08) | 0·15 (-0·32, 0·61) |
| HARS anxiety | 31/32 | 0·54 (-2·16, 3·23) | -0·09 (-0·52, 0·35) ^a^ |
| **Eighteen months** |  |  |  |
| *PHQ9 depression* | 30/25 | -0·52 (-3·73, 2·68) | 0·08 (-0·39, 0·54) |
| *WEMWBS wellbeing* | 29/24 | 3·87 (-2·21, 9·95) | 0·32 (-0·18, 0·82)+ |
| GAD7 anxiety | 30/25 | -0·94 (-3·77, 1·9) | 0·17 (-0·34, 0·67) |
| SHAPS anhedonia | 30/24 | -1·24 (-5·66, 3·18) | 0·14 (-0·36, 0·64) |
| PANAS positive affect | 23/24 | 3·97 (-0·89, 8·84) | 0·43 (-0·10, 0·96)+ |
| PANAS negative affect | 23/24 | -4·54 (-9·66, 0·58) | 0·45 (-0·06, 0·96)+ |
| MASQ anhedonia | 30/23 | -2·86 (-7·36, 1·64) | 0·29 (-0·16, 0·73)+ |
| MASQ general distress | 30/23 | -0·74 (-5·77, 4·29) | 0·07 (-0·42, 0·56) |
| MASQ anxious arousal | 30/23 | 2·51 (-1·17, 6·19) | -0·34 (-0·83, 0·16) ^a^ |
| WSAS functioning | 30/24 | -4·24 (-9·98, 1·49) | 0·37 (-0·13, 0·87)+ |
| IAPT phobia scale | 29/24 | -1·63 (-4·69, 1·44) | 0·25 (-0·22, 0·72)+ |
| HDRS depression | 30/28 | -1·61 (-5·49, 2·38) | 0·2 (-0·29, 0·67) |
| HARS anxiety | 30/29 | -0·99 (-4·55, 2·5) | 0·13 (-0·34, 0·62) |

*Note:* Primary outcomes italicized; *N* = participants with complete data in CBT/ADepT arm; all other data are mean (95% CI) values; Raw∆ = estimated raw score difference between groups (ADepT – CBT) from intent-to-treat linear regression (using multiple imputation to simulate missing values); d∆ = estimated standardized (Cohen’s d) difference between groups (ADepT – CBT) from intent-to-treat linear regression (using multiple imputation to simulate missing values); + = lower bound CI > -0·24; ^a^ = effect size estimate favours CBT (in all other cases effect size estimates favour ADepT).

Reliable Change (RC) and Reliable and Clinically Significant Change (RSCS) Analyses

Table S13 reports the results of RC and RCSC analyses at each time point. Rates numerically favoured ADepT over CBT for all outcomes at each assessment, apart from: MASQ-S30 anxious arousal RC at twelve months and eighteen months, HDRS depression RC at six months and twelve months, and HARS anxiety at twelve months. Confidence intervals of the effect sizes were broad, although did not cross one in a subset of analyses (RC and RCSC at six months, HARS anxiety RC at six months, combined PHQ9/WEMWBS RCSC at six months, WEMWBS wellbeing RC and RCSC at eighteen months, PANAS PA RC at eighteen months). RC and RCSC were not calculated for IAPT phobia as there are no reliability estimates and no general population normative data available.

Table S13

Reliable change (RC) and reliable and clinically significant change (RCSC) analyses at six-month, twelve-month and eighteen-month follow-up.

|  | 6m |  |  | 12m |  |  | 18m |  |  |
| --- | --- | --- | --- | --- | --- | --- | --- | --- | --- |
|  | CBT | ADepT | Odds ratio  (95% CI) | CBT | ADepT | Odds ratio  (95% CI) | CBT | ADepT | Odds ratio  (95% CI) |
| **RC** |  |  |  |  |  |  |  |  |  |
| PHQ9 depression | 0·73 | 0·77 | 1·09 (0·38, 3·16) | 0·71 | 0·83 | 2·12 (0·58, 7·85) | 0·67 | 0·88 | 2·86 (0·64, 12·55) |
| WEMWBS wellbeing | 0·59 | 0·72 | 1·63 (0·60, 4·48) | 0·68 | 0·70 | 1·22 (0·39, 3·82) | 0·48 | 0·71 | 3·13 (1·03, 9·68) |
| PHQ9/WEMWBS | 0·51 | 0·69 | 1·84 (0·66, 5·10) | 0·61 | 0·70 | 1·82 (0·59, 5·64) | 0·45 | 0·67 | 2·83 (0·94, 8·50) |
| GAD7 anxiety | 0·33 | 0·53 | 2·41 (0·84, 6·96) | 0·64 | 0·70 | 1·34 (0·39, 4·53) | 0·57 | 0·67 | 1·55 (0·51, 4·71) |
| SHAPS anhedonia | 0·38 | 0·65 | 3·03 (1·11, 8·25) | 0·45 | 0·65 | 2·25 (0·71, 7·10) | 0·40 | 0·56 | 1·97 (0·64, 6·11) |
| PANAS positive affect | 0·65 | 0·75 | 1·75 (0·57, 5·37) | 0·61 | 0·68 | 1·62 (0·55, 4·76) | 0·65 | 0·91 | 5·05 (1·11, 22·87) |
| PANAS negative affect | 0·46 | 0·56 | 1·46 (0·55, 3·90) | 0·46 | 0·56 | 1·20 (0·39, 3·71) | 0·43 | 0·57 | 1·92 (0·58, 6·36) |
| MASQ-S30 anhedonia | 0·64 | 0·75 | 1·70 (0·57, 5·05) | 0·61 | 0·77 | 2·39 (0·58, 9·78) | 0·63 | 0·78 | 2·16 (0·67, 6·96) |
| MASQ-S30 general distress | 0·53 | 0·75 | 2·75 (0·92, 8·33) | 0·64 | 0·81 | 2·64 (0·69, 10·28) | 0·47 | 0·56 | 1·48 (0·52, 4·22) |
| MASQ-S30 anxious arousal | 0·14 | 0·19 | 1·58 (0·41, 6·17) | 0·18 | 0·11 | 0·59 (0·16, 2·18)^a^ | 0·17 | 0·08 | 0·59 (0·12, 2·8)^a^ |
| WSAS functioning | 0·33 | 0·44 | 1·70 (0·61, 4·81) | 0·38 | 0·46 | 1·60 (0·47, 5·37) | 0·30 | 0·46 | 2·20 (0·70, 7·10) |
| HDRS depression | 0·89 | 0·88 | 1·02 (0·30, 3·56) | 0·87 | 0·84 | 0·79 (0·19, 3·19)^a^ | 0·70 | 0·78 | 1·57 (0·53, 4·71) |
| HARS anxiety | 0·39 | 0·63 | 2·92 (1·05, 8·00) | 0·52 | 0·50 | 0·90 (0·33, 2·44)^a^ | 0·43 | 0·48 | 1·27 (0·41, 3·90) |
| **RCSC** |  |  |  |  |  |  |  |  |  |
| PHQ9 depression | 0·49 | 0·69 | 2·25 (0·81, 6·23) | 0·46 | 0·62 | 1·92 (0·61, 5·99) | 0·50 | 0·60 | 1·51 (0·48, 4·81) |
| WEMWBS wellbeing | 0·35 | 0·53 | 2·23 (0·83, 5·93) | 0·36 | 0·52 | 2·20 (0·73, 6·62) | 0·31 | 0·58 | 3·42 (1·26, 10·38) |
| PHQ9/WEMWBS | 0·32 | 0·59 | 2·83 (1·04, 7·54) | 0·46 | 0·59 | 1·92 (0·63, 5·87) | 0·38 | 0·54 | 1·79 (0·59, 5·47) |
| GAD7 anxiety | 0·17 | 0·38 | 2·83 (0·90, 8·85) | 0·32 | 0·48 | 1·99 (0·67, 5·93) | 0·33 | 0·58 | 2·86 (0·98, 8·33) |
| SHAPS anhedonia | 0·35 | 0·65 | 3·82 (1·36, 10·80) | 0·38 | 0·54 | 1·88 (0·66, 5·42) | 0·33 | 0·52 | 2·92 (0·92, 9·30) |
| PANAS positive affect | 0·47 | 0·65 | 1·97 (0·73, 5·37) | 0·46 | 0·56 | 1·70 (0·53, 5·37) | 0·52 | 0·70 | 2·20 (0·58, 8·41) |
| PANAS negative affect | 0·35 | 0·47 | 1·88 (0·69, 5·10) | 0·39 | 0·40 | 0·95 (0·34, 2·64)^a^ | 0·39 | 0·56 | 1·93 (0·63, 5·93) |
| MASQ-S30anhedonia | 0·33 | 0·59 | 2·92 (0·99, 8·50) | 0·50 | 0·54 | 1·38 (0·44, 4·31) | 0·43 | 0·56 | 1·68 (0·63, 4·53) |
| MASQ-S30 general distress | 0·36 | 0·56 | 2·29 (0·84, 6·36) | 0·25 | 0·50 | 3·25 (0·91, 11·59) | 0·37 | 0·44 | 1·51 (0·53, 4·26) |
| MASQ-S30 anxious arousal | 0·05 | 0·16 | 2·01 (0·54, 7·61) | 0·14 | 0·11 | 0·84 (0·21, 3·35)^a^ | 0·07 | 0·08 | 1·75 (0·25, 12·06) |
| WSAS functioning | 0·19 | 0·24 | 1·54 (0·48, 4·90) | 0·21 | 0·32 | 1·75 (0·54, 5·70) | 0·17 | 0·33 | 2·64 (0·75, 9·30) |
| HDRS depression | 0·36 | 0·55 | 2·16 (0·80, 5·81) | 0·48 | 0·61 | 1·62 (0·57, 4·53) | 0·53 | 0·54 | 1·07 (0·38, 3·03) |
| HARS anxiety | 0·28 | 0·48 | 2·29 (0·84, 6·30) | 0·45 | 0·40 | 0·84 (0·30, 2.34)^a^ | 0.30 | 0·45 | 1·95 (0·67, 5·70) |

*Note:* Table reports (complete case) proportion of clients in each arm meeting criteria; effect sizes are odds ratios (95% CI) estimates from ITT logistic regression analyses (using multiple imputation to simulate missing values), with scores > 1 showing greater rates of meeting criteria in ADepT over CBT; ^a^ = effect size estimate favours CBT (in all other cases effect size estimates favour ADepT)

Survival Analysis and Sustained Remission at Twelve Months

Of those who had remitted at six months, twelve-month LIFE interview follow-up data were available for 18/20 individuals receiving CBT (6[33%] of whom relapsed) and 24/28 individuals receiving ADepT (4[17%] of whom relapsed) (Figure S3). Cox’s regression estimated the odds of relapsing were approximately half as likely following ADepT than CBT, odds ratio = ·49 (95% CI = 0·14, 1.73).

SCID data at both the six- and twelve-month assessments were available for 31/41(76%) individuals in the CBT arm and 32/41(78%) individuals in the ADepT arm. Rates of sustained remission (meeting remission criteria at both the six-month and twelve-month follow-up assessment) were 15/31(48%) in CBT and 23/32(72%) in ADepT, with logistic regression suggesting the odds of meeting this criterion were approximately doubled in ADepT relative to CBT, odds ratio = 2.71 (95% CI = 0·92, 8.02).

Figure S3

Survival plot examining depressive relapse at twelve-month follow-up in the CBT and ADepT arms for the subset of individuals who had recovered at six months.


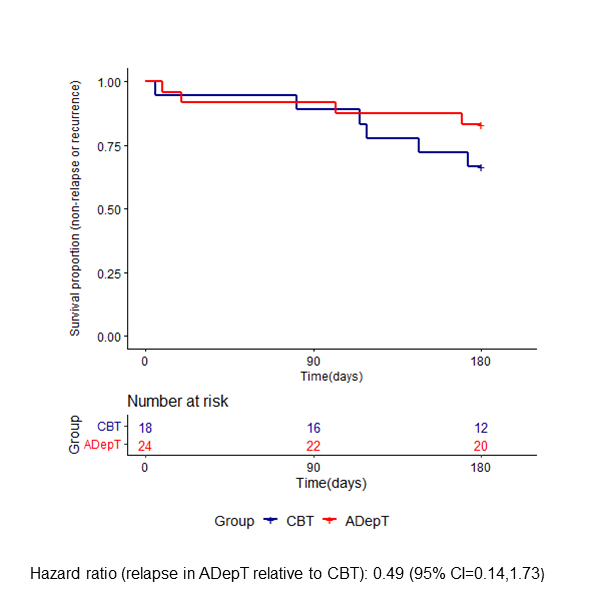


Benchmarking Analysis

Table S14 reports within-arm effect sizes estimates from intake to six months in each arm of the current trial and the comparator COBRA and COBALT trials (all estimated using complete case data). On all outcomes, within-arm effect sizes in the ADepT arm of the current trial were numerically larger than, the effect sizes observed in all active conditions in other trials. Within-effect size in the CBT arm of the current trial were numerically broadly equivalent to or slightly larger than those seen in active conditions of other trial. Table S15 reports estimates of raw score differences between each arm in the current trial to each arm of the comparator COBRA and COBALT trials (with associated 95% confidence intervals) emerging from linear regression analyses. Both arms in the current trial were superior to the COBALT TAU arm at repairing PHQ9 and GAD7 (95% confidence intervals did not cross zero). The current trial CBT arm performed broadly similarly or led to greater gains on all measures relative to the other active arms in COBALT and COBRA (confidence intervals crossing zero in all cases) and led to greater gains on all measure relative to the TAU COBALT arm (confidence intervals not crossing zero). The current trial ADepT arm led to greater gains on all measures relative to the other active therapies in COBALT and COBRA (confidence intervals not crossing zero for GAD-7 anxiety and SHAPS anhedonia relative to COBRA CBT and COBRA BA; confidence intervals crossing zero for other measures) and led to greater gains on all measures relative to COBALT TAU (confidence intervals not crossing zero).

Table S14

Benchmarking intake to six-month PHQ-9 depression, GAD-7 anxiety and SHAPS anhedonia change within each arm in the current trial against COBRA and COBALT trial arms.

|  | Within arm effect size  (intake to 6m change)  Cohen’s d(95% CI) |
| --- | --- |
| **PHQ-9** |  |
| ADepT ADepT | 1·61 (1·31, 1·92) |
| ADepT CBT | 1·44 (1·14, 1·75) |
| COBRA CBT | 1·18 (0·99, 1·34) |
| COBRA BA | 1·13 (0·96, 1·29) |
| COBALT CBT | 1·07 (0·93, 1·21) |
| COBALT TAU | 0·56 (0·43, 0·70) |
| **GAD-7 anxiety** |  |
| ADepT ADepT | 1·64 (1·34, 1·95) |
| ADepT CBT | 0·99 (0·69, 1·30) |
| COBRA CBT | 0·87 (0·72, 1·01) |
| COBRA BA | 0·82 (0·68, 0·97) |
| COBALT CBT | 0·83 (0·69, 0·96) |
| COBALT TAU | 0·40 (0·27, 0·54) |
| **SHAPS anhedonia** |  |
| ADepT ADepT | 1·43 (1·13, 1·74) |
| ADepT CBT | 0·83 (0·52, 1·14) |
| COBRA CBT | 0·81 (0·66, 0·97) |
| COBRA BA | 0·82 (0·67, 0·98) |

*Note:* Effect size estimates from complete case paired sample t-tests

Table S15

Benchmarking six-month PHQ-9 depression, GAD-7 anxiety and SHAPS anhedonia change between arms of the current trial and COBRA and COBALT trial arms.

|  | Current trial CBT | Current trial ADepT |
| --- | --- | --- |
| **PHQ-9** |  |  |
| COBRA CBT | 0·23 (-2·13, 2·60) | -1·45 (-3·86, 0·97) |
| COBRA BA | 0·11 (-2·26, 2·47) | -1·41 (-3·81, 0·99) |
| COBALT CBT | -0·02 (-2·20, 2·16) | -1·35 (-3·59, 0·89) |
| COBALT TAU | -2·92 (-5·10, -0·75) | -3·88 (-6·63, -2·17) |
| **GAD-7** |  |  |
| COBRA CBT | -0·16 (-2·08, 1·76) | -2·08 (-4·06, -0·11) |
| COBRA BA | -0·16 (-2·07, 1·75) | -2·01 (-3·97, -0·06) |
| COBALT CBT | 0·02 (-1·80, 1·85) | -1·78 (-3·66, 0·10) |
| COBALT TAU | -2·71 (-4·46, -·95) | -4·67 (-6·48, -2·87) |
| **SHAPS** |  |  |
| COBRA CBT | -0·16 (-1·74, 2·91) | -2·83 (-5·23, -0·43) |
| COBRA BA | 0·54 (-1·87, 2·96) | -2·90 (-5·40, -0·40) |

*Note:-* MCID estimates for PHQ-9 = 1·70 points and for GAD-7 = 1·15 points; there are no established estimates for the SHAPS. Data are estimated difference between groups (and associated 95% confidence intervals) in raw score units from linear regression (using complete case data).

Finally, we also benchmarked remission and response outcomes to meta-analytic findings of depression psychotherapies. Cuijpers and colleagues^34^ report rates of clients no longer meeting diagnostic criteria and meeting remission and recovery criteria from 92 studies with 181 conditions (134 psychotherapy, 47 control conditions; 6937 clients meeting criteria for major depressive disorder). In this meta-analysis, 62% of clients across all therapies (66% CBT) no longer met diagnostic criteria for depression after treatment on structured clinical interviews, compared to 56% remission in the CBT arm and 80% remission in the ADepT arm in the current trial. Similarly, the meta-analyses reported remission criteria on the Hamilton Depression Rating Scale (HDRS) 17-item version were met by 43% of clients across all therapies (49% CBT), compared to 39% remission in the CBT arm and 60% remission in the ADepT arm in the current trial. The meta-analyses reported response criteria (defined as 50% reduction on symptoms across all measures) were met by 48% of clients across all therapies (53% CBT), compared to PHQ-9 response rates of 46% in CBT and 66% in ADepT in the current trial. Mirroring the COBRA and COBALT benchmarking analyses, the CBT arm performed broadly equivalently to previous CBT trials, whereas ADepT tended to show greater levels of remission and response relative to previous trials. All benchmarking analyses should be viewed as preliminary given they are not comparisons of randomized groups.

Bayesian Analyses

Figure S4 summarises the results of twelve-month and eighteen-month Bayesian analyses on the PHQ-9 and WEMWBS, plotting the probability of difference in raw score outcomes if new clients were treated with ADepT rather than CBT at each time point. For the PHQ-9 at twelve months, 76% of participants would show numerically greater improvement (and 56% would show at least a MCID advantage) in ADepT relative to CBT. Less than 10% would show at least MCID disadvantage on the PHQ-9 in ADepT relative to CBT at twelve months. For the PHQ-9 at eighteen months, 87% of participants would show numerically greater improvement (and 71% would show at least a MCID advantage) in ADepT relative to CBT. Less than 6% would show at least MCID disadvantage on the PHQ-9 in ADepT relative to CBT at eighteen months. For the WEMWBS at twelve months, 76% of participants would show numerically greater improvement (and 40% would show at least a MCID advantage) in ADepT relative to CBT. Less than 4% would show at least MCID disadvantage on the WEMWBS in ADepT relative to CBT at twelve months. For the WEMWBS at eighteen months, 86% of participants would show numerically greater improvement (and 61% would show at least a MCID advantage) in ADepT relative to CBT. Less than 2·5% would show at least MCID deterioration on the WEMWBS in ADepT relative to CBT at eighteen months.

Figure S4

Probability of difference in PHQ-9 (a) and WEMWBS (b) outcomes between treatment arms in twelve-month and eighteen-month Bayesian analyses


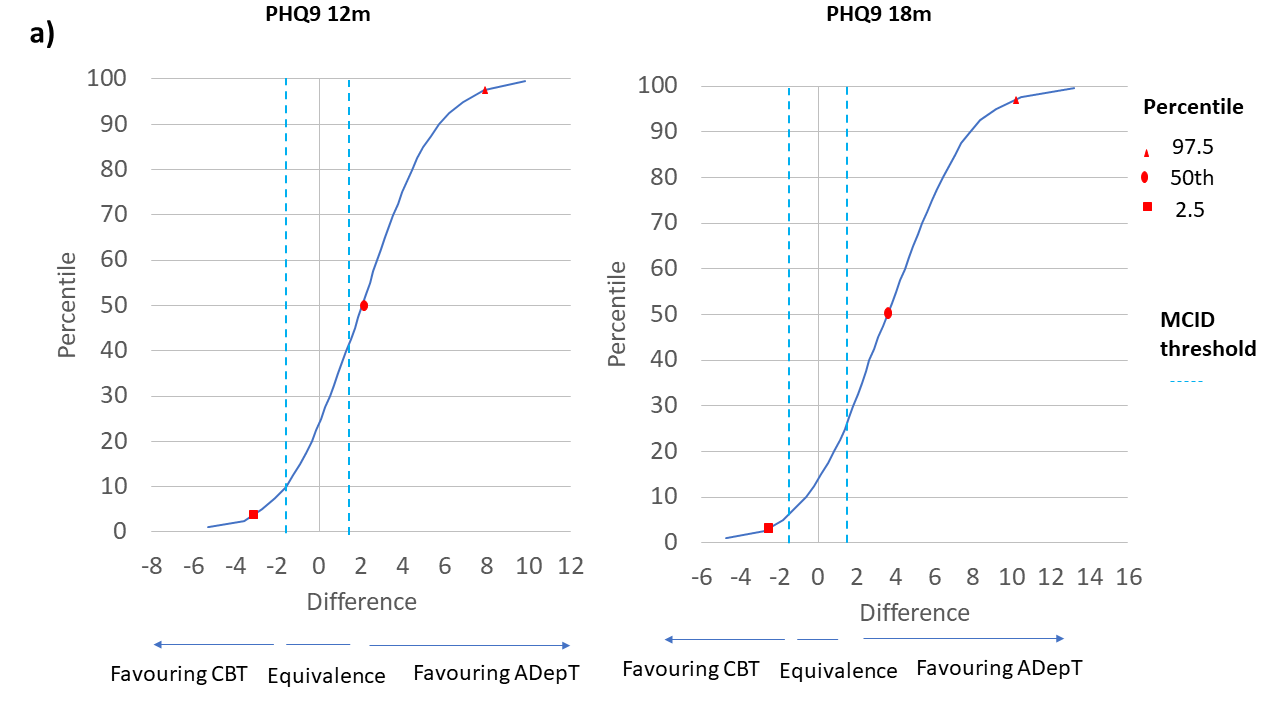


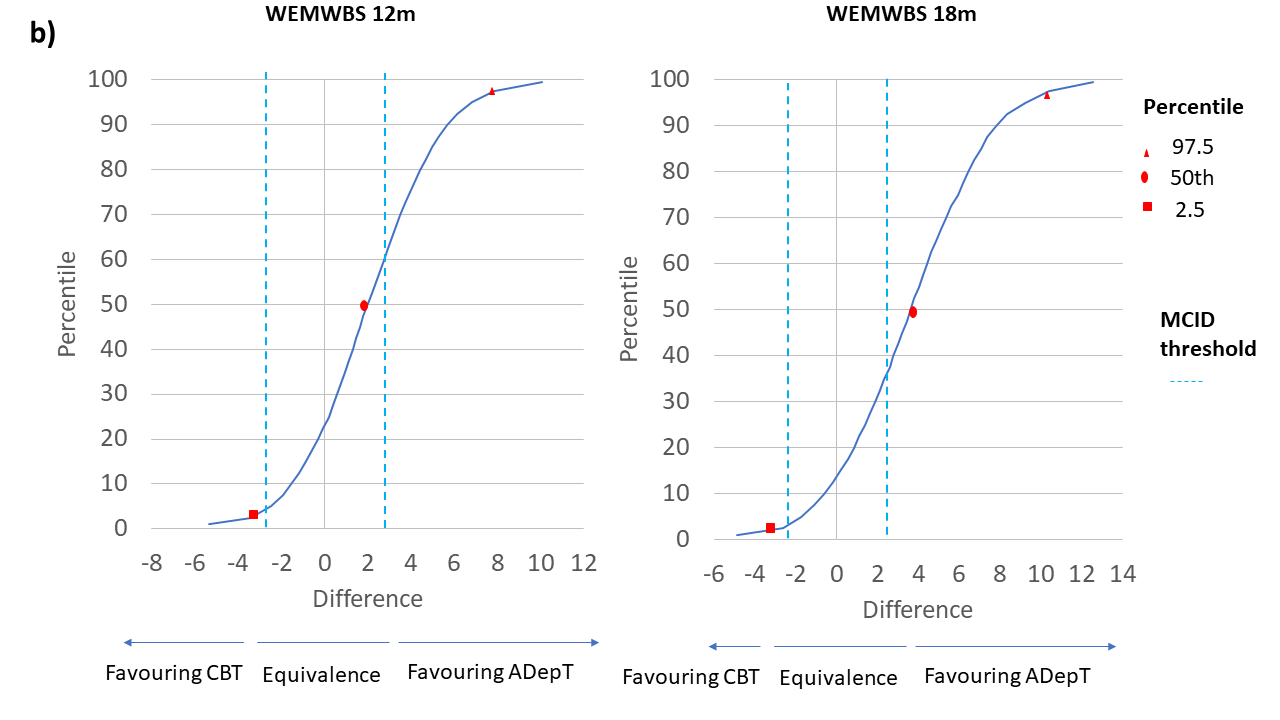


*Note:* Analyses are run on bootstrapped distribution generated from complete case data. Red shapes indicate the Bayesian 95% credibility interval. Blue dotted lines indicate the minimum clinically important difference threshold (MCID; 1·7 points for PHQ-9, 2·8 points for WEMWBS). Where curves cross the right hand blue dotted line, ADepT is MCID superior to CBT; where curves cross the left hand blue dotted line, CBT is MCID superior to ADepT. Where curves cross to the right of zero, ADepT is superior to CBT at any value; where curves cross to the left of zero, CBT is superior to ADepT at any value.

**6: Additional Health Economic Proof-of-Concept Analyses**

Table S16 provides a detailed breakdown of service use by category. There was minimal imbalance in service use between arms in any of the resource categories, apart from a pattern of greater ambulance call outs in the CBT relative to the ADepT arm. Table S17 and Figure S6 reports the results of the cost-effectiveness sensitivity analysis, examining if a similar pattern emerged if focusing on six-month outcomes. In terms of data completeness 34/41(83%) of CBT and 31/41(76%) of ADepT participants had full cost and QALY data for the six-month analyses. The sensitivity analysis continued to suggest ADepT is cost-effective relative to CBT, with clearer evidence when focusing on the ICECAP than the EQ-5D QALY measure.

Table S16

Service use (unit) in each arm during the trial follow-up broken down by category.

|  | *CBT* |  | *ADepT* |  |
| --- | --- | --- | --- | --- |
| Service use | Mean (SD) | Used >1 | Mean (SD) | Used>1 |
| Inpatient stays (nights) | 0.09 (0.39) | 2/32 | 0.03 (0.18) | 1/30 |
| Outpatient appointments (attendances) | 1.28 (2.12) | 16/32 | 0.97 (1.46) | 14/30 |
| Accident and emergency (attendances) | 0.40 (0.76) | 8/32 | 0.65 (1.85) | 7/30 |
| Ambulance (calls) | 0.16(0.37) | 5/32 | 0.07 (0.36) | 1/32 |
| GP contacts (practice, home visit, phone) | 9.64 (7.49) | 31/32 | 7.85 (7.50) | 28/30 |
| Other community or social care services | 10.89 (13.69) | 27/32 | 8.12 (9.00) | 22/30 |

*Note:-* Table reports complete case data; Used>1 reports the number of individuals making use of that service at least once during the follow-up period.

Table S17

Cost-effectiveness (6m) sensitivity analyses.

|  | CBT | ADepT | Difference  (ADepT – CBT) |
| --- | --- | --- | --- |
| **6-month** |  |  |  |
| Total Costs | £2531 (£418) | £2368 (£502) | -£159 (£-379, £60) |
| EQ‑5D‑3L | 0.307 (0.100) | 0.312 (0.105) | 0.008 (-0.018. 0.034) |
| ICECAP | 0.288 (0.066) | 0.298 (0.097) | 0.014 (-0.007, 0.035) |

*Note:-* Cost and QALY estimate are mean (SD) complete case data; difference scores are mean (95% CI) estimates from (unimputed) regression models.

Figure S6

Six-month sensitivity analyses


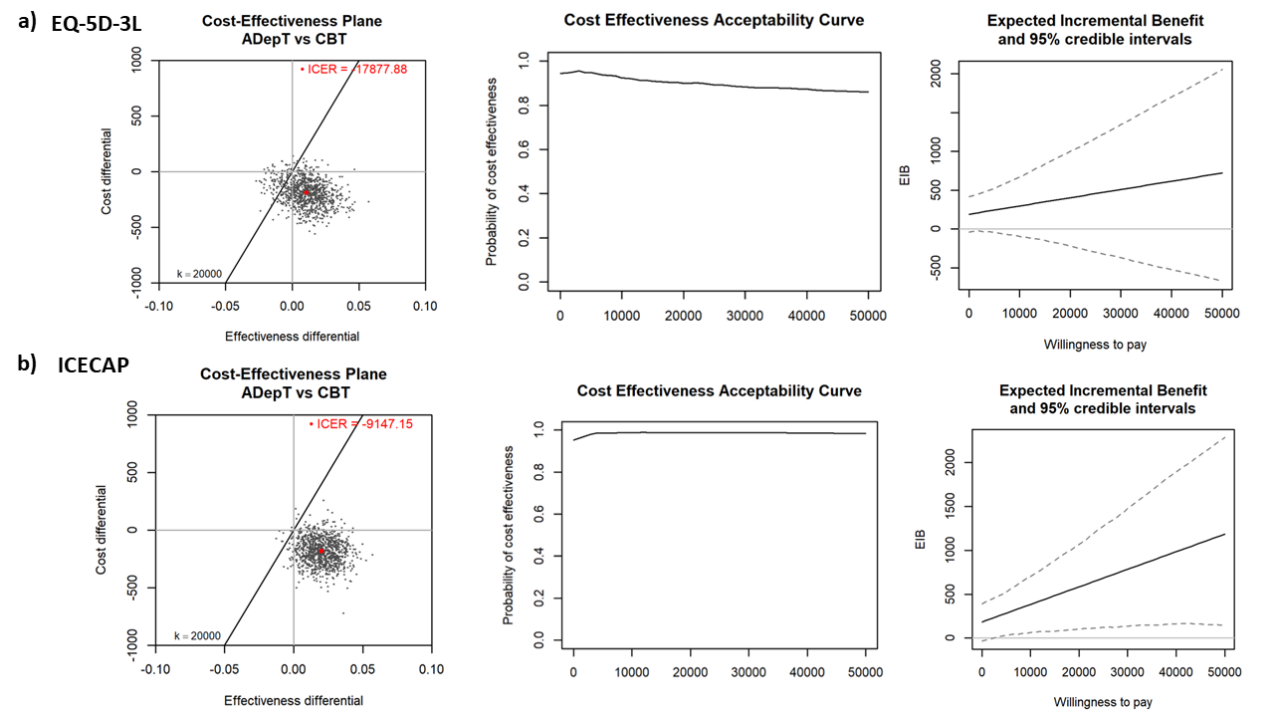


*Note:-* Analyses run on bootstrapped distributions from complete case data. In each panel, the left-hand graph is a scatterplot of cost and effectiveness pairs for ADepT versus CBT on a cost-effectiveness plane (with a £20000 willingness to pay threshold), the middle graph is an expected incremental benefit (EIB) plot showing the monetary benefit of ADepT relative to CBT at different willingness to pay thresholds (with 95% credibility intervals), and the right-hand graph is a cost-effectiveness acceptability curve (CEAC) showing the probability ADepT is cost-effective relative to CBT at different willingness to pay thresholds. A negative ICER value in this context reflects lower costs and greater QALYs in ADepT relative to CBT.

References

1 Thabane, L., Ma, J., Chu, R., Cheng, J., Ismaila, A., Rios, et al.. A tutorial on pilot studies: the what, why and how. *BMC Med Res Methodol* 2010: 10,1.

2 Lee, E., Whitehead, A., Jacques, R., & Julious, S. The statistical interpretation of pilot trials: should significance thresholds be reconsidered? *BMC Med Res Methodol*,2014: 14, 41.

3 Sim J. **Should treatment effects be estimated in pilot and feasibility studies?**Pilot Feasibility Stud, 2019: Article 107, vol. 5(1)

4 IBM Corp (2021). IBM SPSS Statistics for Windows (Version 28.0). IBM Corp, Armonk, NY.

5 R Core Team. R: A language and environment for statistical computing. R Foundation for Statistical Computing, Vienna, Austria. 2018 URL <https://www.R-project.org/>.

6 van Buuren, S., Groothuis-Oudshoorn, K. MICE: Multivariate Imputation by Chained Equations in R. Journal of Statistical Software, 2011: 45(3), 1—67. 10.18637/jss.v045.i03

7 Bland, J. & Altman, D. Comparisons against baseline within randomised groups are often used and can be highly misleading. *Trials*, 2011: 12, 264.

8 von Hippel, P. T., & & Bartlett, J. Maximum likelihood multiple imputation: Faster imputations and consistent standard errors without posterior draws. 2019 [arXiv:1210.0870](https://arxiv.org/abs/1210.0870)

9 Kounali, D., Button, K. S., Lewis, G., Gilbody, S., Kessler, D., Araya, A., et al. How much change is enough? Evidence from a longitudinal study on depression in UK primary care. *Psych Med*, 2020: 52, 1875-1882.

10 Maheswaran, H., Weich, S., Powell, J., & Stewart-Brown, S. Evaluating the responsiveness of the Warwick Edinburgh Mental Well-Being Scale (WEMWBS): group and individual level analysis. *Health Qual Life Outcomes*, 2012: 10, 156.

11 Hengartner, M. & Ploderl, M. Estimates of the minimal important difference to evaluate the clinical significance of antidepressants in the acute treatment of modertate-to-severe depression. *BMJ EBM*, 2021, 27: 69-73

12 Cuijpers, P., Turner, E. H., Koole, S. L., van Dijke, A., & Smit, F.. What is the threshold for a clinically relevant effect? The case of major depressive disorders. *Depress Anx*, 2014: 31, 374-378.

13 Dunn, B. D., Widnall, E., Reed, N., Taylor, R., Owens, C., Spencer, A., et al. Evaluating Augmented Depression Therapy (ADepT): study protocol for a pilot randomised controlled trial. *Pilot Feasibility Stud*, 2019: *5*, 63.

14 Jacobson, N. & Truax, P. Clinical significance: a statistical approach to defining meaningful change in psychotherapy research. *J Consult Clin Psychol*,1991: 59, 12-19.

15 Dunn, B. D., German, R. E., Khazanov, G., Xu, C. L., Hollon, S. D., & DeRubeis, R. J. Changes in Positive and Negative Affect During Pharmacological Treatment and Cognitive Therapy for Major Depressive Disorder: A Secondary Analysis of Two Randomized Controlled Trials. *Clin Psych Sci*, 2020: *8*(1), 36-51.

16 Alsayednasser, B., Widnall, E., O’Mahen, H., Wright, K., Warren, F., Ladwa, A. et al. How well do cognitive behavioural therapy (CBT) and behavioural activation (BA) for depression repair anhedonia: A secondary analysis of the COBRA randomised controlled trial. *Behav Res & Ther,* 2022: 159, 10418.

17 Wiles, N.J, Thomas, L., Abel, A., Barnes, M., Carroll, F., Rigway, N., et al.Cognitive behavioural therapy as an adjunct to pharmacotherapy for primary care based patients with treatment resistant depression: results of the CoBalT randomosed controlled trial. *Lancet,* 2014: 381, 375-384.

18 Richards, D. A., Ekers, D., McMillan, D., Taylor, R. S., Byford, S., Warren, F. C., et al. Cost and Outcome of Behavioural Activation versus Cognitive Behavioural Therapy for Depression (COBRA): a randomised, controlled, non-inferiority trial. *Lancet*, 2016: 388(10047), 871-880.

19 Goodrich B, Gabry J, Ali I & Brilleman S. (2020). rstanarm: Bayesian applied regression modeling via Stan. R package version 2.21.1 <https://mc-stan.org/rstanarm>.

20 Clements, M. N., White, I. R., Copas, A. J., Cornelius, V., Cro, S., Dunn, D. T., et al. .. Improving clinical trial interpretation with AccEPT analyses. *NEJM Evid*, 2022: 1 (8).

21 Clarke, P. When can group level clustering be ignored? Multilevel models versus single-level models withsparse data. *J Epidemiol and Community Health*, 2008, 62, 752-758.

22 Dolan PGC, Kind P, Williams A. A Social Tariff for EuroQoL: Results from a UK General Population Survey. York: University of York; 1995.

23 Flynn, T. N., Huynh, E., Peters, T. J., Aljanabi, H., Clemens, S., Moody, A., & Coast, J. . Scoring the ICECAP-A capability instrument: estimation of a UK general population tariff. *Health Econ*, 2013: 24, 258-269.

24 Hernandez-Alava , M., Pudney, S., & Wailoo, A. Estimating the relationship between the EQ-5D-5L and EQ-5D-3L: Results from a UK population study. *Pharmacoeconomics*, 2023: 41: 199-207.

25 Netten A, Knight J, Dennett J, Cooley R, Slight A. A ‘Ready Reckoner’ for Staff Costs in the NHS,Volume I, Estimated Costs. Canterbury: University of Kent; 1998.

26 Curtis, L. A., & Burns, A. Unit Costs of Health and Social Care 2019. Unit Costs of Health and Social Care . PSSRU, University of Kent, 176, 2019 ISBN 978-1-911353-10-2.

27 National Schedule of NHS Costs 2018/2019. Accessed at: <https://www.england.nhs.uk/publication/2018-19-national-cost-collection-data-publication/>

28 Joint Formulary Committee. British National Formulary 77. BMJ Publishing and the Royal Pharmaceutical Society, 2019.

29 Canty, A., & Ripley, B. D. . *boot: Bootstrap R (S-Plus) Functions*. 2021

30 Baio, G, A Berardi, and A Heath. Bayesian Cost-Effectiveness Analysis with the r Package BCEA. New York, NY: Springer, 2017.

31 Devilly, G. J., & Borkovec, T. D. . Psychometric properties of the credibility/expectancy questionnaire. *JBehav Ther Exp Psychiatry*, 2000: 31(2), 73-86.

32 Blackburn, I. M., James, I. A., Milne, D. L., Baker, C., Standart, S., Garland, A., & Reichelt, F. K.The Revised Cognitive Therapy Scale (Cts-R): Psychometric Properties. *Behav Cog Psychotherapy*, 2001: 29(4), 431-446.

33 Dunn, B. D., Widnall, E., Reed, N., Owens, C., Campbell, J., & Kuyken, W. Bringing light into darkness: A multiple baseline mixed methods case series evaluation of Augmented Depression Therapy (ADepT). *Behav Res Ther*, 2019: 120, 103418

34 Cuijpers, P., Karyotaki, E., Weitz, E., Andersson, G., Hollon, S. D., & van Straten, A. The effects of psychotherapies for major depression in adults on remission, recovery and improvement: A meta-analysis. *JAffect Disord*, 2014: 159, 118-126.
